# New Esters of Ferrediol and Sideritriol in *Sideritis clandestina* subsp. *peloponnesiaca*: Characterization and Antiproliferative Activity

**DOI:** 10.1021/acsomega.4c04628

**Published:** 2024-10-04

**Authors:** Virginia D. Dimaki, Efstathia Stamopoulou, Dimitra Manou, Achilleas D. Theocharis, Manolis A. Fousteris, Fotini N. Lamari

**Affiliations:** † Department of Pharmacy, University of Patras, Patras 26504, Greece; ‡ Department of Chemistry, University of Patras, Patras 26504, Greece

## Abstract

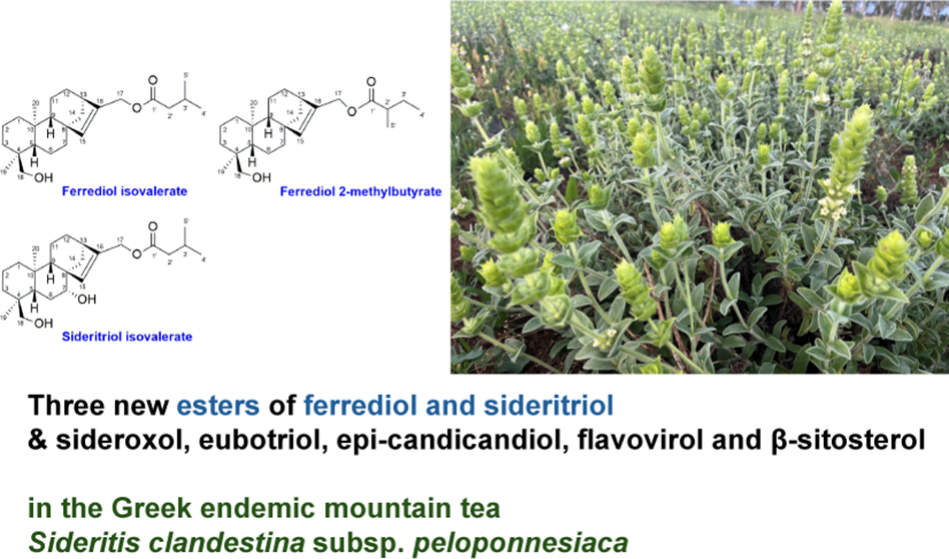

17-Isovalerate esters of ferrediol and sideritriol (compounds **1** and **5**) and 17-(2-methylbutyrate) ferrediol (compound **2**), which have not been earlier described, were isolated along with five known *ent*-15-kaurene diterpenes (siderol, sideridiol, sideroxol, eubotriol, and *epi*-candicandiol), one *ent*-beyerene (flavovirol), and one phytosterol (β-sitosterol) from the aerial parts of *Sideritis clandestina* subsp. *peloponnesiaca* (Boiss. & Heldr.) Baden that is a mountain tea species endemic to Northern Peloponnese in Greece. Compounds **1**–**5** exhibited weak antiproliferative activity against triple-negative MDA-MB-231 and Hs578T breast cancer cells. In MCF-7 breast cancer cells, compounds **1**, **2**, siderol, and sideridiol were equipotent to 5-fluorouracil (moderate antiproliferative activity).

## 1 Introduction

The genus *Sideritis* (family Lamiaceae) consists of more than 150 species, predominantly found in the Mediterranean region.^1^ Most *Sideritis* species, commonly known as “mountain tea”, have traditionally been consumed as herbal teas for their distinct organoleptic and therapeutic properties, such as alleviating symptoms of cough, common cold, pain, asthma, gastrointestinal disorders, and mild anxiety. *Sideritis* species are a remarkable source of diterpenes. Over 160 diterpenes have been identified in various *Sideritis* species and classified into several types according to their carbon skeletons, including *ent*-kaurane, labdane, atisane, pimarane, beyerane, trachilobane, and rosane.^1−3^ Diterpenes from *Sideritis* exhibit various biological properties, including antibacterial, antifungal, antiviral, anticholinesterase, anti-inflammatory, neuroprotective, and cytotoxic activities.^4−11^*Sideritis* species from the Eastern and Central Mediterranean areas (Turkey, Greece, and Italy) are characterized by the presence of *ent*-kaurene derivatives.^1,3,12^ Previous studies have demonstrated the cytotoxic activity of siderol, the most characteristic and abundant *ent*-kaurene in *Sideritis*.^9,10^ Those findings, along with the evaluation of other *ent*-kaurenes in cancer cell lines,^6−8^ highlight the antiproliferative activity of *Sideritisent*-kaurenes and emphasize the need for further evaluation of structure–activity relationships.

In this study, we report the isolation of three new *ent*-kaurene diterpene esters from *Sideritis clandestina* subsp. *peloponnesiaca* (Boiss. & Heldr.) Baden for the first time. *S. clandestina* subsp. *peloponnesiaca* is a taxon that is endemic to the mountains of the Central and Northern Peloponnese. Available data on its phytochemical composition are limited. We have previously reported its essential oil composition and its polar metabolites content (iridoid glycosides, vanillic and salicylic acid glycosides, chlorogenic acid, and phenylethanoid glycosides),^13,14^ while we have described the isolation of siderol and sideridiol from its aerial parts and the anti-Aβ aggregation mechanism for sideridiol.^11^ Only one earlier publication has reported the isolation and characterization of *ent*-15-kaurenes from *Sideritis theezans* Boiss. & Heldr. (synonym of the other endemic subspecies *Sideritis clandestina* (Bory & Chaub.) Hayek subsp. *clandestina*), that is, siderol, isolinearol, isosidol, sideridiol, epoxy-isolinearol, isofoliol, and isosidol.^15^

**Figure 1:**
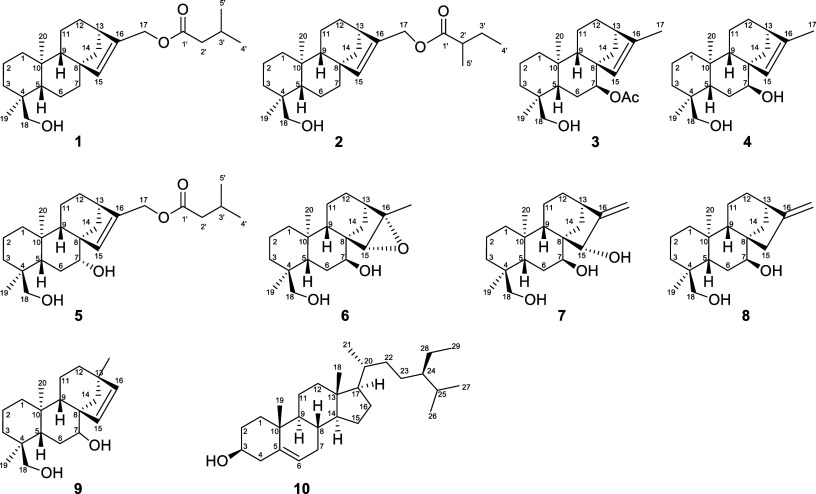
Chemical structures of isolated compounds **1**–**10**.

Herein, we embarked on the full investigation of diterpenes from *S. clandestina* subsp. *peloponnesiaca* and present the characterization of three new *ent*-15-kaurene esters, 17-isovalerate and 17-(2-methylbutyrate) of ferrediol, and (**1**, **2**) and 17-isovalerate of sideritriol (**5**) that have not been described earlier either in *Sideritis* or in any other species (Figure 1). Furthermore, we document for the first time the presence of (i) three known *ent*-15-kaurenes, that is, sideroxol (**6**), eubotriol (**7**), and 7-epicandicandiol (**8**), (ii) one *ent*-15-beyerene, that is, flavovirol (**9**), and (iii) one phytosterol, that is, β-sitosterol (**10**) in *S. clandestina* subsp. *peloponnesiaca* aerial parts. We also present the evaluation of the antiproliferative activity of compounds **1**–**5** on MCF-7, MDA-MB-231, and Hs578T breast cancer cell lines.

## 2 Results and Discussion

### 2.1 Isolation and Characterization of Compounds **1**–**10**

The dried aerial parts of *S. clandestina* subsp. *peloponnesiaca* were extracted sequentially with petroleum ether, ethyl acetate (EtOAc), and methanol (MeOH) at room temperature. The EtOAc extract, after being concentrated under reduced pressure, was fractionated into five fractions (Fr.A–Fr.E) by using silica gel column chromatography. Fraction B was further purified using silica gel column chromatography, preparative thin-layer chromatography (PTLC), and high-performance liquid chromatography (HPLC), yielding three new *ent*-15-kaurenes (**1**, **2**, and **5**), five known *ent*-15-kaurene diterpenes (**3**, **4**, and **6**–**8**), one *ent*-beyerene (**9**), and one phytosterol (**10**). The experimental steps are summarized in Scheme S1 and described in detail in Section 3.3.

Compound **1** was obtained as a colorless oil. HRESIMS analysis revealed a sodium adduct ion at *m*/*z* 411.2870 [M + Na]^+^ (calcd for C_25_H_40_O_3_Na, 411.2870) suggesting a molecular formula of C_25_H_40_O_3_, and six degrees of unsaturation (Figure S1). The IR spectrum showed prominent absorption bands for hydroxyl groups at 3428 cm^–1^ and carbonyl groups at 1734 cm^–1^ (Figure S2).

Several parallels in the ^1^H and ^13^C NMR spectroscopic data resonances were identified with those of *ent*-kaurene diterpenes (Table 1, Figures S3–S9). Specifically, a proton signal at δ_H_ 2.54–2.55 (1H, m, H-13) with its characteristic multiplicity was observed, and the presence of an endocyclic double bond (C-15 and C-16) was inferred from the olefinic proton signal at δ_H_ 5.42 (1H, s, H-15) and δ_C_ 139.90 (CH, C-15). In addition to the two distinctive *ent*-kaurene methyl protons at δ_H_ 0.77 (3H, s, H-19) and δ_H_ 1.09 (3H, s, H-20), the NMR spectra revealed two additional methyl signals at δ_H_ 0.96 (6H, d, H-4′ and H-5′) and δ_C_ 22.45 (CH_3_, C-4′ and C-5′), which were not correlated to the *ent*-kaurenoid subunit, as confirmed by the 2D NMR spectra. Additionally, the ^13^C NMR data and APT experiment demonstrated a total of 25 carbon signals, including four methyl carbons, 11 methylene carbons, five methine carbons (one sp^2^), four quaternary carbons (one sp^2^), and one ester carbonyl carbon at δ_C_ 173.04 (C-1′).

**Table 1:** NMR Spectroscopy Data (700 MHz for ^1^H and 176 MHz for ^13^C) for Ferrediol Isovalerate and Ferrediol 2-Methylbutyrate (**1** and **2**) in CDCl_3_ and (600 MHz for ^1^H and 150 MHz for ^13^C) for Sideritriol Isovalerate (**5**) in CD_3_OD

	**1**			**2**			**5**		
position	δ_C_, type	δ_H_ (*J* in Hz)	HMBC^a^	δ_C_, type	δ_H_ (*J* in Hz)	HMBC^a^	δ_C_, type	δ_H_ (*J* in Hz)	HMBC^a^
1a	39.96, CH_2_	1.82, d (12.8)	2, 3, 5, 10, 20	39.95, CH_2_	1.82, d (12.8)	2, 3, 20	41.12, CH_2_	1.82, d (13.0)	3, 5
1b		0.79–0.76, m	2, 3, 4, 5		0.79–0.76, m	2, 3, 4, 5, 20		0.82, td (3.5, 12.6)	2, 3, 10, 20
2a	17.93, CH_2_	1.68–1.62, m^b^	1, 3	17.93, CH_2_	1.69–1.65, m^b^	4, 10	19.04, CH_2_	1.69–1.65, m^b^	3, 4
2b					1.51–1.49, m^b^			1.49–1.42, m^b^	4, 10
3a	35.31, CH_2_	1.42–1.38, m^b^	2	35.31, CH_2_	1.42–1.38, m^b^	19	36.54, CH_2_	1.49–1.42, m^b^	2, 4, 18, 19
3b		1.29–1.27, m^b^			1.29–1.27, m^b^			1.31, s^b^	2, 4, 19
4	37.60, C			37.60, C			38.24, C		
5β	48.95, CH	1.11, dd (1.4, 12.0)	4, 6, 7, 10, 9, 19, 20	48.94, CH	1.12, dd (1.4, 12.0)	1, 4, 6, 10, 18, 19, 20	40.98, CH	1.73–1.71,m	4, 6, 7, 9, 10, 18, 19
6a	38.74, CH_2_	1.68–1.62, m^b^	5, 7, 8, 10	38.74, CH_2_	1.69–1.65, m^b^	5, 8	27.57, CH_2_	1.60–1.55,m^b^	5, 7, 8, 10
6b		1.58–1.55, m^b^			1.61–1.58, m^b^				
7	18.88, CH_2_	1.33–1.29, m^b^	6, 8	18.88, CH_2_	1.32–1.29, m^b^	5, 8	75.66, CH	3.55, s	5, 6, 8, 9, 14
8	49.08, C			49.07, C			54.51, C		
9β	48.14, CH	1.09–1.06, m^b^	11, 14, 15, 20	48.16, CH	1.09–1.06, m^b^	11, 14, 15, 20	44.63, CH	1.41–1.38,m^b^	7, 8, 11, 12, 13
10	39.33, C			39.33, C			40.56, C		
11	25.52, CH_2_	1.54–1.52, m^b^	8, 9, 13	25.56, CH_2_	1.56–1.53, m^b^	8, 10, 13	26.43, CH_2_	1.60–1.55,m^b^	13
12	18.61, CH_2_	1.58–1.55, m^b^	9, 11, 13, 14	18.61, CH_2_	1.61–1.58, m^b^	10, 11, 13	19.35, CH_2_	1.60–1.55,m^b^	11
13α	41.66, CH	2.55–2.54, m	8, 11, 12, 14, 15, 16	41.70, CH	2.55–2.54, m	8, 12, 14, 15, 16	42.92, CH	2.56, s	8, 12, 14, 15, 16
14a	43.80, CH_2_	2.15–2.10, m^b^	8, 11, 13, 15, 16	43.84, CH_2_	2.04, d (10.2)	9, 11, 13, 15, 16	43.42, CH_2_	2.04, d (10.1)	8, 9, 11,13, 15, 16
14b		1.42–1.38, m^b^	8, 11, 13		1.42–1.38,m^b^	8, 9, 13		1.41–1.38,m^b^	7, 8, 9, 11, 12, 13
15	139.90, CH	5.42, s	8, 13, 14, 16, 17	138.81, CH	5.42, s	6, 8, 9, 13, 14, 16, 17	135.66, CH	5.88, s	8, 14, 16, 17
16	141.00, C			141.08, C			143.03, C		
17	62.16, CH_2_	4.64, qd (1.4, 13.3)	13, 15, 16, 1′, 2’	62.18, CH_2_	4.64, qd (1.4, 13.3)	13, 15, 16, 1’	63.24, CH_2_	4.65, qd (1.5, 13.5)	13, 15, 16, 1’
18a	72.15, CH_2_	3.42, d (10.9)	3, 4, 5, 19	72.15, CH_2_	3.43, d (10.9)	3, 4, 5, 19	72.37, CH_2_	3.29^c^	3, 4, 5, 19
18b		3.10, d (10.9)	3, 4, 5, 19		3.11, d (10.9)	3, 4, 5, 19		3.06, d (11.2)	3, 4, 5, 19
19	17.44, CH_3_	0.77, s	2, 3, 4, 5, 18	17.43, CH_3_	0.77 s	2, 3, 4, 5, 18	17.63, CH_3_	0.76, s	3, 4, 5, 18
20	18.06, CH_3_	1.09, s	1, 5, 9, 10	18.06, CH_3_	1.09, s	1, 5, 8, 9	18.50, CH_3_	1.11, s	1, 5, 9, 10
1’	173.04, C=Ο			176.60, C=Ο			174.62, C=O		
2’	43.46, CH	2.22, d (7.0)	1′, 3′, 4′, 5′	41.16, CH	2.40, sextet (7.0)	1′, 3′, 4′, 5′	44.25, CH_2_	2.21, d (7.1)	1′, 3′, 4′, 5′
3′	25.70, CH_2_	2.15–2.10, m^b^	2′, 4′, 5′	26.79, CH_2_	1.72–1.68, m^b^	1′, 2′, 4′, 5′	26.89, CH	2.10–2.04, m^b^	1′, 2′, 4′, 5′
4’	22.45, CH_3_	0.96, d (6.3)	3′, 5′	11.66, CH_3_	0.92, t (7.5)	2′, 3′,	22.70, CH_3_	0.95, d (6.7)	2′, 3′, 5′
5′	22.45, CH_3_	0.96, d (6.3)	3′, 4’	16.66, CH_3_	1.16, d (7.0)	1′, 2′, 3′,	22.70, CH_3_	0.95, d (6.7)	2′, 3′, 4’

a HMBC correlations are from proton(s) stated to the indicated carbon.

b Overlapped signals.

c Overlapping with solvent.

**Figure 2:**
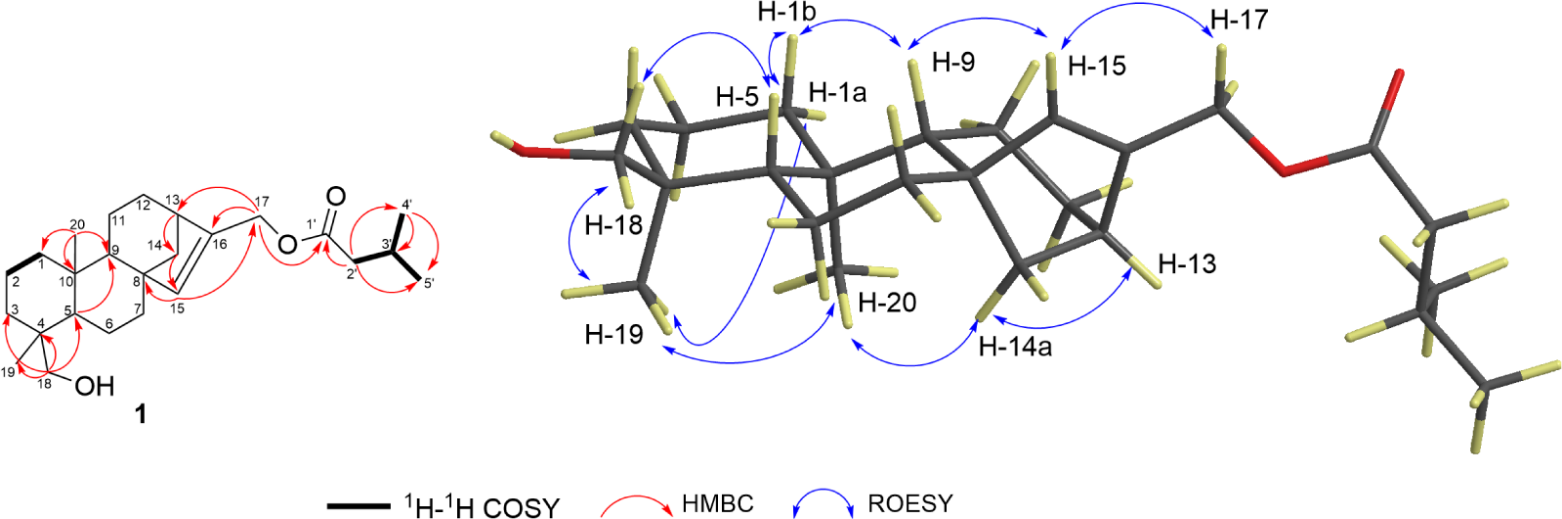
Important ^1^H–^1^H COSY, HMBC, and ROESY correlations of compound **1**.

HMBC and COSY correlations (Table 1, Figures 2 and S7) established the 6/6/6/5 ring system (A/B/C/D) that characterizes all *ent*-kaurenoid structures.^16^ The six-membered ring A was deduced by the HMBC correlations between H_3_-20 (δ_H_ 1.09) and C-1, C-5, C-9, and C-10; H_3_-19 (δ_H_ 0.77) and C-3, C-4, C-5, and C-18; H_2_-18 and C-3, C-4, C-5, and C-19; and H-5 and C-4, C-6, C-9, C-10, and C-19 and also by the correlations observed in the COSY spectrum between H_2_-1 and H_2_-2. The chemical shift of H_2_-18 (Ha δ_H_ 3.42, d, *J* = 10.9 Hz and Hb 3.10, d, *J* = 10.9 Hz) indicates the linkage to a hydroxyl group, further supported by the IR absorption peak at 3428 cm^–1^. Furthermore, the HMBC correlations from H-9 (δ_H_ 1.06–1.09, m) to C-11, C-14, C-15, and C-20; from H-13 (δ_H_ 2.54–2.55, m) to C-8, C-11, C-12, C-14, C-15, and C-16; and from H_2_-14 to C-8, C-13, C-15, and C-16 confirmed the presence of rings B and C. Finally, the five-membered ring D was identified by HMBC correlations of the deshielded olefinic H-15 (δ_H_ 5.42, s) with C-8, C-13, C-14, C-16, and C-17, as well as by 4-bond COSY correlations between H-15 and both H_2_-17 and H_2_-14a.

Regarding the two additional secondary methyl groups, signals at δ_H_ 0.96 (6H, d, *J* = 6.3 Hz, CH_3_-4′ and CH_3_-5′) and δ_C_ 22.45 (CH_3_, C-4′ and C-5′) along with their corresponding HMBC and COSY correlations confirmed the presence of two equivalent terminal methyl groups (Figures S7 and S8). HMBC correlations of the methyl protons H_3_-4′ and H_3_-5′ with C-3′ and of H_2_-2′ (δ_H_ 2.22, d, *J* = 7 Hz) with C-3′, C-4′, C-5′, and the ester carbonyl C-1′ (δ_C_ 173.04), along with the proton resonance of H_2_-17 (δ_H_ 4.64, qd, *J* = 1.4, 13.3 Hz), suggested that an isovalerate ester group was present. The existence of the ester group was further corroborated by the IR absorption band at 1734 cm^–1^. Last, HMBC correlations of H_2_-17 with C-13, C-15, C-16, C-1′, and C-2′ via 4-bond correlation established the location of the ester linkage at C-17 of the *ent*-15-kaurene moiety.

Analysis of the ROESY spectrum provided insights into the relative configuration of compound **1** (Figures 2 and S9). In the ROESY spectrum of compound **1**, H-5 correlated with H-1b, H-9 correlated with both H-15 and H1b, and H-15 correlated with H-17, indicating that H1b, H-5, H-9, H-15, and H-17 share cofacial relationships. Given the β-orientation assigned to H-5, it is inferred that H-1b, H-5, H-9, and H-17 are β-oriented. ROESY correlations showed that H_3_-19 interacts with both H_3_-20 and H-1a, while H-14a correlates with H-13 and H_3_-20, indicating that H-1a, H-13, H-14a, H_3_-19, and H_3_-20 are α-oriented. Additionally, H_2_-18 correlated with both H-5 and H_3_-19. Consequently, compound **1** is assigned as *ent*-17-isovaleroxy-18-hydroxykaur-15-ene. The *ent*-17,18-dihydroxykaur-15-ene (ferrediol) was isolated from *Sideritis ferrensis* for the first and only time,^17^ whereas compound **1** represents a new ester of it, the 17-isovalerate of ferrediol.

**Figure 3:**
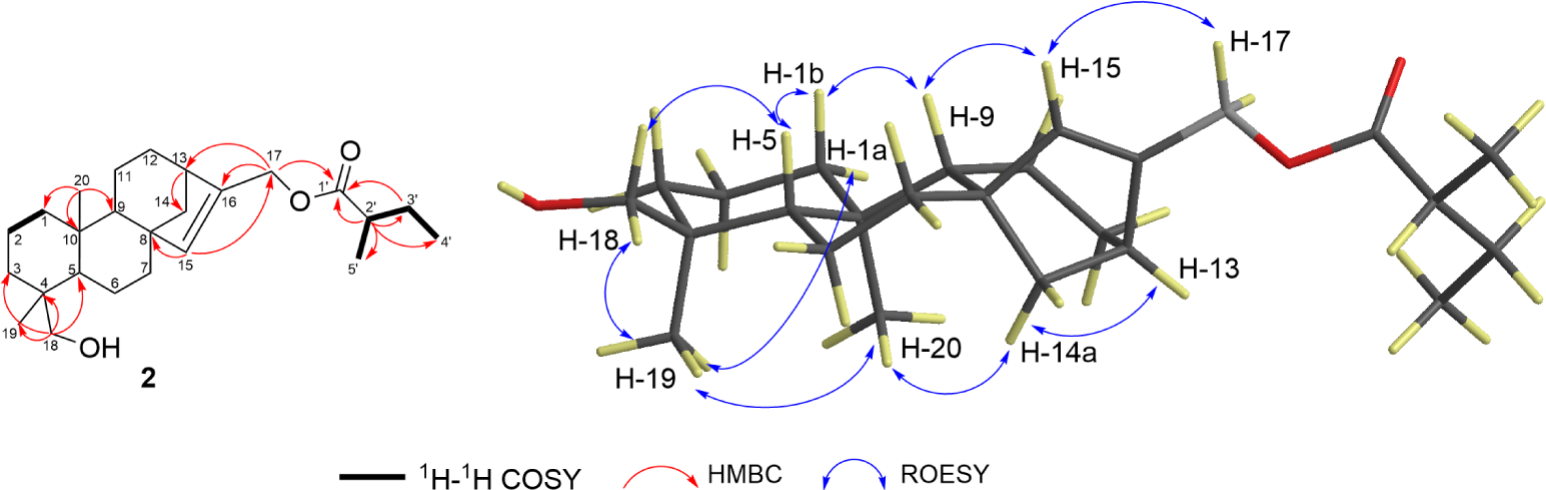
Important ^1^H–^1^H COSY, HMBC, and ROESY correlations of compound **2**.

Compound **2** was a colorless oil with the molecular formula C_25_H_40_O_3_, as determined by HRESIMS analysis. The sodium adduct ion appeared at *m*/*z* 411.2865 [M + Na]^+^ (calcd for C_25_H_40_O_3_Na, 411.2870), indicating six degrees of unsaturation (Figure S10). Analysis of 1D and 2D NMR, as well as IR spectra (Table 1, Figures 3 and S11–S18), confirmed the close structural similarity of compound **2** and compound **1**, since the key ^1^H and ^13^C NMR signals attributed to their ferrediol moiety were superimposable. The only difference was the presence of a terminal 2-methylbutyrate ester group at C-17 on the *ent*-kaurene skeleton. This conclusion was supported by the ^1^H NMR signals and *J*-couplings of H-2′ (δ_H_ 2.40, sex, *J* = 7 Hz), H-3′ (δ_H_ 1.72–1.68, m), and H-4′ (δ_H_ 0.92, t, *J* = 7.5 Hz) and of H-5′ (δ_H_ 1.16, d, *J* = 7 Hz). The relative configuration of compound **2** was determined to be identical to that of compound **1**, based on diagnostic ROESY correlations (Figure 3). Therefore, compound **2** is identified as *ent*-17-(2′-methylbutyryloxy)-18-hydroxykaur-15-ene and is named 17-(2′-methyl)butyrate of ferrediol.

**Figure 4:**
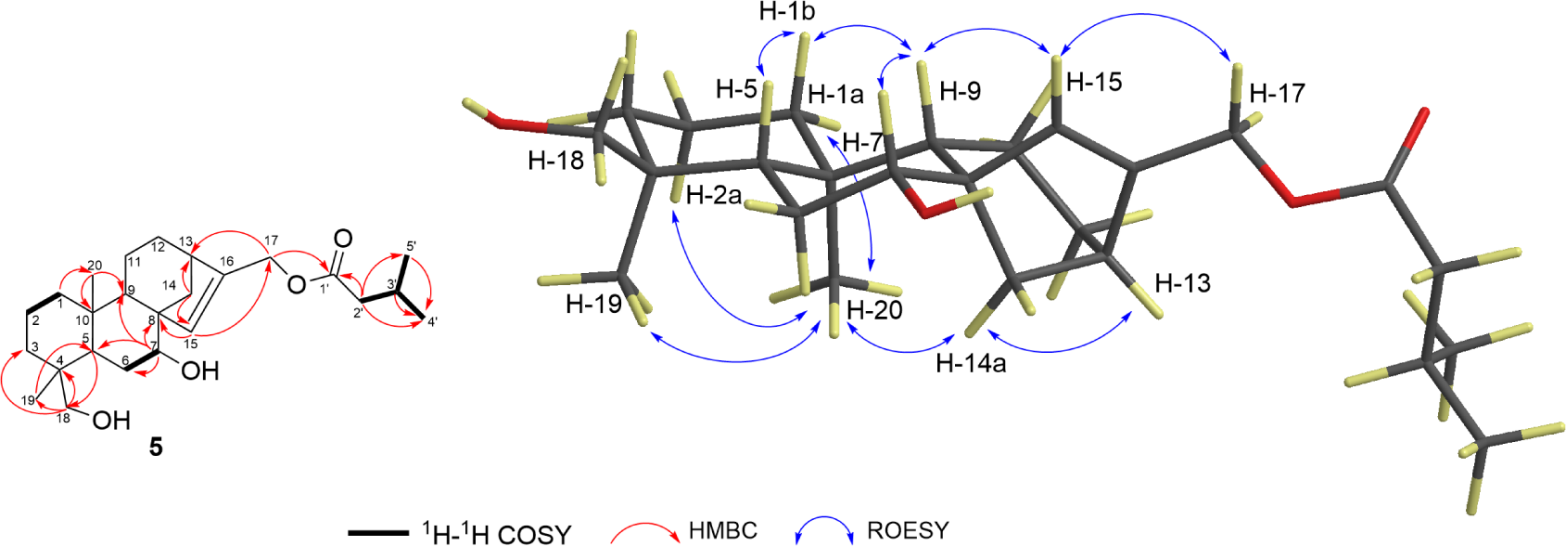
Important ^1^H–^1^H COSY, HMBC, and ROESY correlations of compound **5**.

Compound **5** was isolated as a colorless oil. Its molecular formula, C_25_H_40_O_4_, was based on the HRESIMS spectrum, which displayed a sodium adduct ion at *m*/*z* 427.2808 [M + Na]^+^ (calcd for C_25_H_40_O_4_Na, 427.2819), indicating six degrees of unsaturation (Figure S19). Characteristic absorption bands for hydroxyl groups at 3370 cm^–1^ and carbonyl groups at 1736 cm^–1^ were observed in the IR spectrum (Figure S20). A comparison of 1D and 2D NMR spectra revealed significant similarities of compounds **5** and **1** (Table 1, Figures 4, and S21–S27). However, a key difference occurred at position C-7, where the ^1^H NMR signals indicated the presence of a downfield proton (δ_H_ 3.55, s, H-7), suggesting a hydroxyl group substitution in place of the C-7 methylene carbon found in compounds **1** and **2**. The presence of the hydroxyl group was further supported by the 16 amu difference in the sodium adduct ion between compounds **5** and **1**. This conclusion was further confirmed by the ^13^C NMR and APT signals for C-7 (δ_C_ 75.66), which were consistent with an oxymethine carbon. Additionally, HMBC correlations of H-7 with C-5, C-6, C-8, C-9, and C-14, along with COSY correlations between H-6 and H-7, undoubtedly corroborated the incorporation of a hydroxyl at C-7 on the *ent*-kaurenoid subunit. The remaining ^1^H and ^13^C NMR signals were superimposed on those of compound **1**. The relative configuration of compound **5** was inferred to be identical to that of compounds **1** and **2**, based on diagnostic ROESY correlations (Figure 4). In detail, the correlations of H-15/H-9, H-7/H-9, H-5/H-1b, and H-9/H-1b show that the protons H-1b, H-5, H-7, and H-9 have β-orientation. The α-orientation of CH_3_-19, CH_3_-20, H-13, H-14a, H-1a, H-2a, and H-14a was deduced from the correlations H-2a/H_3_-20; H_3_-19/H_3_-20; H-13/H-14a; and H_3_-20/H1a, H_3_-19, and H-14a. Consequently, compound **5** is identified as *ent*-17-isovaleroxy-7α,18-dihydroxykaur-15-ene. The *ent*-7α,17,18-trihydroxykaur-15-ene was first isolated from *Sideritis sicula* and named sideritriol,^18^ and thus, compound **5** is described as 17-isovalerate of sideritriol. As far as we know, only acetate esters of sideritriol have been documented.^1,2,19^

Siderol (**3**) and sideridiol (**4**), *ent*-15-kaurene diterpenoids, have been isolated from numerous species of *Sideritis*.^1^ Although we previously reported the isolation of siderol (**3**) and sideridiol (**4**),^11^ we now present herein their isolation (using a modified protocol for siderol), along with their full spectral data (HRESIMS, 1D and 2D NMR spectra, Table S1 and Figures S28-S43), to complete the *S. clandestina* subsp. *peloponnesiac*a diterpene characterization. The structures of compounds **6**–**10** were determined by ^1^H and ^13^C NMR, supported by literature data,^20−24^ and attached proton test (APT) analysis whenever that was feasible due to the very small isolated quantities (Table S1–S3, Figures S44–S67). Sideroxol (**6**), eubotriol (**7**), 7-epicandicandiol (**8**), flavovirol (**9**), and β-sitosterol (**10**) have been isolated from *S. clandestina* for the first time in this study. It is worth mentioning that flavovirol (*ent*-7,18-dihydroxybeyer-15-ene, **9**) belongs to *ent*-15-beyerenes rather than to *ent*-kaurenes.

### 2.2 Antiproliferative Activity of Compounds **1**–**5**

The antiproliferative activity of compounds **1**–**5** was evaluated in three breast cancer cell lines: estrogen receptor-positive MCF-7 cells, triple-negative MDA-MB-231 cells, and mesenchymal triple-negative Hs578T cells (Table 2) after a 48 h treatment. All tested compounds exhibited weak antiproliferative activity (IC_50_s in the range of 45 to 92 μΜ) in MDA-MB-231 and Hs578T cells. However, in MCF-7 cells, their IC_50_ values were significantly lower (*p* < 0.05), with the exception of sideridiol (**4**). In MCF-7 cells, compounds **1**, **2**, **3**, and **4** were equipotent to 5-fluorouracil (5-FU) with no statistically significant differences, demonstrating the antiproliferative potential of those kaurenes against MCF7 cells.

**Table 2:** Antiproliferative Effects (IC_50_ in μM) of Compounds **1**–**5** on Breast Cancer Cells (*n* = 3)^a^

Compound	MCF-7	MDA-MB-231	Hs578T
Ferrediol isovalerate (**1**)	30.2 ± 13.0^a^	91.4 ± 3.1^b, *^	75.7 ± 17.6^b^
Ferrediol 2-methylisobutyrate (**2**)	28.3 ± 10.9^a^	49.2 ± 11.3^b^	85.0 ± 11.9^c^
Sideritriol isovalerate (**5**)	56.8 ± 11.4^a,*^	85.6 ± 19.9^b^	90.4 ± 6.5^b^
Siderol (**3**)	20.7 ± 3.9^a^	88.8 ± 6.6^b^	68.1 ± 3.5^b, *^
Sideridiol (**4**)	43.3 ± 7.3^a^	59.3 ± 10.1^a^	46.6 ± 10.1^a^
5-Fluorouracil (5-FU)	20.6 ± 3.5^a^	5.0 ± 0.2^a^	6.1 ± 2.1^a^

a The superscript letters denote the statistically significant differences (two-way ANOVA) of the same compound among cell lines: the same letter across columns denotes the absence of significant differences. Superscript asterisks (one-way ANOVA) show significant differences from those of 5-FU in the same cell line.

Similar IC_50_ values were previously determined for siderol (**3**) against the colorectal DLD1, cervical HeLa, lung A549, glioblastoma T98, and U87 human cancer cells (26.4, 44.7, 46, 18, and 13 μΜ, respectively) after 72 h treatment as measured with the MTT assay (the latter two also analyzed using the trypan blue exclusion assay).^9,10^ In particular, siderol, at ΙC_50_ concentrations, induced G0/G1 cell cycle arrest and hindered migration of the glioma T98 and U87 cells.^10^ These observations, combined with our findings, confirm the antiproliferative activity of the *ent*-kaurene family and suggest that siderol (**3**), sideridiol (**4**), and newly isolated ferrediol and sideritriol esters (**1**, **2**, and **5**) may serve as starting points for further optimization into more potent chemotherapeutic agents against breast cancer.

## 3 Experimental Section

### 3.1 General Experimental Procedures

Reversed-phase (RP) HPLC was conducted using a 1260 Infinity II instrument equipped with a DAD detector set to 210 nm from Agilent Technologies, Inc. (Santa Clara, CA, USA) and on an AKTA purifier (GE Healthcare Life Science, Buckinghamshire, United Kingdom) with Unicord 4.12 software (GE Healthcare Life Science). Semipreparative HPLC was performed using Luna C-18(2) (250 mm × 10 mm, 5 μm, 100 Å) from Phenomenex (Torrance, CA, USA). Silica gel (60 Å, 70–230 mesh) from Merck (Darmstadt, Germany) was used for column chromatography (CC). For preparative thin-layer chromatography (PTLC), precoated silica gel plates (20 × 20 cm, 0.5 and 1.0 mm) from Macherey-Nagel (Düren, Germany) were used. Spots were visualized under a UV lamp (254/365 nm) after treatment with SbCl_3_ reagent in CHCl_3_ and heating the plates at 100 °C. IR spectra were acquired using a PerkinElmer (Waltham, MA, USA) FT-IR spectrometer. Optical rotations were measured in CHCl_3_ and MeOH with Schmidt + Haensch (Berlin, Germany) Polartronic Universal at the sodium-D line (589 nm) with a 0.5 dm path-length cell. The results are reported as follows: [α]_D_^26^, concentration (g/100 mL), and solvent. Analysis of compounds **1** and **2** was achieved on an ion-trap HPLC-ESI-MS instrument equipped with the software MassLynx V4.1 (Waters Corporation, USA) on a reversed-phase C-18 Acclaim column (120 Å, 100 × 2.1 mm, particle size 3 μm) from Thermo Fisher Scientific. Analysis of compounds **3**–**10** was conducted using a Single Quadrupole UHPLC- ESI/MS (1260 Infinity II) with OpenLab 3.2 (Agilent Technologies Inc.). Separation was achieved on a reversed-phase Poroshell EC-C-18 column (120 Å, 150 × 4.6 mm, 2.7 μm) from Agilent Technologies, Inc. Mass spectra at high resolution were generated using an LTQ Orbitrap XL 2.5.5 SP1 mass spectrometer from Thermo Fisher Scientific (Waltham, MA, USA). One-dimensional and two-dimensional NMR spectra were recorded on Bruker Avance III HD Ascend spectrometers operating at 700 and 600 MHz (Billerica, MA, USA). Chemical shifts (δ) are reported in parts per million (ppm), with the residual solvent signal serving as an internal standard.

### 3.2 Plant Material

*S. clandestina* subsp. *peloponnesiaca* was collected from Mount Chelmos in July 2017. The aerial parts were air-dried, and voucher specimens were authenticated by Professor Gregoris Iatrou and deposited at the Herbarium of the Department of Biology, University of Patras (UPA 22921).

### 3.3 Extraction and Isolation

An overview of the isolation procedures is provided in Scheme S1. In detail, the dried aerial parts (943 g) were sequentially extracted at room temperature with petroleum ether, ethyl acetate (EtOAc), and methanol (MeOH) with each solvent used five times for 3 days each time, as earlier described.^11,14^ The EtOAc extract was concentrated under reduced pressure to yield 13.01 g. This extract was subjected to silica gel column chromatography (CC_1_) using a series of solvents with increasing polarity: 100% hexane, 100% ethyl acetate, 100% acetone, 100% methanol, and finally a methanol/water mixture (70:30, v/v) with 1% acetic acid. This process resulted in the separation of five fractions (Fr.A–Fr.E), as previously described.^11^ Fr.B (5.56 g) underwent further processing through silica gel column chromatography (CC_2_) using dichloromethane (DCM) and various dichloromethane/acetone mixtures (90:10, 80:20, 50:50, and 10:90, v/v), followed by acetone/methanol (90:10, v/v), yielding ten fractions (Fr.B1–Fr.B10). To eliminate chlorophylls, Fr.B3 (0.38 g), Fr.B4 (0.50 g), Fr.B5 (0.49 g), and Fr.B7 (0.41 g) were treated with activated charcoal resulting in Fr.B3′ (0.24 g), Fr.B4′ (0.40 g), Fr.B5′ (0.22 g), and Fr.B7′ (0.39 g).^11^

Fr.B3′ (0.170 g) was further fractionated on silica gel CC_3_, with the elution performed using hexane/EtOAc (95:5, 90:10, 80:20, 70:30, and 50:50, v/v), followed by pure EtOAc. This process resulted in the collection of 18 fractions (Fr.B3′.1–Fr.B3′.18). Fr.B3′.13 (0.012 g) was purified using semipreparative HPLC on a Luna C-18 (5 μm, 250 × 10 mm, 100 Å). The separation was achieved via gradient elution with CH_3_CN/H_2_O, starting at 80:20 and transitioning to 100:0 over 30 min at a flow rate of 2 mL/min, resulting in the isolation of compounds **1** (6.1 mg) and **2** (3.2 mg). Additionally, Fr.B3′ was crystallized with methanol, and the precipitate was subjected on PTLC. A total of 0.015 g was applied to a silica gel precoated plate (silica gel 60, 20 cm × 20 cm, 0.5 mm) and developed using a solvent mixture of hexane/DCM/acetone/MeOH (6:3.6:0.3:0.1). This process yielded compound **10** (4.9 mg) with an Rf of 0.19.

Fr.B5′ (0.22 g) was subjected to silica gel column chromatography (CC4) using a gradient of DCM/EtOAc (100:0, 99.5:0.5, 99:1, 98:2, 97:3, 95:5, 90:10, 80:20, 70:30, 60:40, 50:50, and 0:100, v/v) to yield 16 fractions (Fr.B5′.01–Fr.B5′.16). Fractions Fr.B5′.07 and Fr.B5′.09 were merged (0.061 g) and purified with PTLC; 0.034 g was absorbed in three PTLC plates using hexane/acetone (85:15, v/v) as eluent, and each plate was developed three times. The fractions with Rf = 0.037 yielded compound **3** (14.3 mg). Fr.B5′.10 (0.027 g) was crystallized with hexane to afford compound **3** (6.2 mg).

Fr.B7′ (0.039 g) was crystallized with EtOAc, resulting in a precipitate (0.075 g), which was then further purified via PTLC on silica gel 60 plates. A total of 0.058 g was applied to three plates, each of which was developed four times with hexane: EtOAc mixture (40:60, v/v). The fractions with Rf = 0.45 yielded sideridiol **4** (18.6 mg), as previously described.^11^ The fractions with Rf = 0.36 provided Fr.B7′.B2 (8.2 mg), while those with Rf = 0.15 yielded Fr.B7′.B3 (2.8 mg). Fr.B7′.B2 was further purified on semipreparative HPLC (Luna C-18(2), 5 μm, 250 × 10 mm, 100 Å; isocratic elution in MeOH/H_2_O; 85:15 both containing 1% CH_3_COOH, v/v; 2.5 mL/min, 40 min) yielding compound **5** (5.3 mg) and Fr.B7′.B2.3 (2.2 mg). epi-Candicandiol **8** (0.9 mg) and flavovirol **9** (0.6 mg) were isolated from Fr.B7′.B2.3 by semipreparative HPLC (Luna C-18(2), 100 Å, 250 × 10 mm, 5 μm; isocratic elution with CH_3_CN/H_2_O; 60:40, v/v; 2.5 mL/min, 45 min).

Fr.B7′.B3 (2.8 mg) was separated into sideroxol **6** (1.2 mg) and eubotriol **7** (1.1 mg) by semipreparative HPLC (Luna C-18(2), 100 Å, 250 × 10 mm, 5 μm; isocratic elution with MeOH/H_2_O; 85:15, v/v; 2.5 mL/min, 37 min).

#### 3.3.1 Ferrediol 17-Isovalerate (**1**)

Colorless oil; [α]_D_^26^ −10.20 (c 0.196 g/100 mL, MeOH); UV (MeOH) λ_max_ (log ε) 195 nm; IR *v*_max_ 3428, 2921, 2865, 1734, 1446, 1294, 1165 cm^–1^; ^1^H and ^13^C NMR spectroscopic data in Table 1; HPLC R_t_: 10.8 min, 100% CH_3_CN + 0.1% HCOOH; HRESIMS (positive) *m*/*z* 411.2870 [M + Na]^+^ (calcd for C_25_H_40_O_3_Na, 411.2870).

#### 3.3.2 Ferrediol 17-(2′-Methylbutyrate) (**2**)

Colorless oil; [α]_D_^26^ −27.97 (c 0.143 g/100 mL, MeOH); UV (MeOH) λ_max_ (log ε) 195 nm; IR *v*_max_ 3428, 2926, 2865, 1736, 1460, 1383, 1182 cm^–1^; ^1^H and ^13^C NMR spectroscopic data in Table 1; HPLC R_t_: 11.1 min, 100% CH_3_CN + 0.1% HCOOH; HRESIMS (positive) *m*/*z* 411.2865 [M + Na]^+^ (calcd for C_25_H_40_O_3_Na, 411.2870).

#### 3.3.3 Sideritriol 17-Isovalerate (**5**)

Colorless oil; [α]_D_^26^ −17.24 (c 0.116 g/100 mL, MeOH); UV (MeOH) λ_max_ (log ε) 195 nm; IR *v*_max_ 3370, 2928, 2867, 1736, 1573, 1461, 1384, 1184, 1042 cm^–1^; ^1^H and ^13^C NMR spectroscopic data in Table 1; UHPLC R_t_: 18.9 min, 85% MeOH + 0.1% CH_3_COOH; HRESIMS (positive) *m*/*z* 427.2808 [M + Na]^+^ (calcd for C_25_H_40_O_3_Na, 427.2819).

### 3.4 Cell Culture and Antiproliferative Assay

MCF-7 (estrogen receptor-positive breast cancer cells, epithelial, low metastatic), MDA-MB-231 (estrogen/progesterone receptor/human epidermal growth factor 2 amplification-negative breast cancer cells, epithelial, highly metastatic), and Hs578T (triple-negative breast cancer cells, mesenchymal, highly metastatic) were obtained from the American Type Culture Collection (ATCC) and cultured as suggested by ATCC. Malignant cells were seeded in 24-well plates at proper concentrations (30000 cells/well for MDA-MB-231 and MCF-7 and 25000 cells/well for Hs578T) and incubated in a medium supplemented with 5–10% fetal bovine serum, depending on the cell type, for 48 h, followed by overnight starvation in a serum-free medium. Compounds were diluted in DMSO, and working dilutions were prepared in a serum-free medium ranging from 5 μΜ to 200 μΜ. Malignant cells were then incubated with these working dilutions for 48 h. Subsequently, the cells were detached through trypsinization, centrifuged at 3000 rpm for 3 min, and counted using a hemocytometer without any dye. This straightforward technique is widely utilized for estimating the number of living and healthy cells within a population. This method offers significant advantages for assessing chemosensitivity, particularly as it can effectively detect cell death even in nondividing cell populations without interfering with metabolic activity. Studies have shown that compounds derived from natural products like green tea could introduce inaccuracies in the determination of the antiproliferation effect.^25^ Antiproliferation values were then calculated relative to malignant cells treated only with DMSO, which was not toxic to cells at the used concentrations. IC_50_ values [nonlinear regression analysis model of log(inhibitor) vs normalized response–variable slope], one-way ANOVA (Dunnett’s multiple comparisons test), and two-way ANOVA (Tukey’s multiple comparisons test) were calculated using GraphPad Prism 5 (Graph Pad Software).

## Supporting Information Available

^1^H, ^13^C, and 2D NMR spectra of compounds **1**–**7** and **10**; APT NMR spectra of compounds **1**–**5** and **10**; ^1^H and ^13^C NMR spectra of compounds **8**–**9**; ESI-high-resolution mass spectra of compounds **1**–**5**; IR spectra of compounds **1**, **2**, and **5**; and an overview of isolation procedures (PDF)
