# Supplementary material for: New Esters of Ferrediol and Sideritriol in Sideritis clandestina subsp. peloponnesiaca: Characterization and Antiproliferative Activity
Source: ACS Omega. 2024 Oct 4;9(41):42253–60. doi: 10.1021/acsomega.4c04628 (PMC11483387; doi:10.1021/acsomega.4c04628)
Supplement: Supplementary file 1 — ao4c04628_si_001.pdf [file ao4c04628_si_001.pdf]

SUPPORTING INFORMATION

New esters of ferrediol and sideritriol in *Sideritis clandestina* subsp. *peloponnesiaca*: characterization and anti-proliferative activity

Virginia D. Dimaki [1], Efstathia Stamopoulou [1], Dimitra Manou [2], Achilleas D. Theocharis [2], Manolis Fousteris [1], Fotini N. Lamari [1]**

[1] Department of Pharmacy, [2] Department of Chemistry, University of Patras, 26504 Patras, Greece

TABLE OF CONTENTS		
Table S1	NMR Spectroscopy Data of siderol (**3**) (700 MHz in $CDCl_3$), sideridiol (**4**) (700 MHz in CD_3OD) and sideroxol (**6**) (600 MHz in CD_3OD) (δ in ppm, J in Hz)	S5
Table S2	NMR Spectroscopy Data of eubotriol (**7**) and *epi*-candicandiol (**8**) (600 MHz in CD_3OD) (δ in ppm, J in Hz)	S6
Table S3	NMR Spectroscopy Data (700 MHz in $CDCl_3$) of β-sitosterol (**10**) (δ in ppm, J in Hz)	S7
Figure S1	HRESIMS data of ferrediol isovalerate (**1**)	S9
Figure S2	IR spectrum of ferrediol isovalerate (**1**) in acetonitrile	S10
Figure S3	^{1}H-NMR spectrum of ferrediol isovalerate (**1**) in $CDCl_3$ (700 MHz)	S11
Figure S4	^{13}C-NMR spectrum of ferrediol isovalerate (**1**) in $CDCl_3$ (176 MHz)	S12
Figure S5	APT NMR spectrum of ferrediol isovalerate (**1**) in $CDCl_3$ (176 MHz)	S13
Figure S6	HSQC NMR spectrum of ferrediol isovalerate (**1**) in $CDCl_3$ (700 MHz)	S14
Figure S7	HMBC NMR spectrum of ferrediol isovalerate (**1**) in $CDCl_3$ (700 MHz)	S15
Figure S8	COSY NMR spectrum of ferrediol isovalerate (**1**) in $CDCl_3$ (700 MHz)	S16
Figure S9	ROESY NMR spectrum of ferrediol isovalerate (**1**) in CDCl3 (700 MHz)	S17
Figure S10	HRESIMS data of ferrediol 2-methylbutyrate (**2**)	S18
Figure S11	IR spectrum of ferrediol 2-methylbutyrate (**2**) in acetonitrile	S19
Figure S12	^{1}H-NMR spectrum of ferrediol 2-methylbutyrate *(***2**) in $CDCl_3$ (700 MHz)	S20
Figure S13	^{13}C-NMR spectrum of ferrediol 2-methylbutyrate (**2**) in $CDCl_3$ (176 MHz).	S21
Figure S14	APT NMR spectrum of ferrediol 2-methylbutyrate (**2**) in $CDCl_3$, (176 MHz)	S22
Figure S15	HSQC NMR spectrum of ferrediol 2-methylbutyrate (**2**) in $CDCl_3$ (700 MHz)	S23
Figure S16	HMBC NMR spectrum of ferrediol 2-methylbutyrate (**2**) in $CDCl_3$ (700 MHz)	S24
Figure S17	COSY NMR spectrum of ferrediol 2-methylbutyrate (**2**) in $CDCl_3$ (700 MHz).	S25
Figure S18	ROESY NMR spectrum of ferrediol 2-methylbutyrate (**2**) in $CDCl_3$ (700 MHz)	S26
Figure S19	HRESIMS data of sideritriol isovalerate (**5**)	S27
Figure S20	IR spectrum of sideritriol isovalerate (**5**) in methanol	S28

Figure S21	^{1}H-NMR spectrum of sideritriol isovalerate (**5**) in CD_3OD (600 MHz)	S29
Figure S22	^{13}C-NMR spectrum of sideritriol isovalerate (**5**) in CD_3OD (150 MHz)	S30
Figure S23	APT NMR spectrum of sideritriol isovalerate (**5**) in CD_3OD (150 MHz)	S31
Figure S24	HSQC NMR spectrum of sideritriol isovalerate (**5**) in CD_3OD (600 MHz)	S32
Figure S25	HMBC NMR spectrum of sideritriol isovalerate (**5**) in CD_3OD (600 MHz).	S33
Figure S26	COSY NMR spectrum of sideritriol isovalerate (**5**) in CD_3OD (600 MHz)	S34
Figure S27	ROESY NMR spectrum of sideritriol isovalerate (**5**) in CD_3OD (600 MHz)	S35
Figure S28	HRESIMS data of siderol (**3**)	S36
Figure S29	^{1}H-NMR spectrum of siderol (**3**) in $CDCl_3$ (700 MHz)	S37
Figure S30	^{13}C-NMR spectrum of siderol (**3**) in $CDCl_3$ (176 MHz)	S38
Figure S31	APT NMR spectrum of siderol (**3**) in $CDCl_3$ (176 MHz)	S39
Figure S32	HSQC spectrum of siderol (**3)** in $CDCl_3$ (700 MHz)	S40
Figure S33	HMBC NMR spectrum of siderol (**3**) in $CDCl_3$ (700 MHz)	S41
Figure S34	COSY NMR spectrum of siderol (**3**) in $CDCl_3$ (700 MHz)	S42
Figure S35	ROESY NMR spectrum of siderol (**3**) in $CDCl_3$ (700 MHz)	S43
Figure S36	HRESIMS data of sideridiol (**4**)	S44
Figure S37	^{1}H-NMR spectrum of sideridiol (**4**) in CD_3OD (700 MHz)	S45
Figure S38	^{13}C-NMR spectrum of sideridiol (**4**) in CD_3OD (176 MHz)	S46
Figure S39	APT NMR spectrum of sideridiol (**4**) in CD_3OD (176 MHz)	S47
Figure S40	HSQC NMR spectrum of sideridiol (**4**) in CD_3OD (700 MHz).	S48
Figure S42	HMBC NMR spectrum of sideridiol (**4**) in CD_3OD (700 MHz)	S49
Figure S42	COSY NMR spectrum of sideridiol (**4**) in CD_3OD (700 MHz)	S50
Figure S43	ROESY NMR spectrum of sideridiol (**4**) in CD_3OD (700 MHz)	S51
Figure S44	^{1}H-NMR spectrum of sideroxol (**6**) in CD_3OD (600 MHz)	S52
Figure S45	^{13}C-NMR spectrum of sideroxol (**6**) in CD_3OD (150 MHz)	S53
Figure S46	DEPT135-NMR spectrum of sideroxol (**6**) in CD_3OD (150 MHz)	S54
Figure S47	HSQC NMR spectrum of sideroxol (**6**) in CD_3OD (600 MHz)	S55
Figure S48	HMBC NMR spectrum of sideroxol (**6**) in CD_3OD (600 MHz).	S56
Figure S49	COSY NMR spectrum of sideroxol (**6**) in CD_3OD (600 MHz)	S57
Figure S50	ROESY NMR spectrum of sideroxol (**6**) in CD_3OD (600 MHz)	S58
Figure S51	^{1}H-NMR spectrum of eubotriol (**7**) in CD3OD (600 MHz)	S59
Figure S52	^{13}C-NMR spectrum of eubotriol (**7**) in CD_3OD (150 MHz)	S60
Figure S53	HSQC NMR spectrum of eubotriol (**7**) in CD_3OD (600 MHz)	S61
Figure S54	HMBC NMR spectrum of eubotriol (**7**) in CD_3OD (600 MHz)	S62
Figure S55	COSY NMR spectrum of eubotriol (**7**) in CD_3OD (600 MHz)	S63
Figure S56	ROESY NMR spectrum of eubotriol (**7**) in CD_3OD (600 MHz)	S64
Figure S57	^{1}H-NMR spectrum of e*pi*-candicandiol (**8**) CD_3OD (600 MHz)	S65
Figure S58	^{13}C-NMR spectrum of e*pi*-candicandiol (**8**) in CD_3OD (150 MHz)	S66
Figure S59	^{1}H-NMR spectrum of flavovirol (**9**) in CD_3OD (600 MHz)	S67
Figure S60	^{13}C-NMR spectrum of flavovirol (**9**) in CD_3OD (150 MHz)	S68
Figure S61	^{1}H-NMR spectrum of β-sitosterol (**10**) in $CDCl_3$ (700 MHz)	S69

Figure S62	^{13}C-NMR spectrum of β-sitosterol (**10**) in $CDCl_3$ (176 MHz)	S70
Figure S63	APT NMR spectrum of β-sitosterol (**10**) in $CDCl_3$, (176 MHz)	S71
Figure S64	HSQC NMR spectrum of β-sitosterol (**10**) in $CDCl_3$ (700 MHz)	72
Figure S65	HMBC NMR spectrum of β-sitosterol (**10**) in $CDCl_3$ (700 MHz)	S73
Figure S66	COSY NMR spectrum of β-sitosterol (**10**) in $CDCl_3$ (700 MHz)	S74
Figure S67	ROESY NMR spectrum of β-sitosterol (**10**) in $CDCl_3$ (700 MHz)	S75
Scheme S1	Overview of isolation procedures	S76

Table S1. NMR Spectroscopy Data of siderol (**3**) (700 MHz in $CDCl_3$), sideridiol (**4**) (700 MHz in CD_3OD) and sideroxol (**6**) (600 MHz in CD_3OD) (δ in ppm, J in Hz).

	Siderol **3**		Sideridiol **4**		Sideroxol **6**	
position	δc, type	δH (*J*in Hz)	δc, type	δH (*J* in Hz)	δc, type	δH (*J*in Hz)
1	42.00, CH_2	1.80, d (12.6)	41.19, CH_2	1.81, dd (2.6, 12.6)	41.13, CH_2	1.84, d, (13.2)
		0.84, td (3.6, 13.0)		0.83, td (3.5, 12.6)		0.87, td (3.4, 12.8)
2	18.30, CH_2	1.54-1.44, m	19.07, CH_2	1.58-1.41, m	18.94, CH_2	1.72-1.56, m, 1.53-1.44, m
3	35.21, CH_2	1.54-1.44, m	36.55, CH_2	1.43, dd (4.2, 13.3)	36.55, CH_2	1.53-1.44, m
		1.25-1.22, m		1.31-1.29, m		1.32, s
4	36.99, C	-	38.25, C	-	38.23, C	
5	44.56, CH	1.66-1.64, m	40.99, CH	1.70-1.68, m	40.90, CH	1.76 dd (1.7, 12.7)
6	23.51, CH_2	1.75, ddd (1.7, 3.7, 14.4)	27.60, CH_2	1.58-1.41, m	27.61, CH_2	1.72-1.56, m, 1.53-1.44, m
		1.54-1,44, m				
7	78.26, CH	4.69, dd (2.2, 3.6)	76.29, CH	3.53, t (2.8)	72.83, CH	3.66, t (2.8)
8	51.87, C	-	54.56, C	-	49.00, C*,	
9	44.87, CH	1.36-1.35, m	45.21, CH	1.36-1.34, m	46.91, CH	1.72-1.56, m
10	39.10, C	-	40.53, C	-	40.15, C	-
11	17.93, CH_2	1.54-1,44, m	19.47, CH_2	1.58-1.41, m	18.69, CH_2	1.72-1.56, m
12	24.79, CH_2	1.54-1,44, m	25.96, CH_2	1.58-1.41, m	28.49, CH_2	1.72-1.56, m
13	39.80, CH	2.36, m	46.07, CH	2.39-2.32, m	40.63, CH	2.10, m
14	39.86, CH_2	1.96, d (10.0)	43.49, CH_2	1.98, d (10.0)	32.30, CH_2	1.53-1.44, m
		1.54-1.44, m		1.36-1.34, m		1.02, dd (4.8, 11.3)
15	129.80, CH	5.26, s	131.96, CH	5.52, s	65.80, CH	3.11, s
16	143.81, C	-	144.10, C	-	63.37, C	-
17	15.38, CH_3	1.70, d (1.6)	15.54, CH_3	1.71, d (1.5)	14.69, CH_3	1.42, s
18	71.41, CH_2	3.32, d (10.8)	72.36, CH_2	3.30, d (11.0)	72.34, CH_2	3.31, m*
		3.00, d (10.8)		3.06, d (11.2)		3.07, d (11.2)
19	17.36, CH_3	0.71, s	17.65, CH_3	0.76, s	17.68, CH_3	1.07, s
20	17.76, CH_3	1.07, s	18.56, CH_3	1.10, s	18.27, CH_3	0.76, s
C=O	170.75, C	-	-	-	-	-
$\underline{CH_3}$-OAc	21.46, CH_3	2,07, s	-	-	-	-

*Overlapping with solvent

Table S2. NMR Spectroscopy Data of eubotriol (**7**) and epi-candicandiol (**8**) (600 MHz in CD_3OD) (δ in ppm, J in Hz).

	Eubotriol **7**		*epi*-Candicandiol **8**	
position	δc, type	δH (j in Hz)	δc, type	δH (j in Hz)
1	41.00, CH_2	1.84, d (12.5)	41.08, CH_2	1.83, d (12.8)
		0.84, br, s		0.84, td (4.0, 13.0)
2	19.02, CH_2[a]	1.71-1.61, m	36.50, CH_2	1.49-1.41, m
		1.49-1.37, m		1.32-1.30, m
3	36.47, CH_2	1.31-1.30, m	34.68, CH_2	1.77-1.57, m
				1.49-1.41, m
4	38.24, C	-	38.21, C[b]	
5	41.18, CH	1.81-1.77, m	41.31, CH	1.77-1.57, m
6	27.38, CH_2	1.71-1.61, m	28.39, CH_2	1.77-1.57, m
7	74.32, CH	3.85, t (2.9)	78.19, CH	3.52, t (2.6)
8	52.44, C	-	49.00, C*	-
9	50.67, CH	1.49-1.37, m	51.76, CH	1.49-1.41, m
10	40.26, C	-	40.16, C[b]	-
11	18.64, CH_2[a]	1.71-1.61, m	19.06, CH_2[c]	1.77-1.57, m
		1.49-1.37, m		
12	34.24, CH_2	1.71-1.61, m	18.82, CH_2[c]	1.49-1.41, m
		1.49-1.37, m		
13	44.20, CH	2.76, s	45.21, CH	2.64, s
14	36.09, CH_2	1.81-1.77, m	39.78, CH_2	1.92, dd (1.7, 11.3)
		1.49-1.37, m		1.14, dd (5.0, 11.3)
15	82.34, CH	4.07, s	46.50, CH	2.25-2.23, m
16	159.25, C	-	156.78, C	
17	108.99, =C$\underline{H}_2$	5.19 s & 5.07 s	103.67, =C$\underline{H}_2$	4.79 s & 4.75 s
18	72.31, CH_2	3.30, m*	72.42, CH_2	3.30, m*
		3.07, d (11.2)		3.07, d (11.2)
19	17.70, CH_3	0.78, s	17.75, CH_3	0.78, s
20	18.34, CH_3	1.09, s	18.49, CH_3	1.10, s

*Overlapping with solvent. [a]These values can be interchanged. [b]These values can be interchanged. [c]These values can be interchanged.

Table S3. NMR Spectroscopy Data (700 MHz in $CDCl_3$) of β-sitosterol (**10**) (*δ* in ppm, *J* in Hz)

	β-Sitosterol 1**0**	
position	δc, type	δH (*J* in Hz)
1	37.27, CH_2	1.86-1.84, m
		1.16-1.06, m
2	31.68, CH_2	1.86-1.84, m
		1.55-146, m
3	71.83, CH	3.54-3.50, m
4	42.32, CH_2	2.32-2.30, ddd (2.3, 5.0, 13.0)<br>2.27-2.23, m
5	140.77, C	-
6	121.74, CH	5.35-5.34, m
7	31.93, CH_2	2.04-1.97
		1.86-1.84, m
8	31.92, CH	1.55-1.46, m
9	50.15, CH	0.97-0.93, m
10	36.52, C	-
11	21.10, CH_2	1.55-146, m
12	39.79, CH_2	2.04-1.97, m
		1.20-1.16, m
13	42.34, C	-
14	56.78, CH	1.04-1.02, m
15	24.32, CH_2	1.61-1.58, m
		1.16-1.06, m
16	28.26, CH_2	1.86-1.84, m
		1.38-1.22, m
17	56.07, CH	1.16-1.06, m
18	11.88, CH_3	0.7, s
19	19.41, CH_3	1.08, s
20	36.16, CH	1.38-1.22, m
21	18.79, CH_3	0.94, d (6.6)
22	33.96, CH_2	1.38-1.22, m
		1.04-1.02, m
23	26.09, CH_2	1.20-1.16, m

24	45.85, CH	0.97-0.93, m
25	29.17, CH	1.69-1.86, m
26	19.83, CH_3	0.87-0.85, m
27	19.05, CH_3	0.83-0.82, m
28	23.08, CH_2	1.38-1.22, m
29	12.00, CH_3	0.87-0.85, m

m/z	Theo. Mass	Delta (ppm)	RDB equiv.	Composition
411.2870	411.2870	0.13	5.5	C25 H40 O3 Na

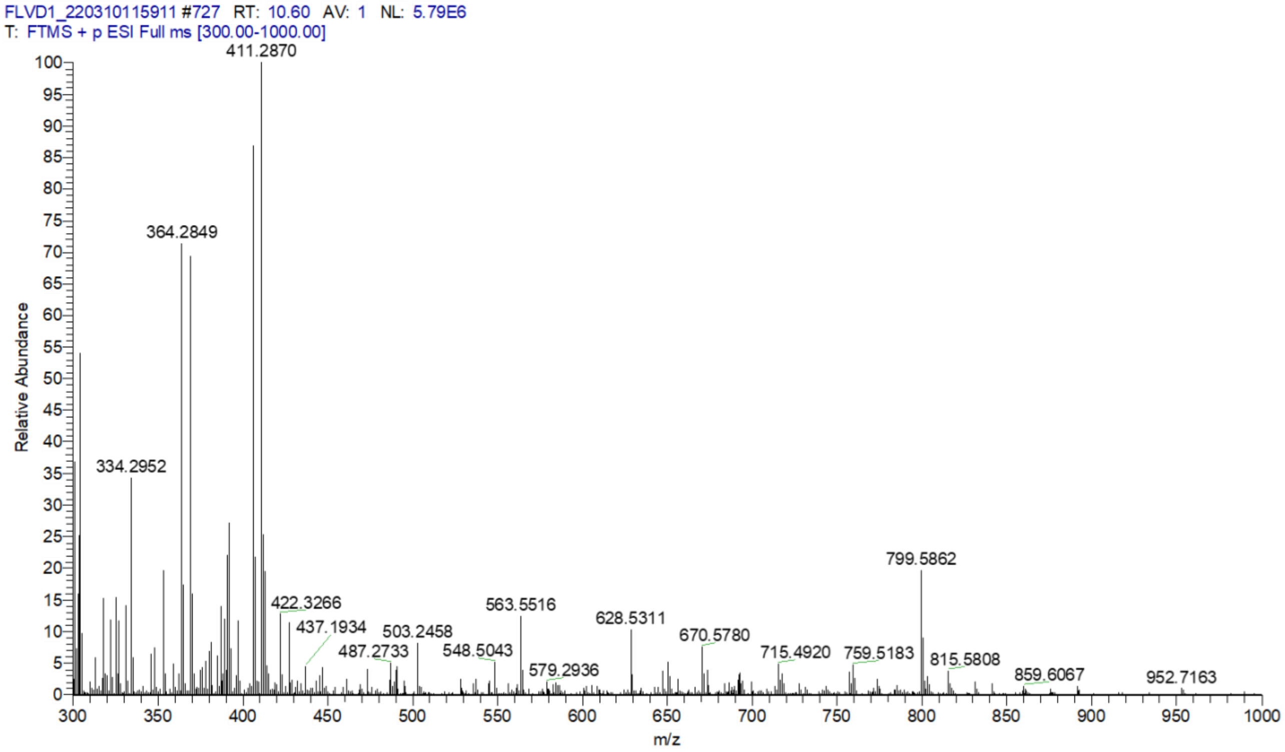

Figure S1. HRESIMS data of ferrediol isovalerate (**1**).

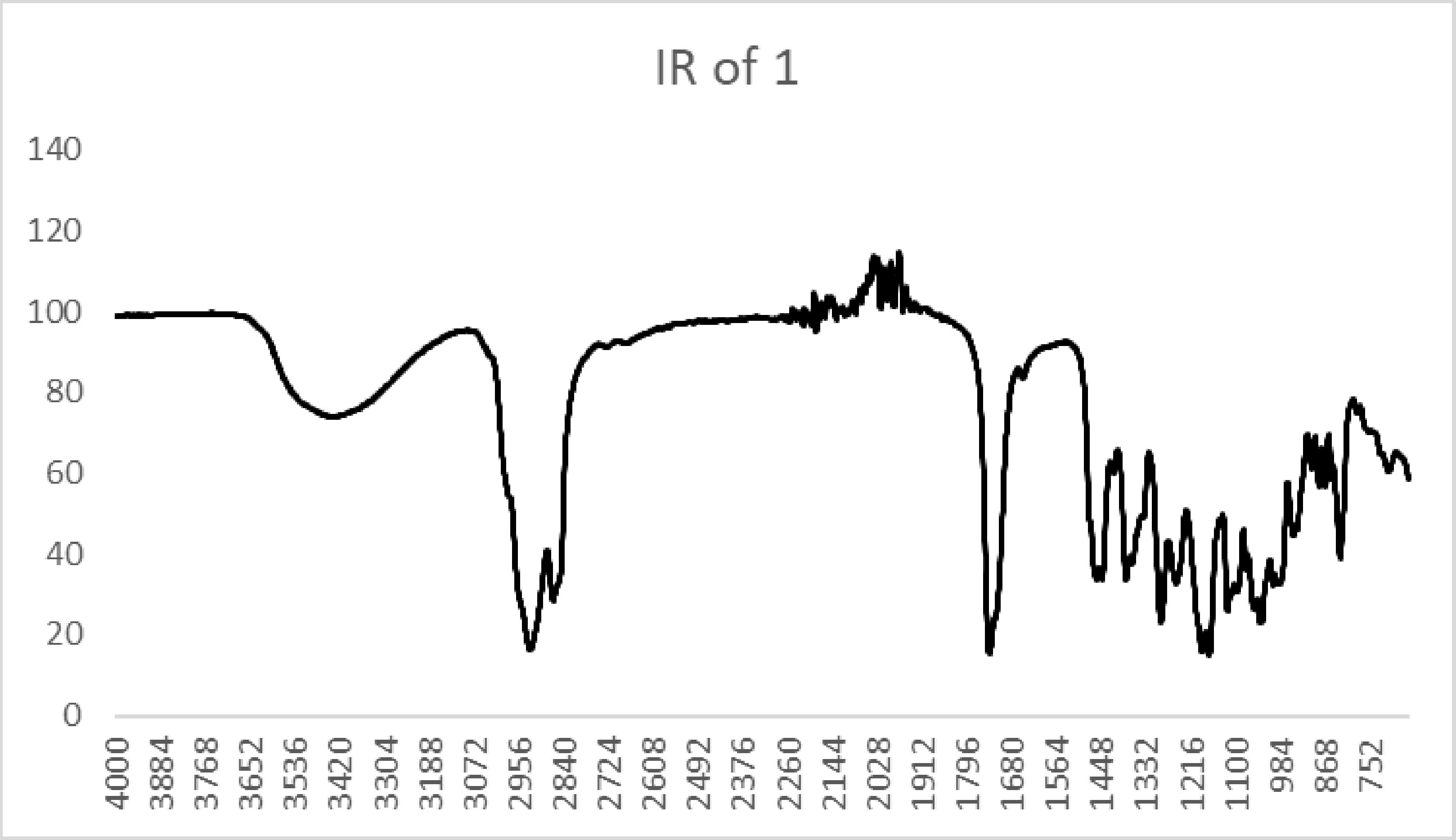

Figure S2. IR spectrum of ferrediol isovalerate (**1**) in acetonitrile.

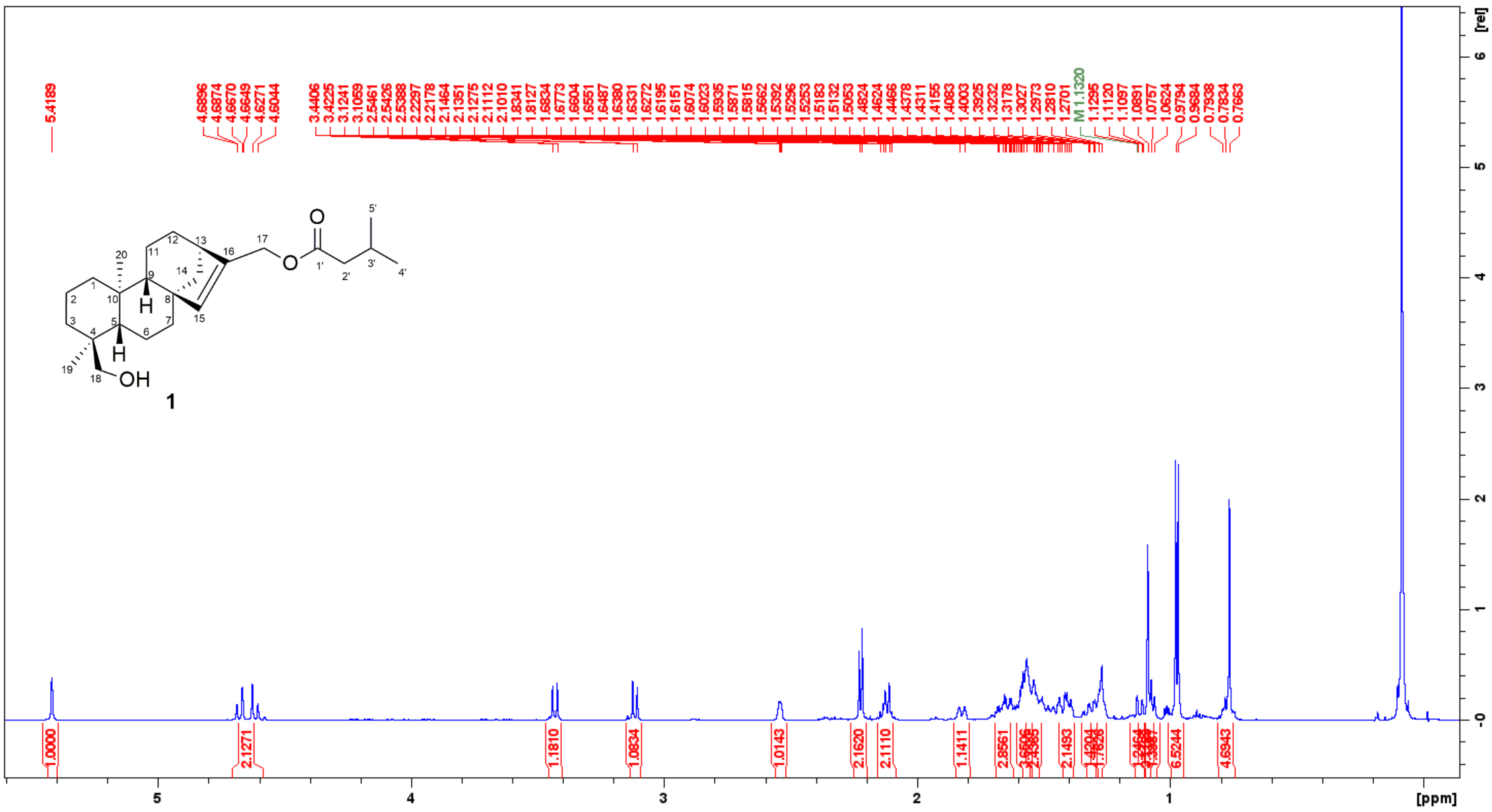

Figure S3. ^{1}H-NMR spectrum of ferrediol isovalerate (**1**) in $CDCl_3$ (700 MHz).

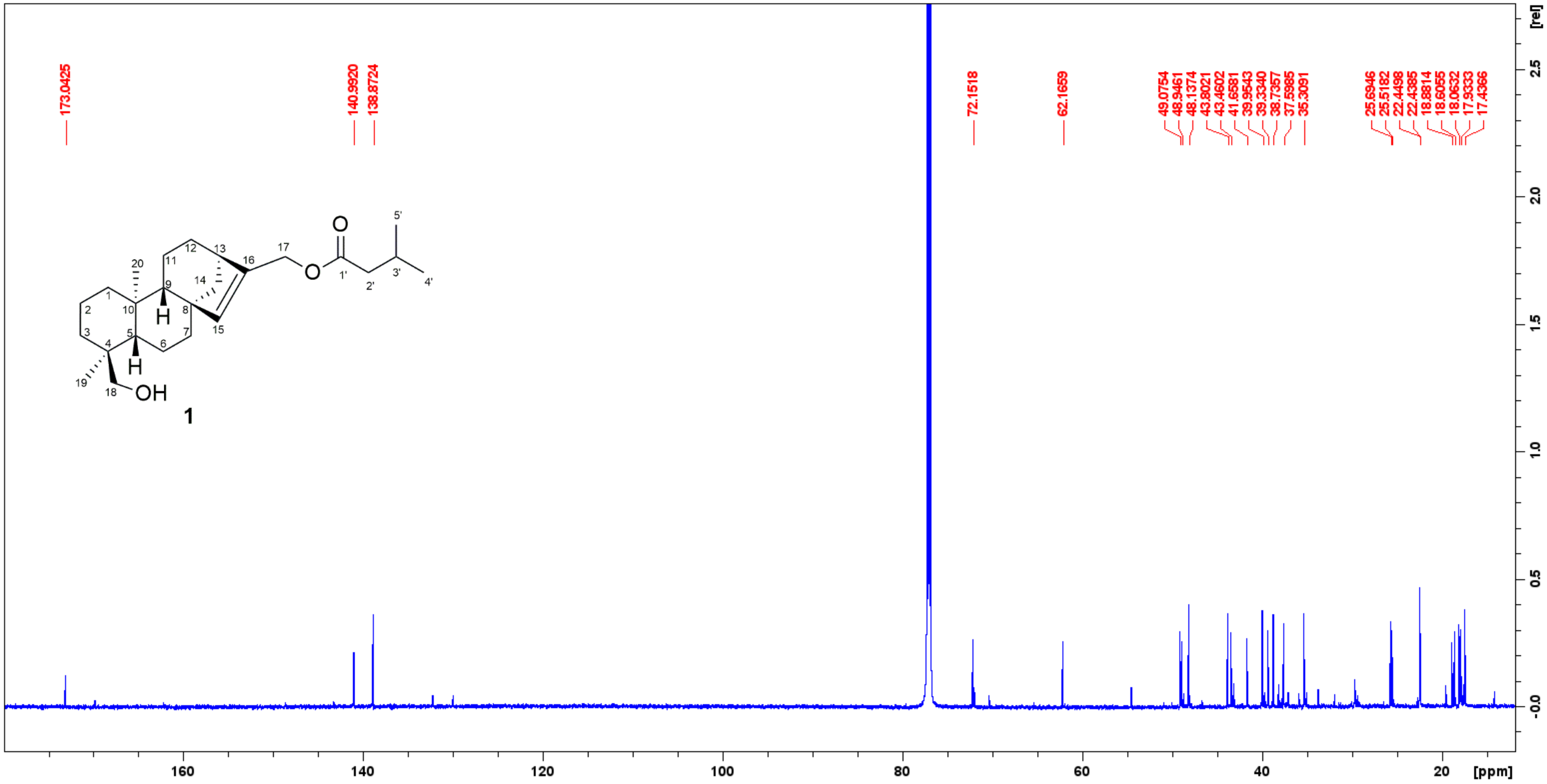

Figure S4. ^{13}C-NMR spectrum of ferrediol isovalerate (**1**) in $CDCl_3$ (176 MHz)

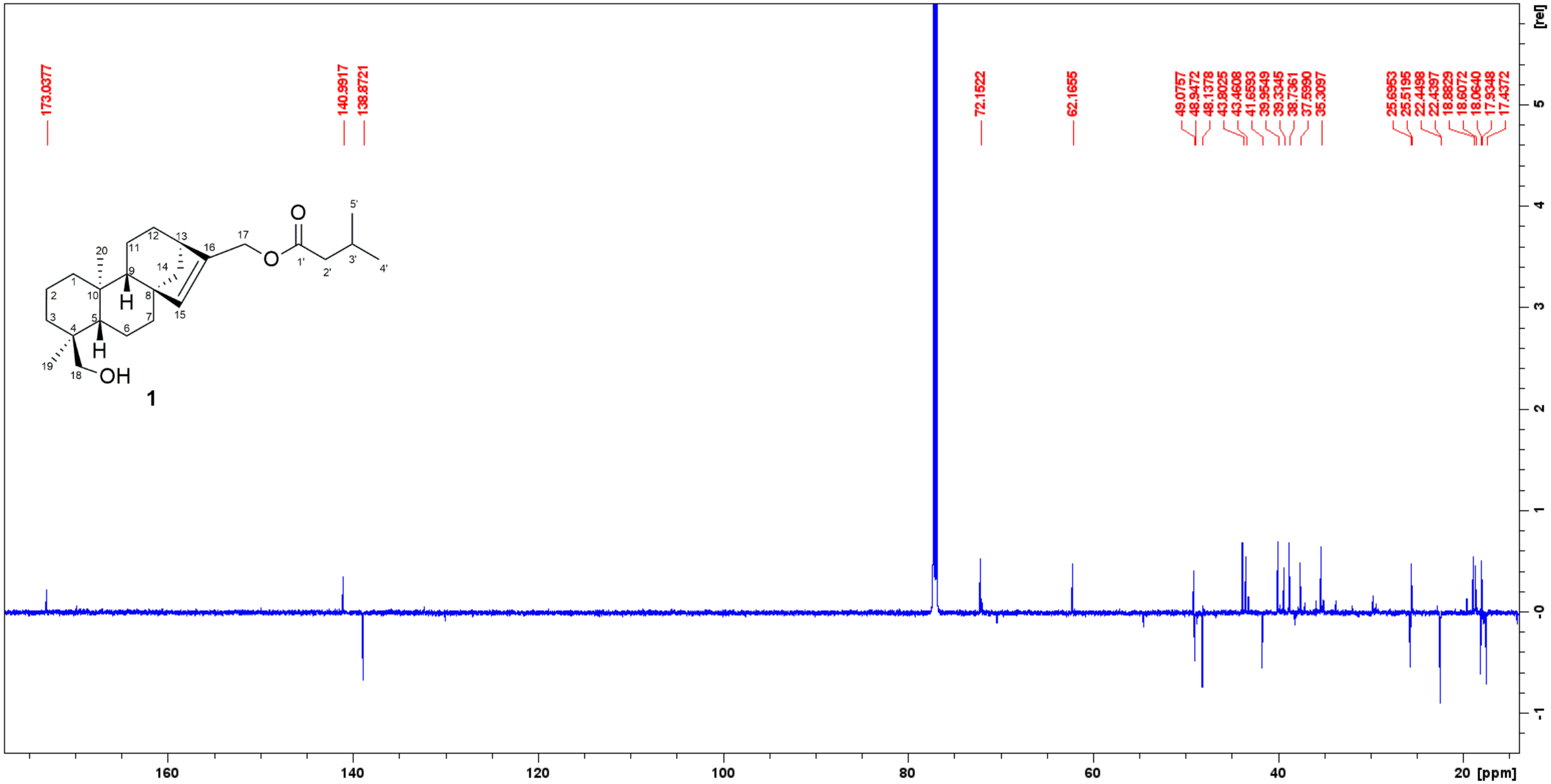

Figure S5. APT NMR spectrum of ferrediol isovalerate (**1**) in $CDCl_3$ (176 MHz).

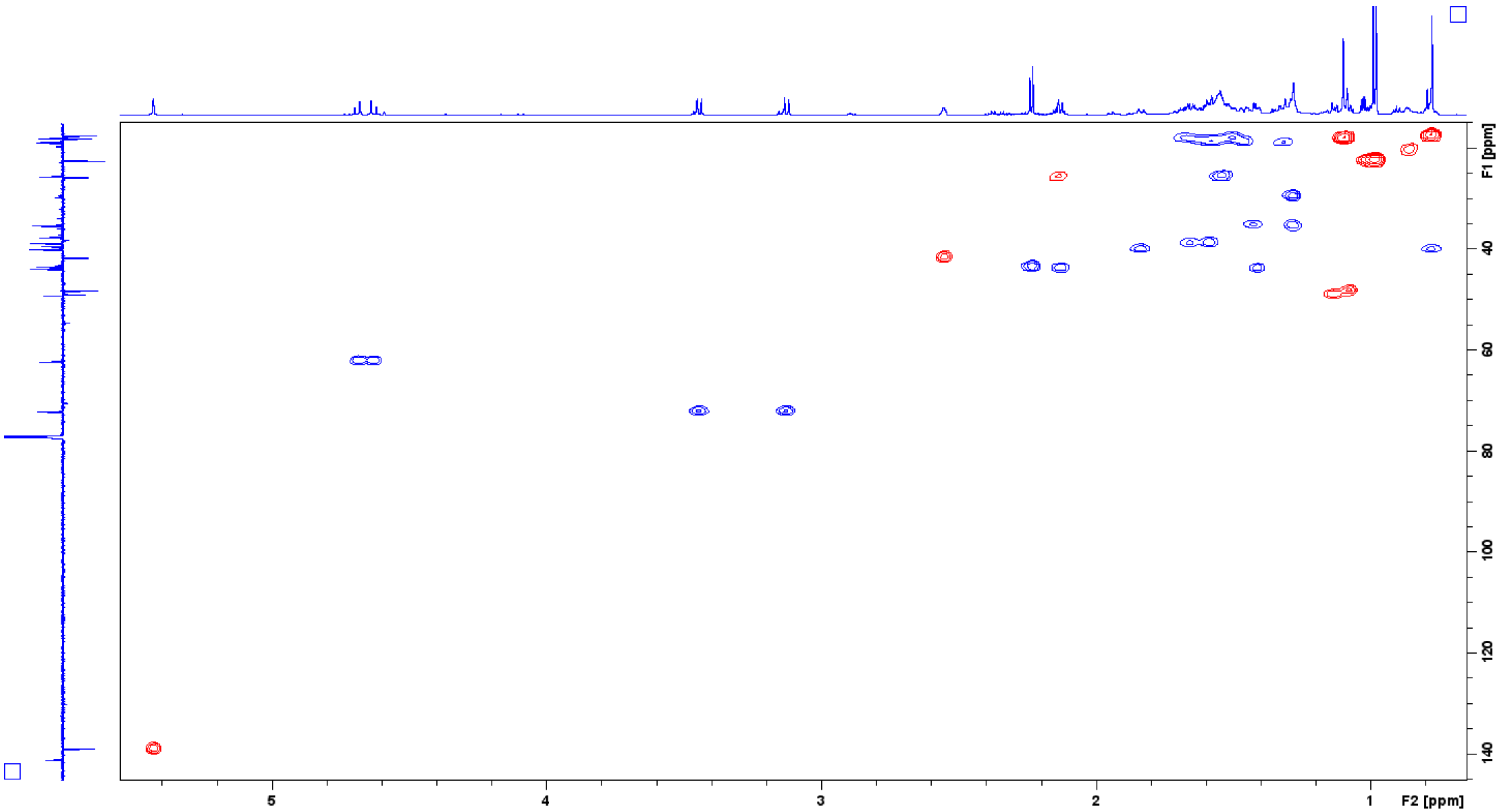

Figure S6. HSQC NMR spectrum of ferrediol isovalerate (**1**) in $CDCl_3$ (700 MHz).

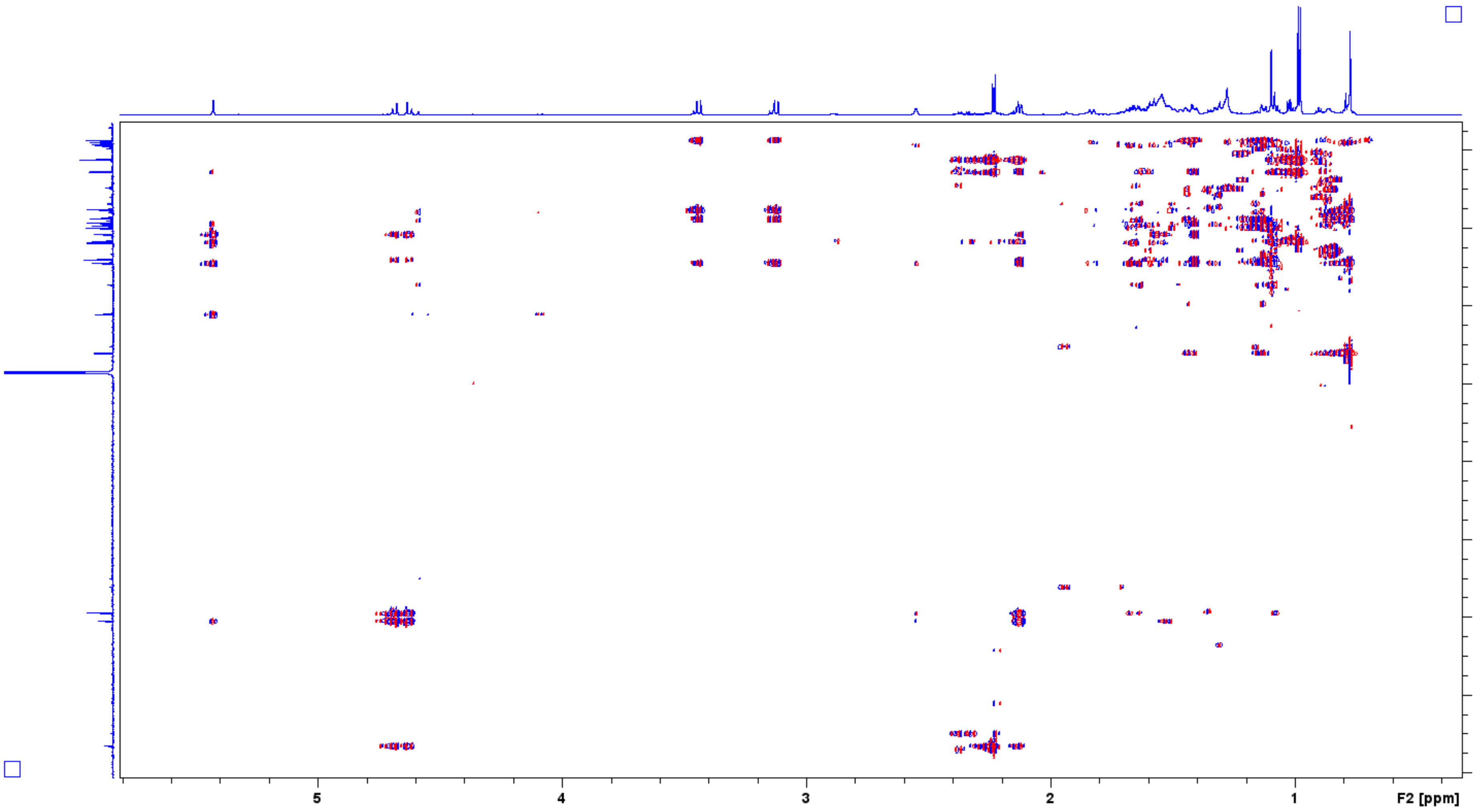

Figure S7. HMBC NMR spectrum of ferrediol isovalerate (**1**) in $CDCl_3$ (700 MHz).

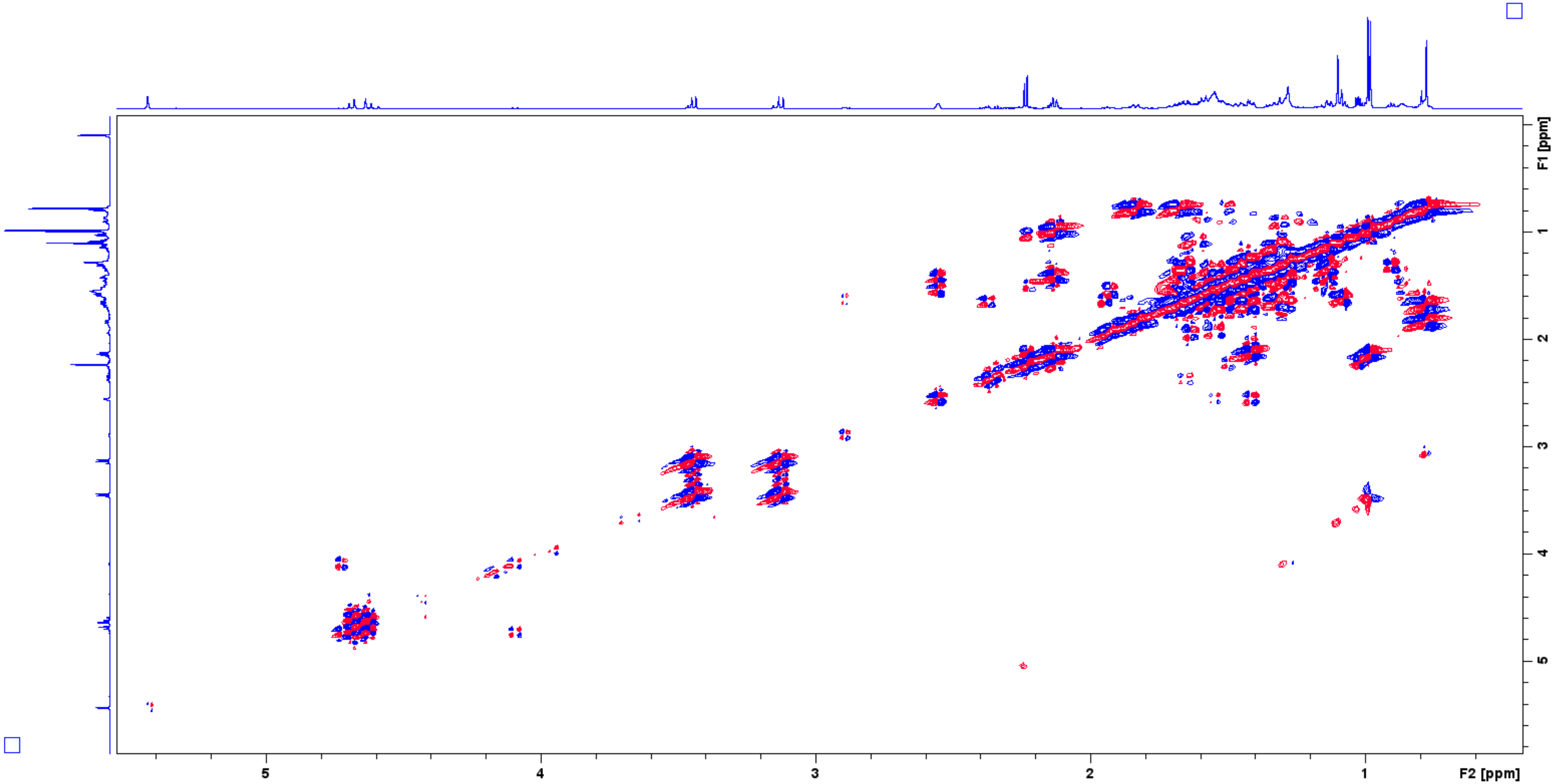

Figure S8. COSY NMR spectrum of ferrediol isovalerate (**1**) in $CDCl_3$ (700 MHz).

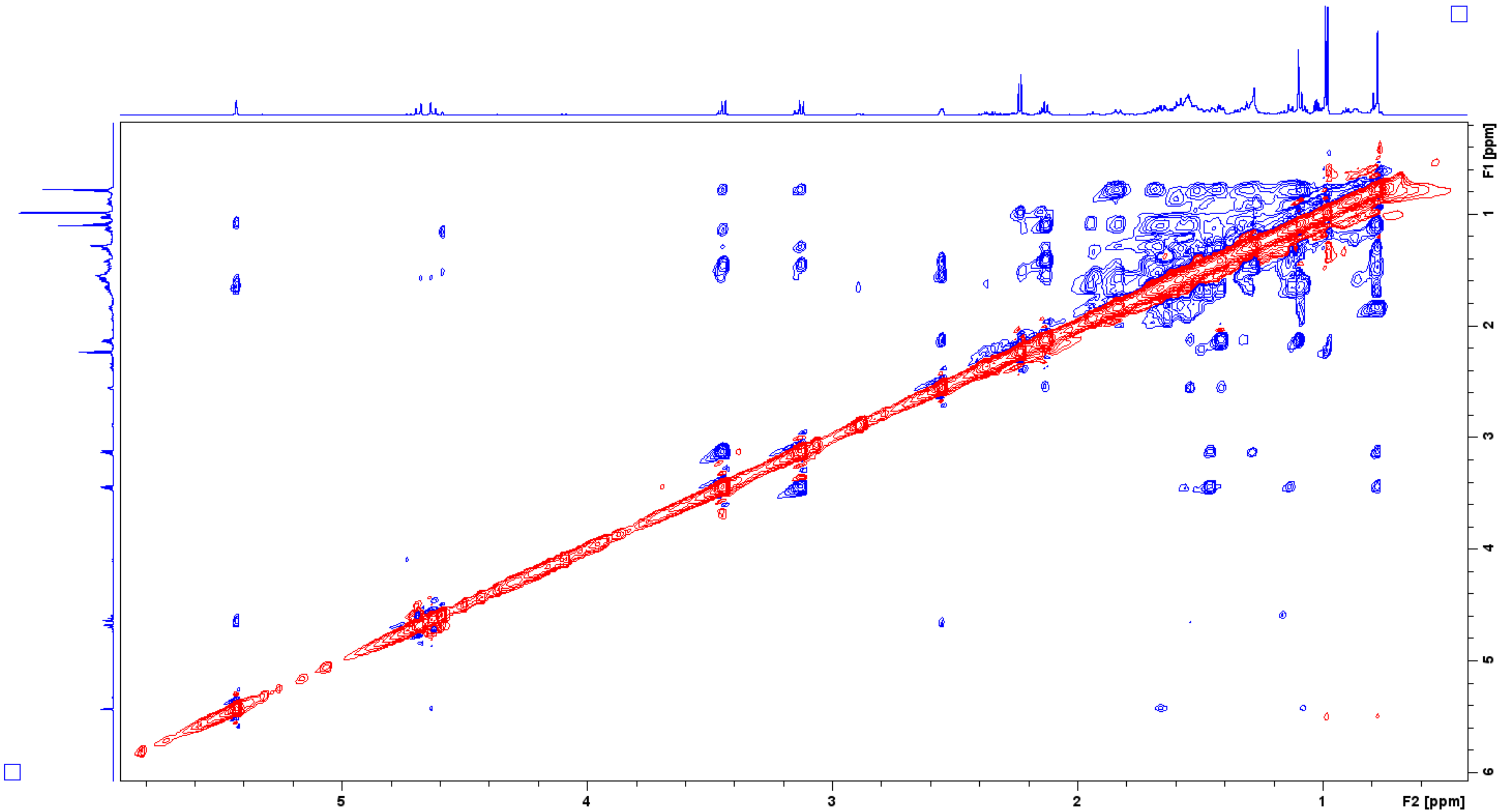

Figure S9. ROESY NMR spectrum of ferrediol isovalerate (**1**) in $CDCl_3$ (700 MHz).

m/z	Theo. Mass	Delta (ppm)	RDB equiv.	Composition
411.2865	411.2870	-1.21	5.5	C25 H40 O3 Na

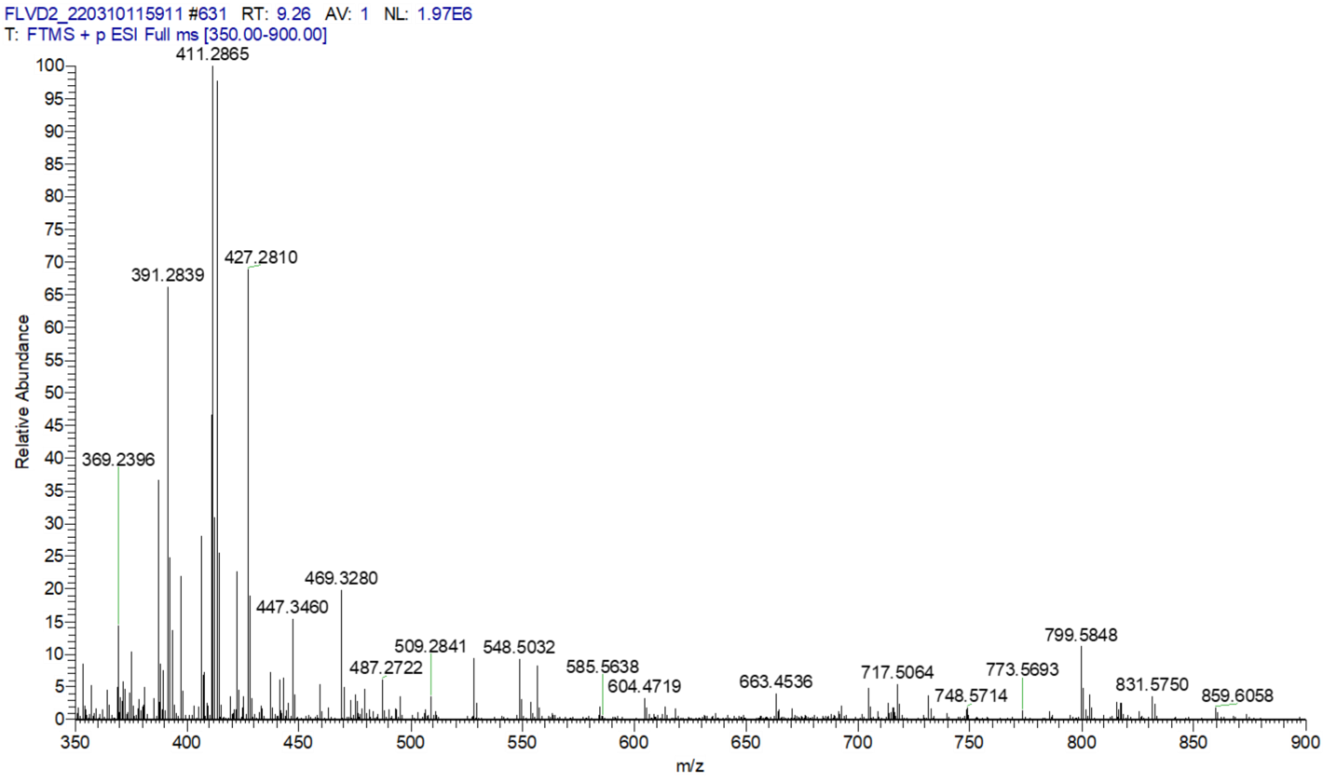

Figure S10. HRESIMS data of ferrediol 2-methylbutyrate (**2**).

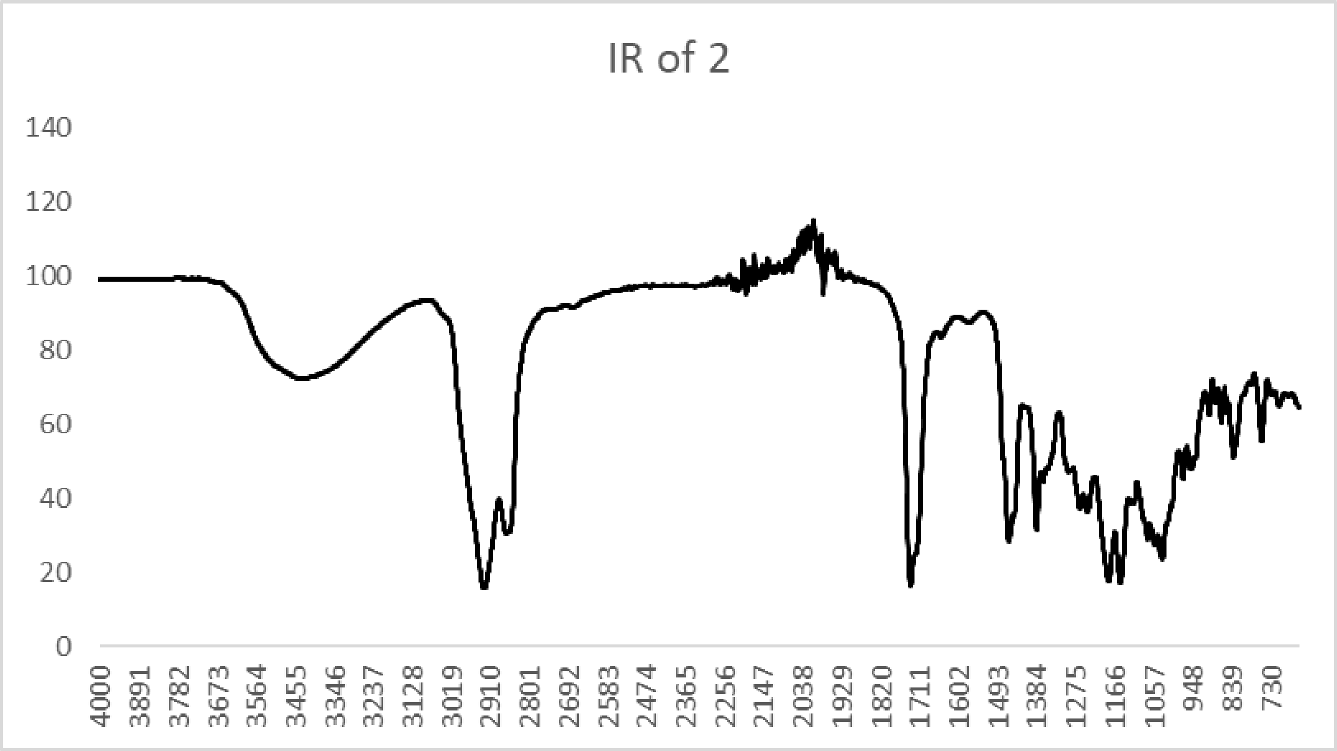

Figure S11: IR spectrum of ferrediol 2-methylbutyrate (**2**) in acetonitrile.

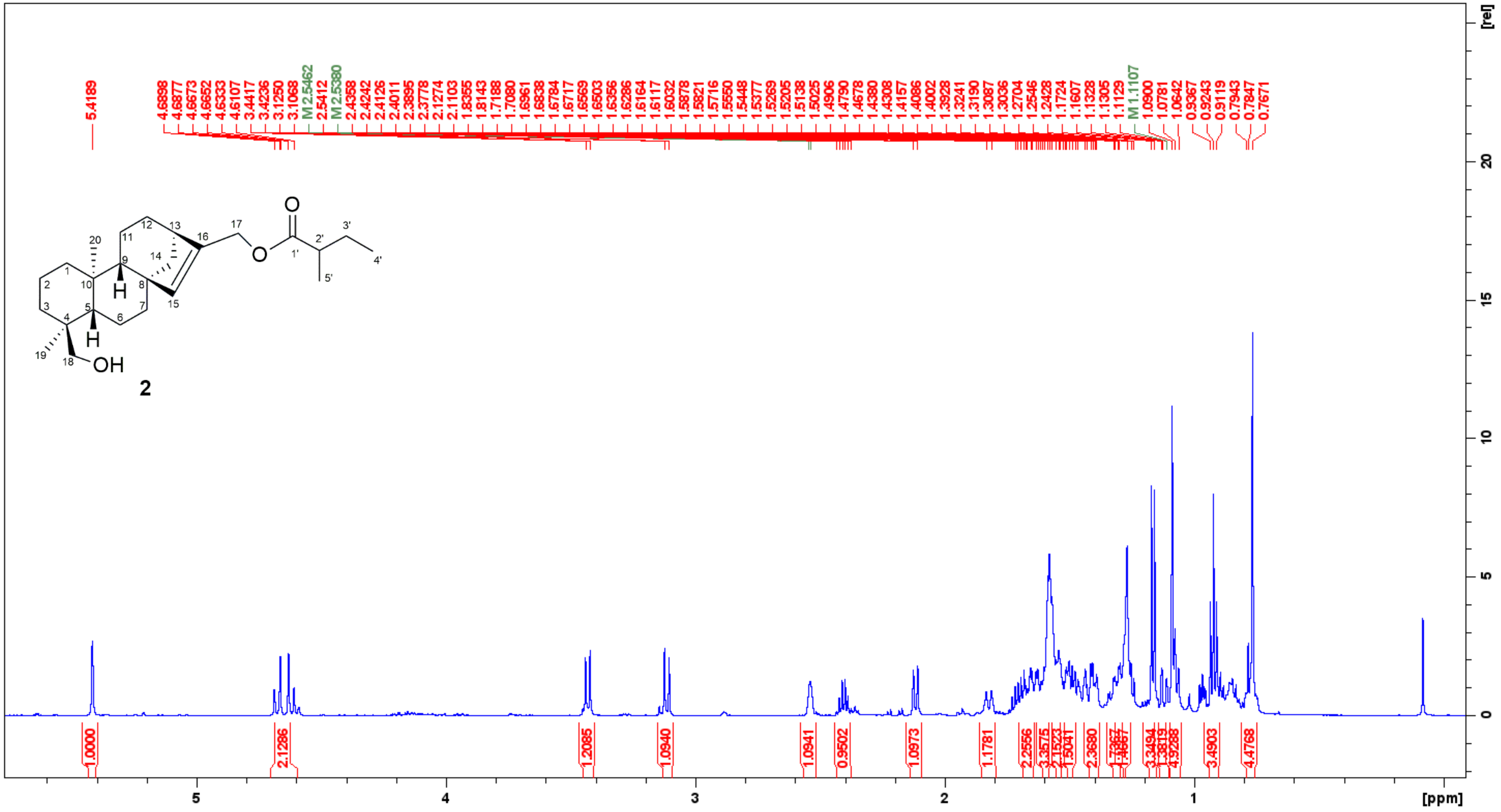

Figure S12. ^{1}H-NMR spectrum of ferrediol 2-methylbutyrate *(**2**)* in $CDCl_3$ (700 MHz).

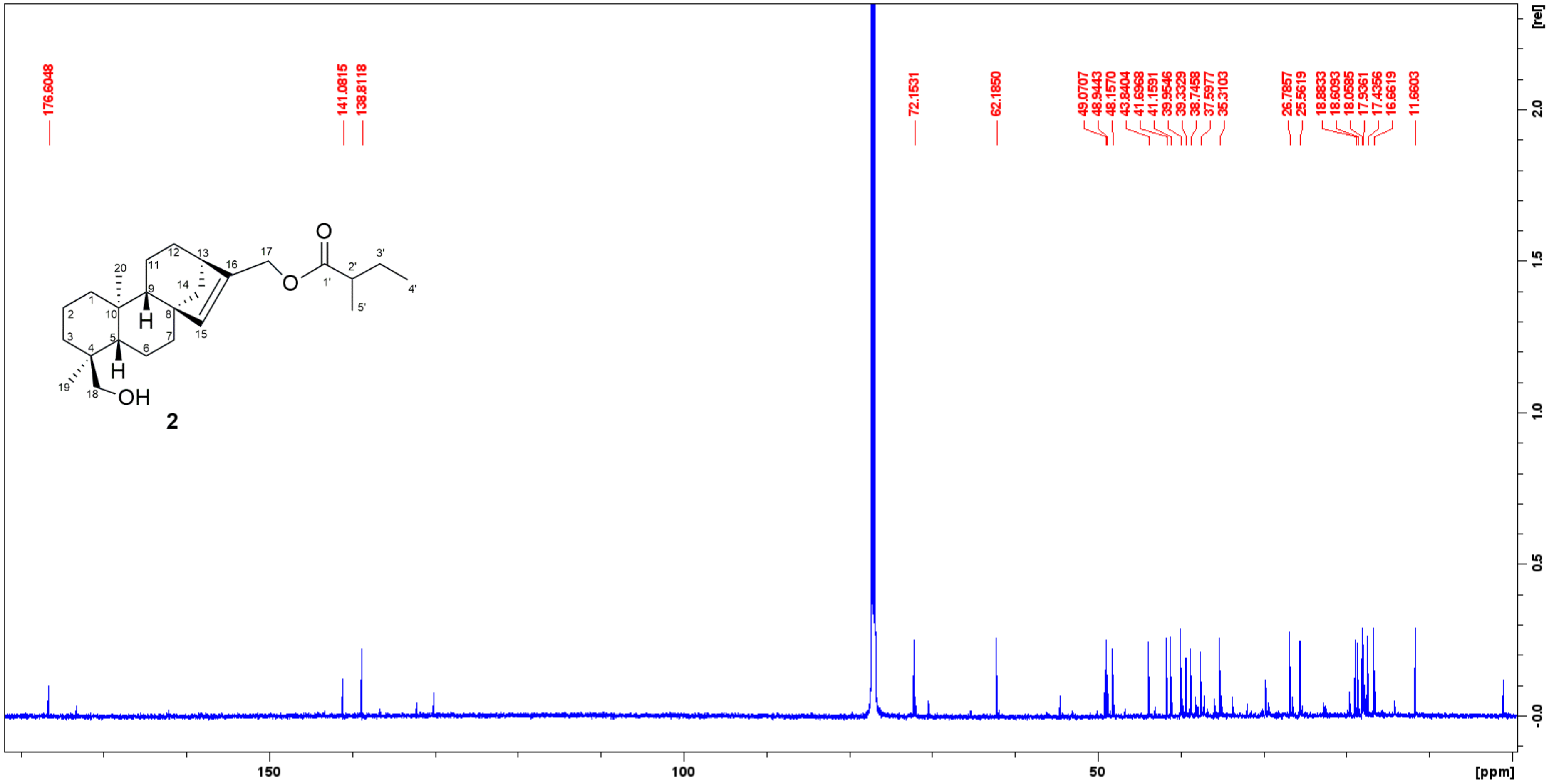

Figure S13. ^{13}C-NMR spectrum of ferrediol 2-methylbutyrate *(***2***)* in $CDCl_3$ (176 MHz).

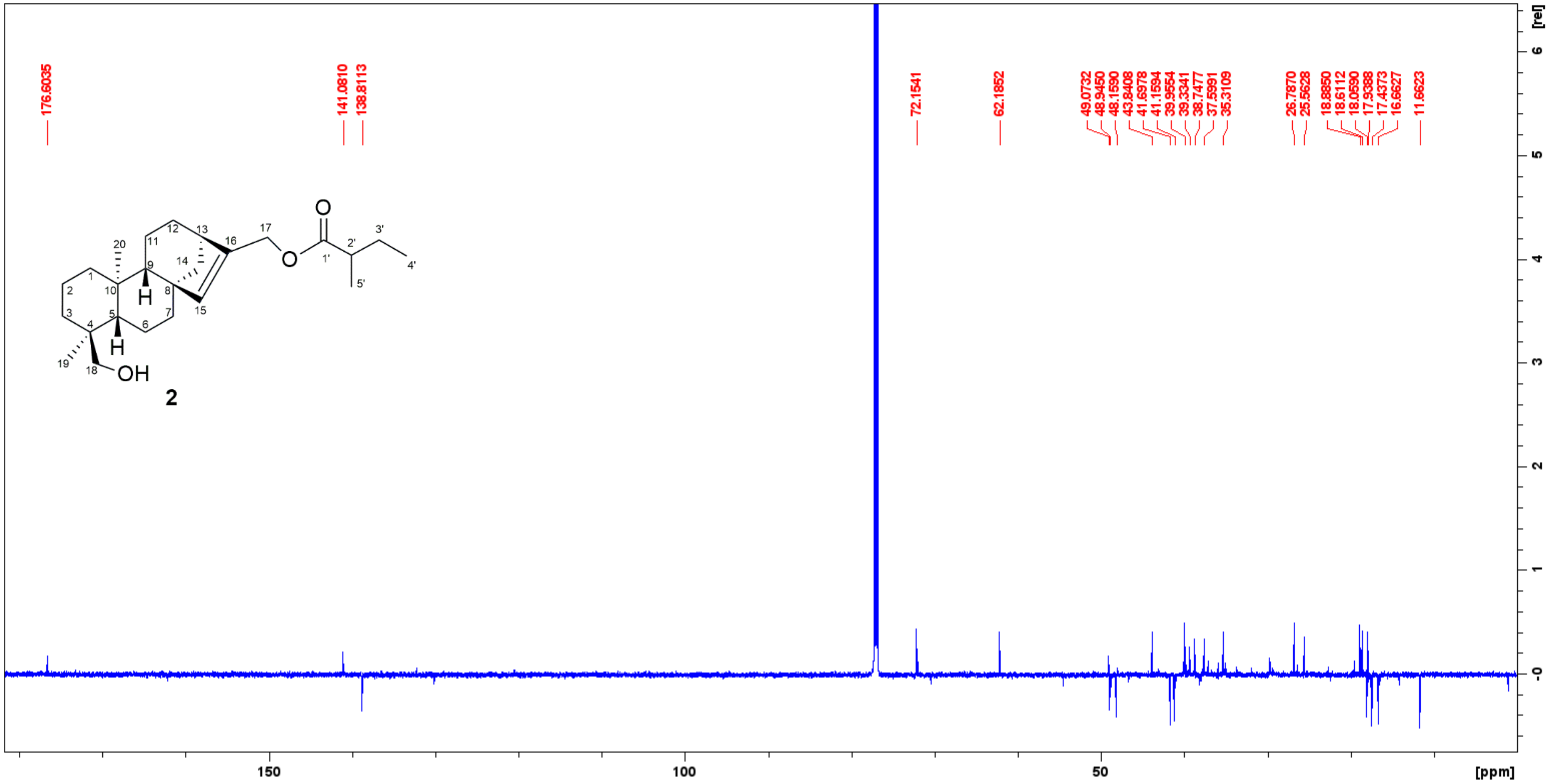

Figure S14. APT NMR spectrum of ferrediol 2-methylbutyrate *(***2***)* in $CDCl_3$ (176 MHz).

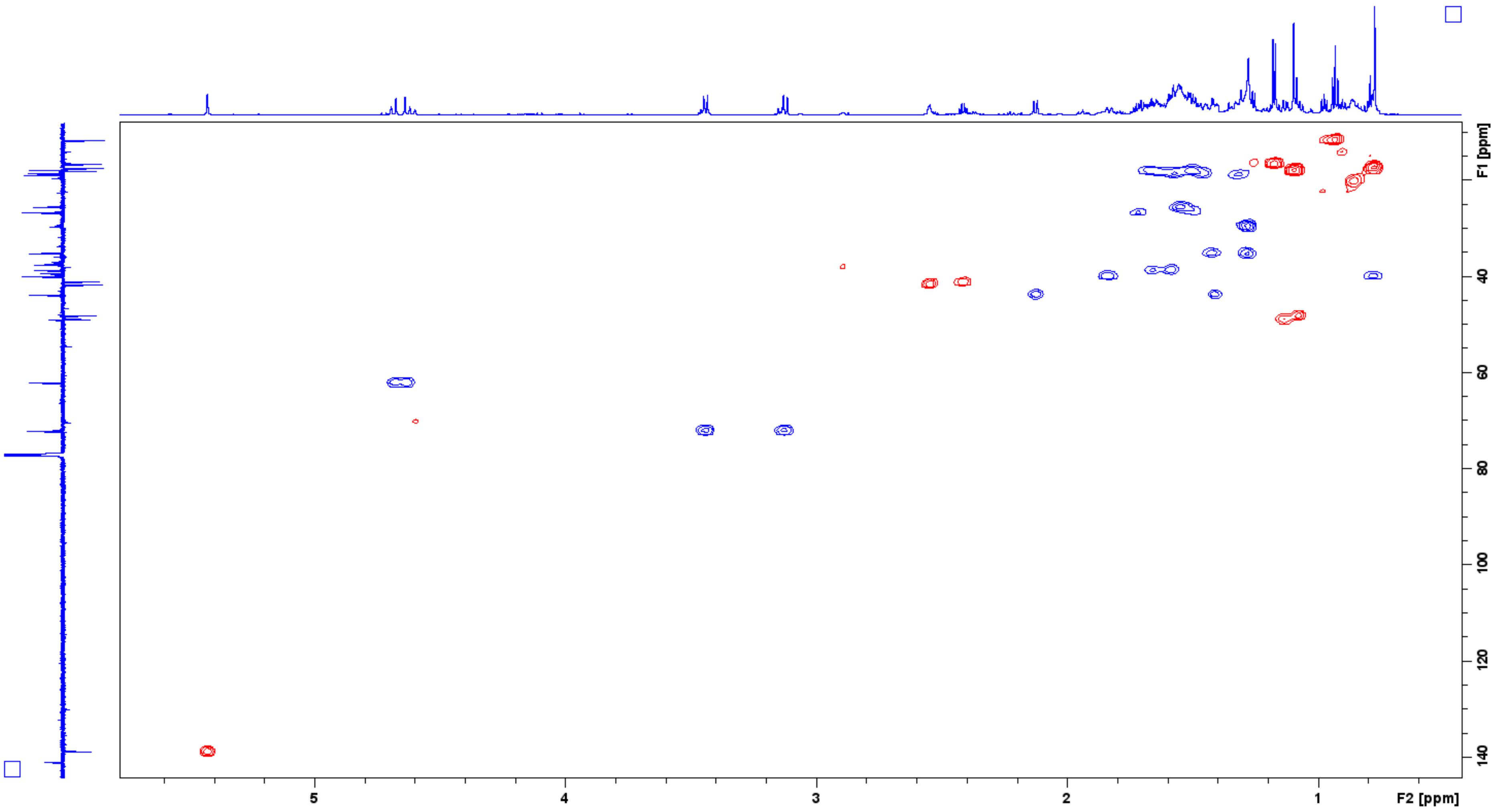

Figure S15. HSQC NMR spectrum of ferrediol 2-methylbutyrate *(***2***)* in $CDCl_3$ (700 MHz)

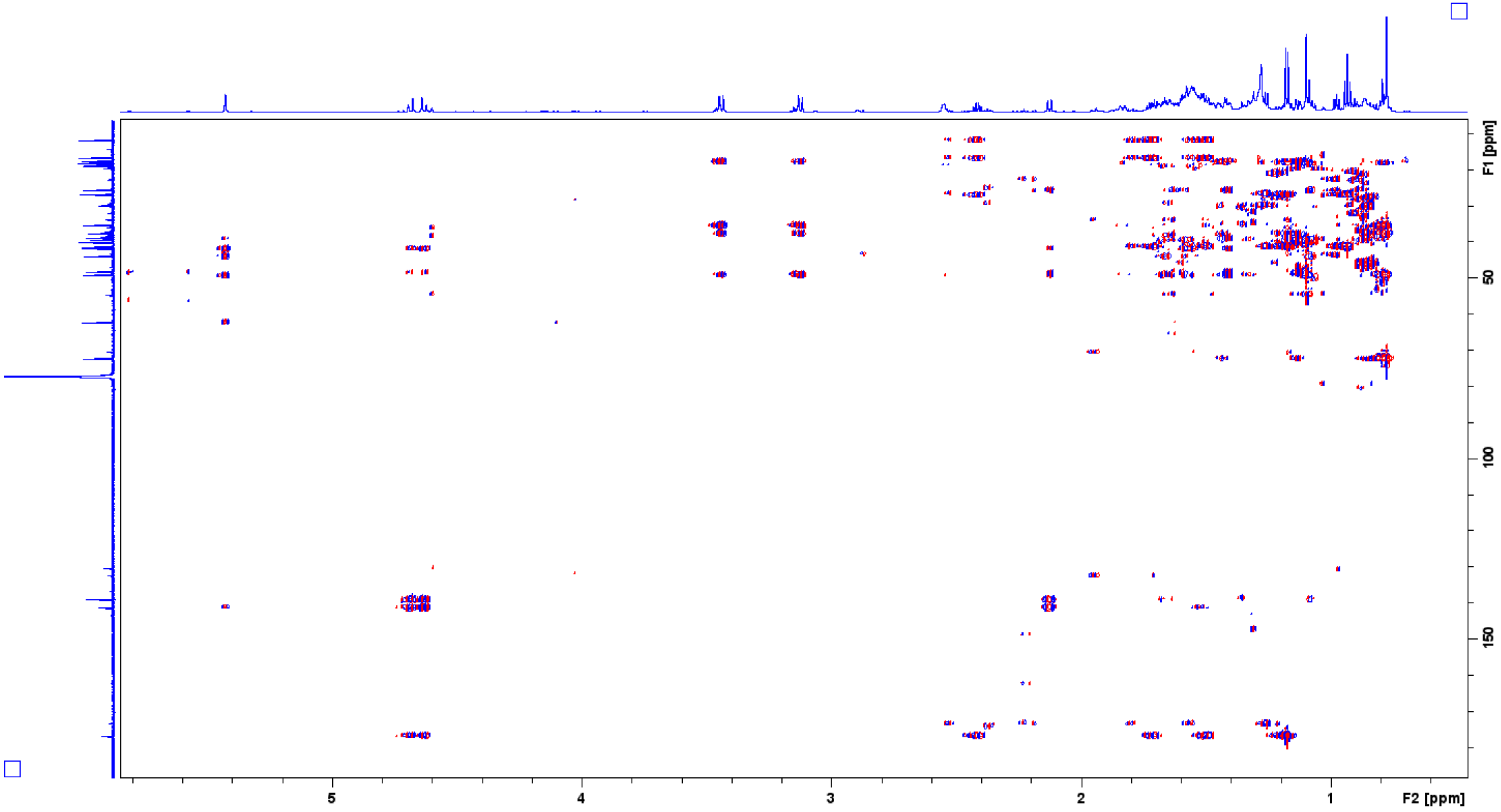

Figure S16. HMBC NMR spectrum of ferrediol 2-methylbutyrate *(***2**) in $CDCl_3$ (700 MHz)

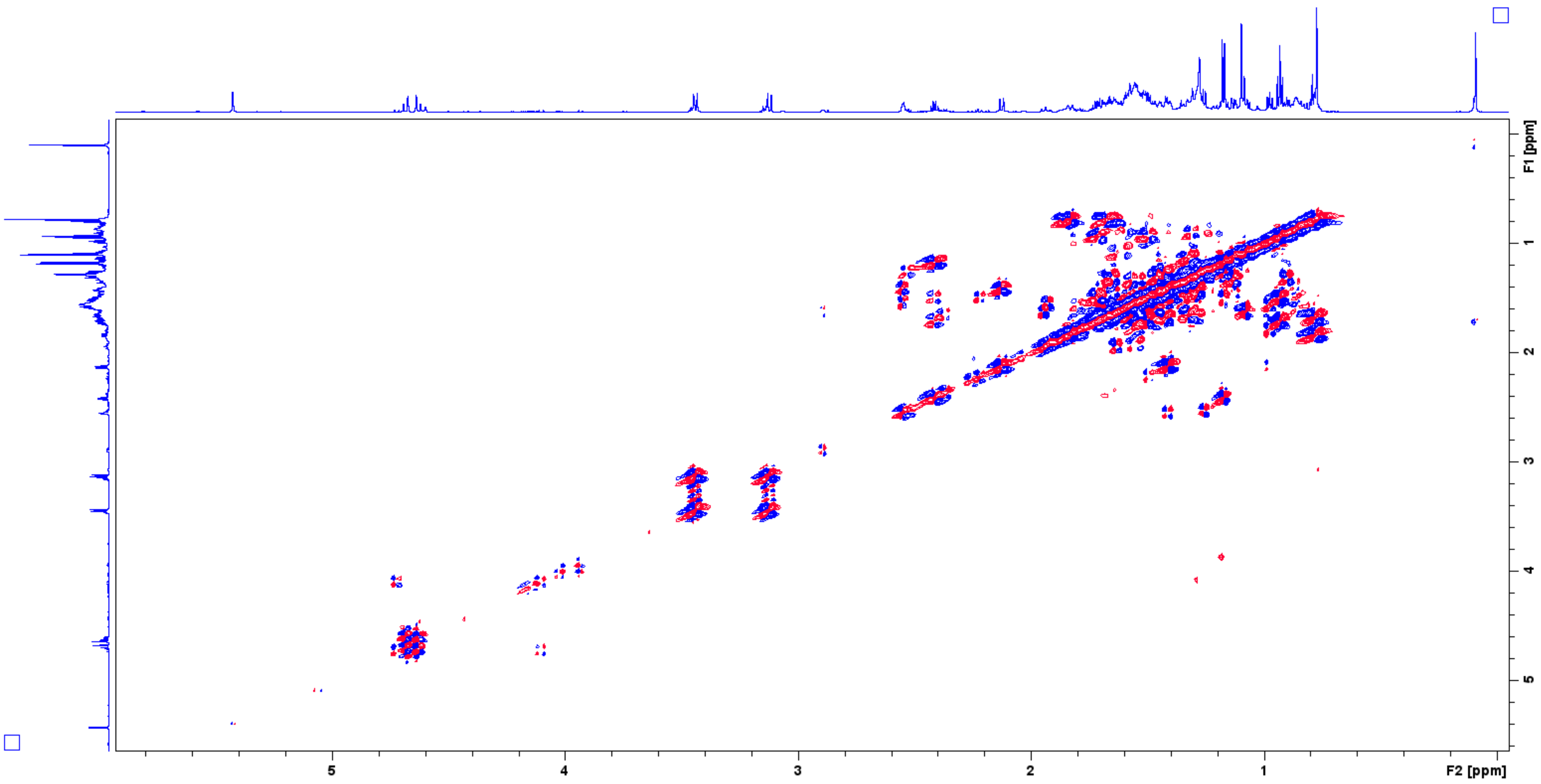

Figure S17. COSY NMR spectrum of ferrediol 2-methylbutyrate *(***2**) in $CDCl_3$ (700 MHz).

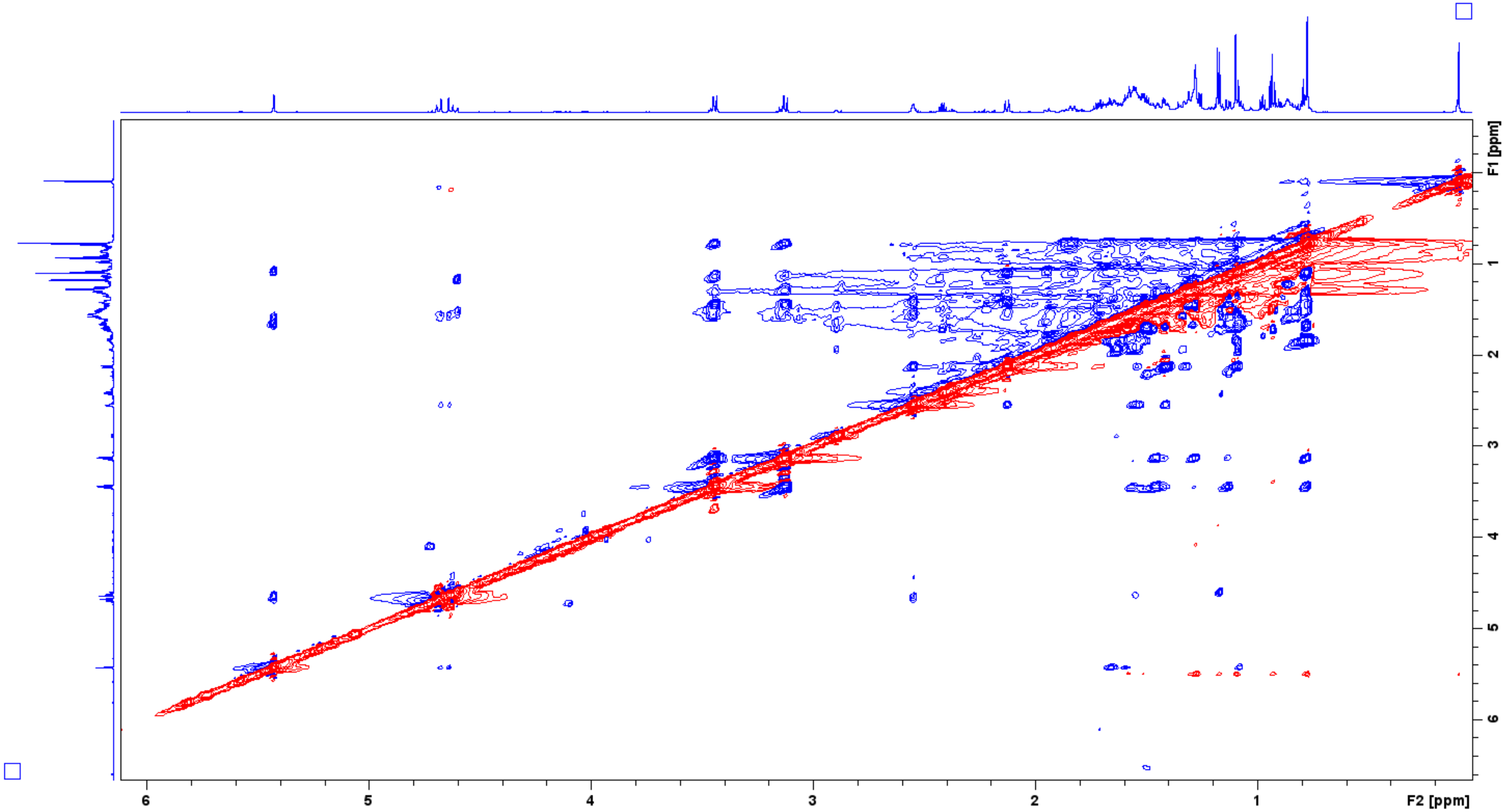

Figure S18. ROESY NMR spectrum of ferrediol 2-methylbutyrate *(***2***)* in $CDCl_3$ (700 MHz).

m/z	Theo. Mass	Delta (ppm)	RDB equiv.	Composition
427.2808	427.2819	-2.48	5.5	C25 H40 O4 Na

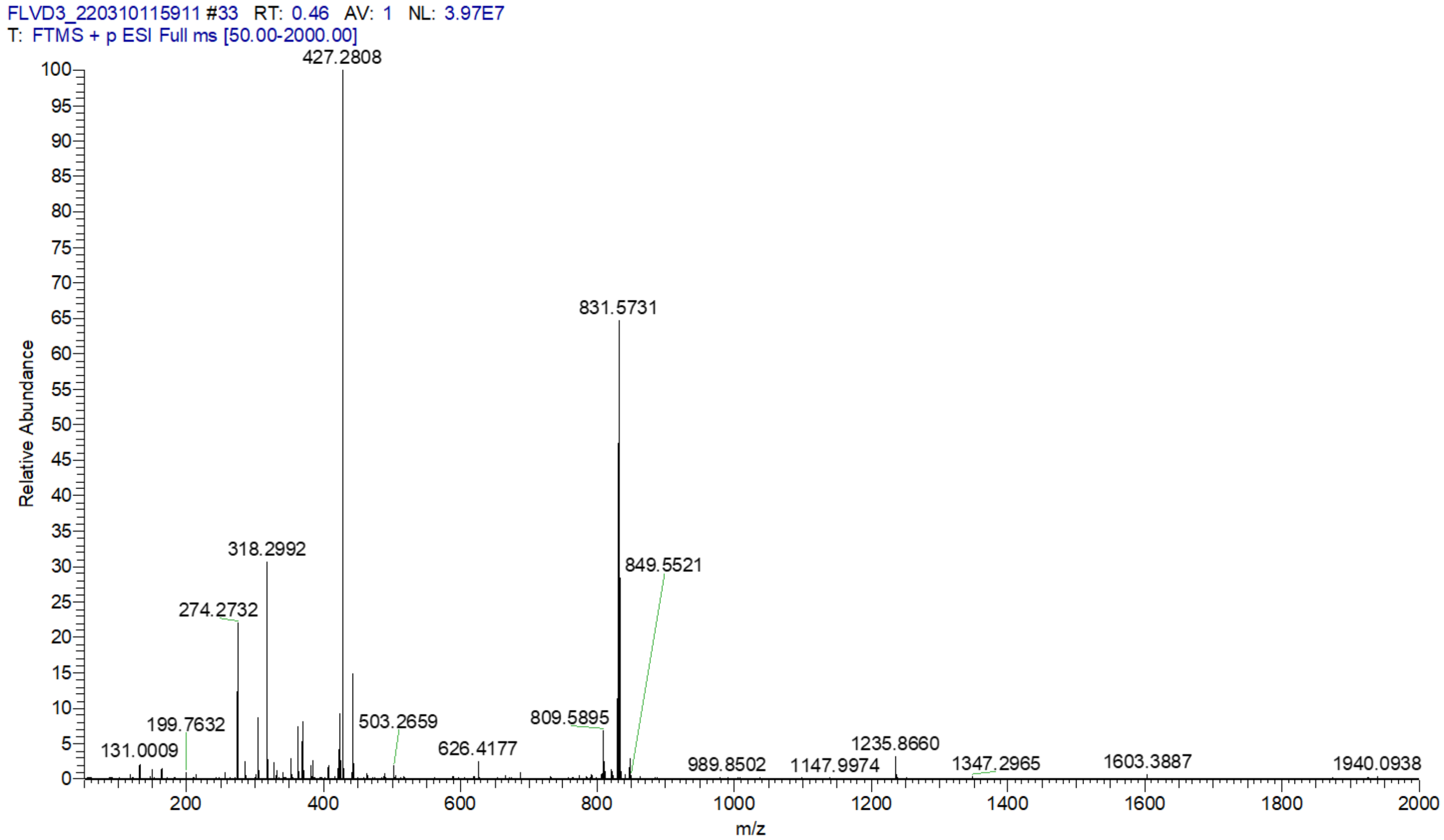

Figure S19. HRESIMS data of sideritriol isovalerate (**5**).

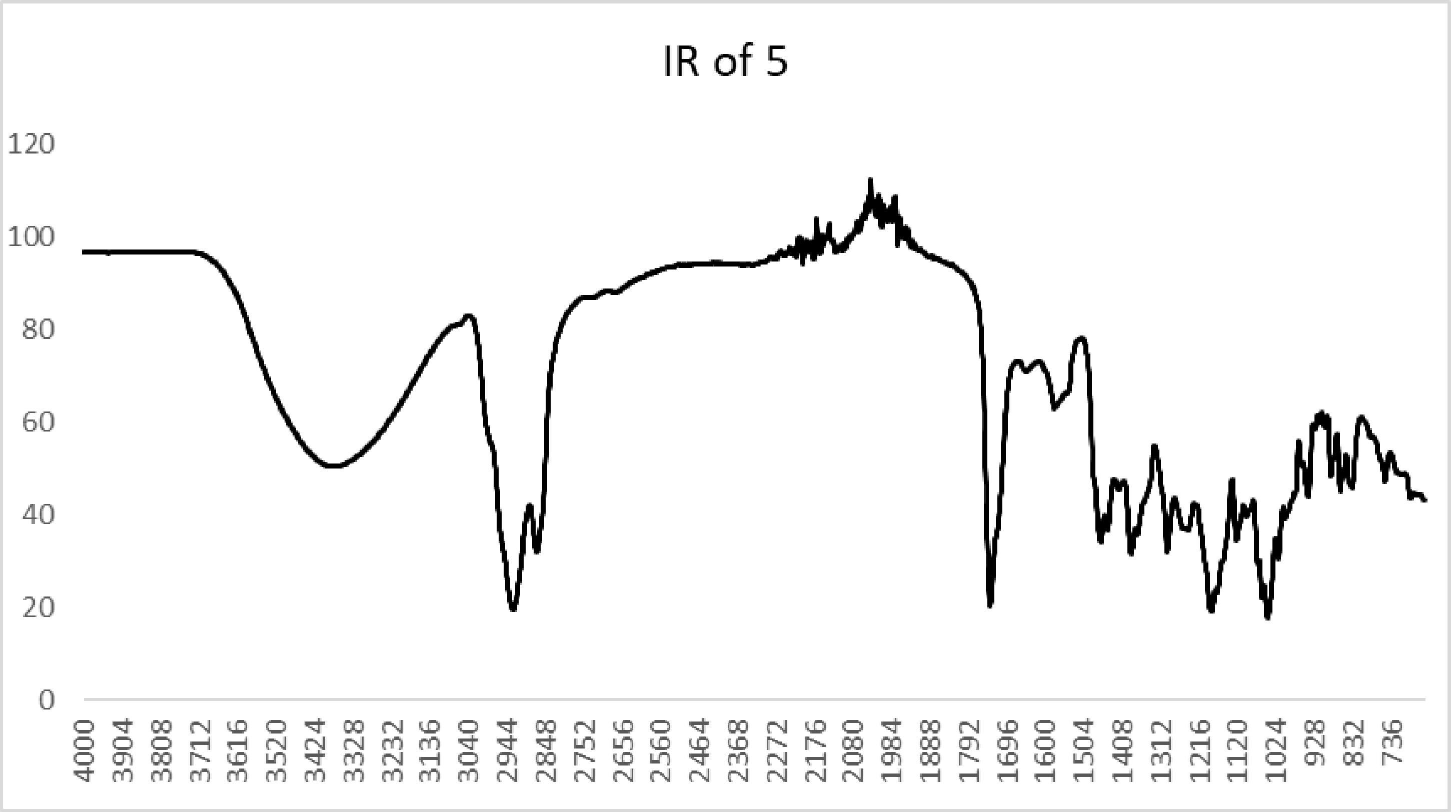

Figure S20: IR spectrum of sideritriol isovalerate (**5**) in methanol.

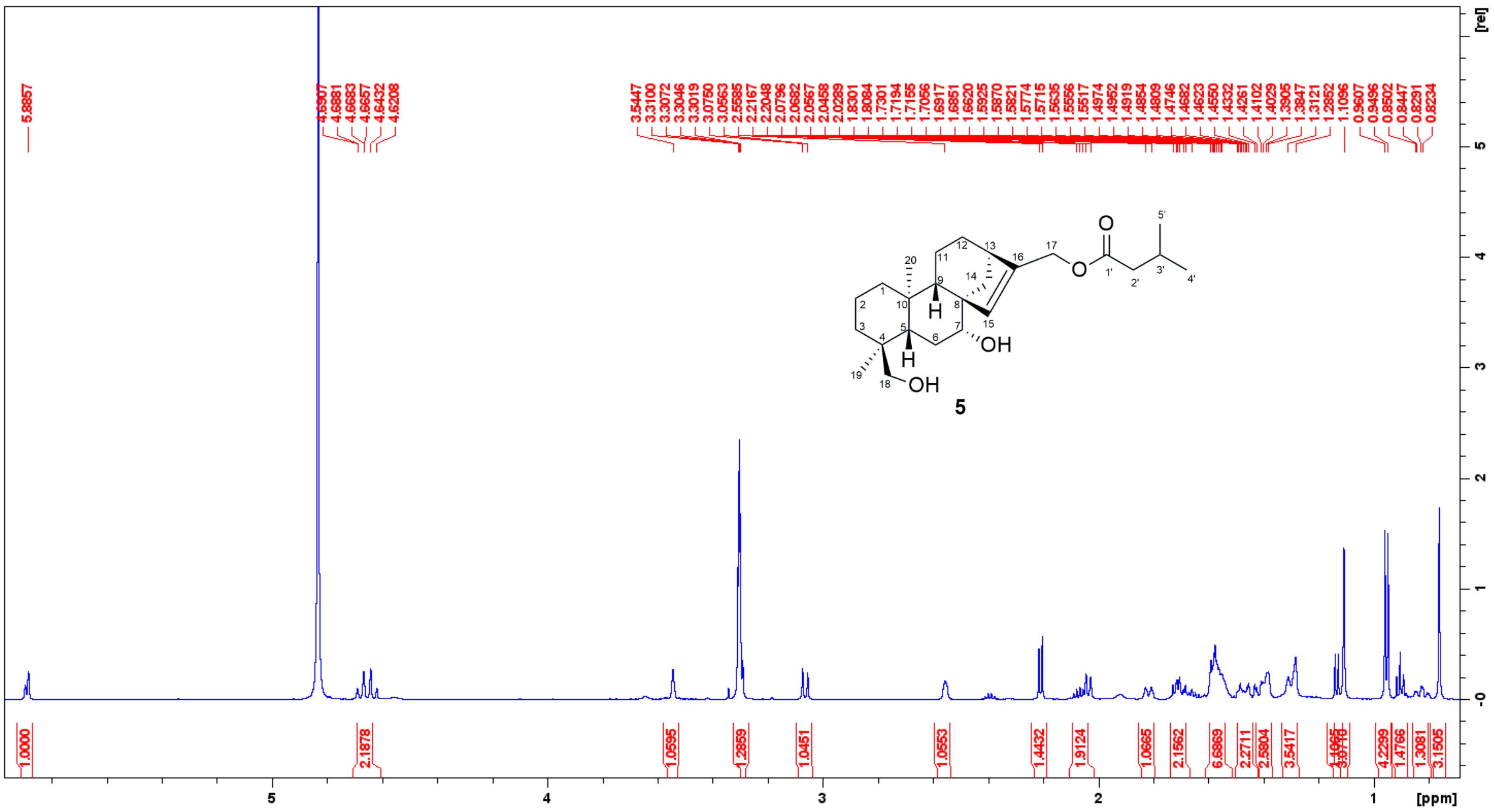

Figure S21. ^{1}H-NMR spectrum of sideritriol isovalerate (**5**) in CD_3OD (600 MHz).

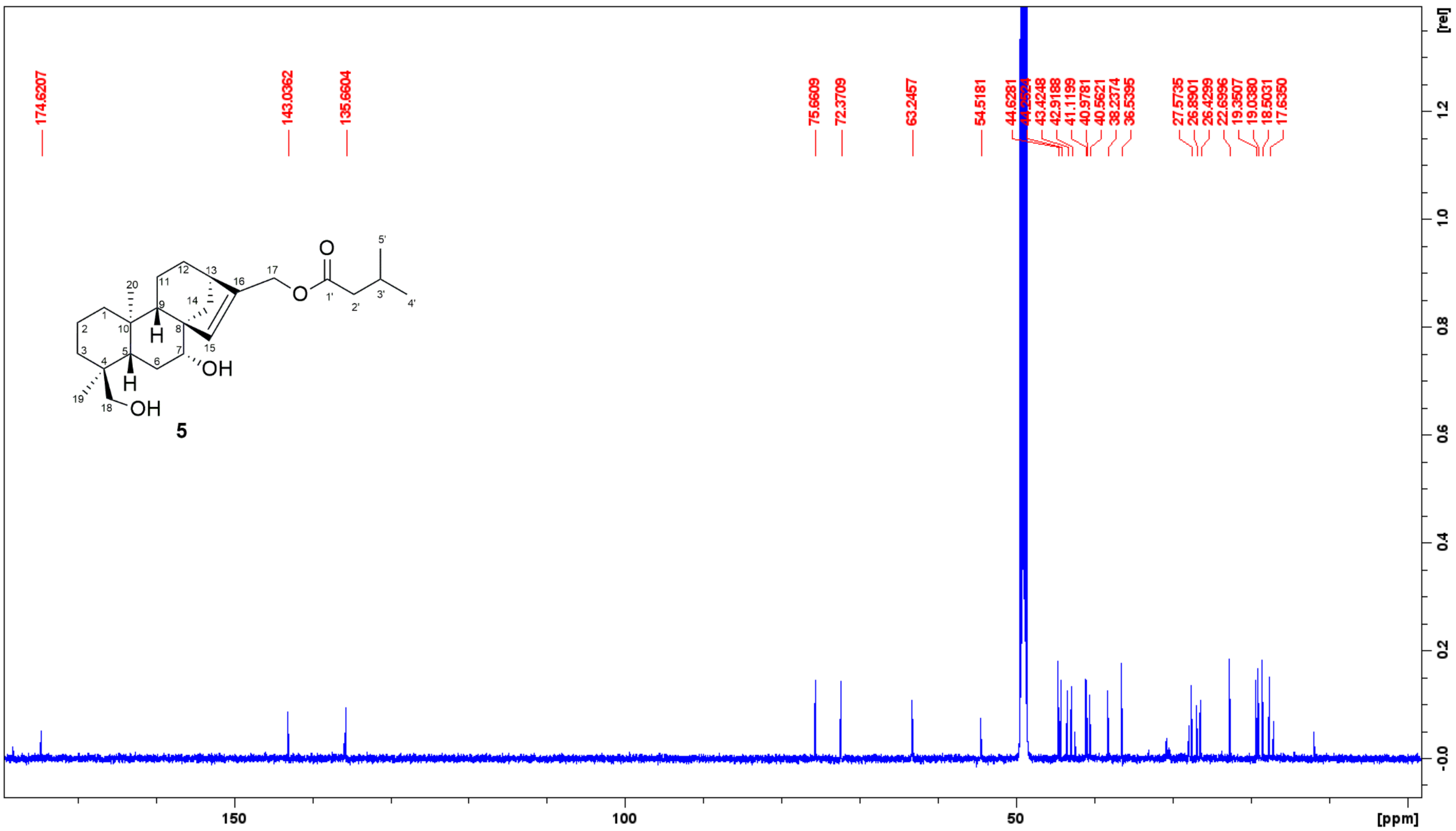

Figure S22. ^{13}C-NMR spectrum of sideritriol isovalerate (**5**) in CD_3OD (150 MHz).

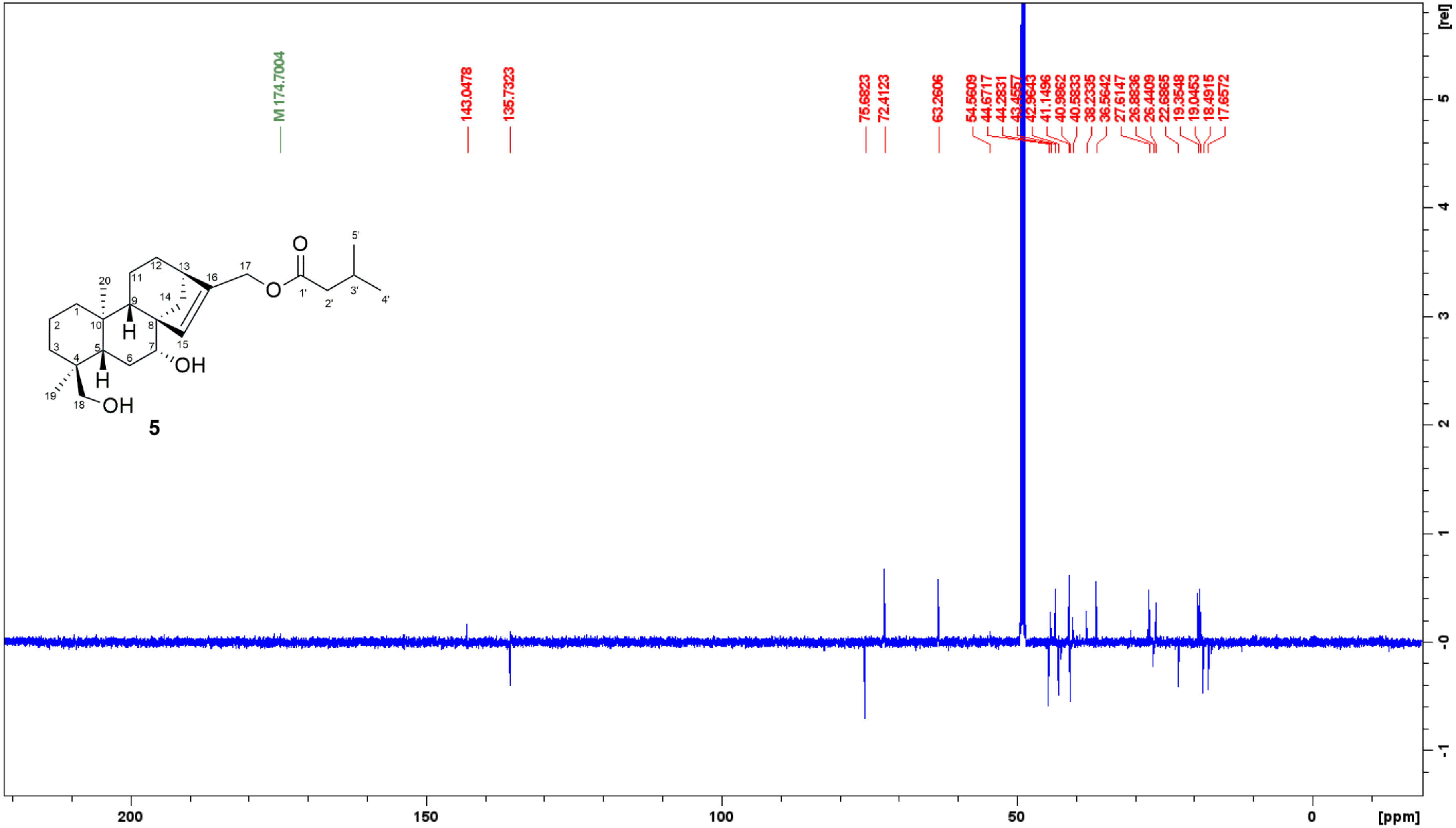

Figure S23. APT NMR spectrum of sideritriol isovalerate (**5**) in CD_3OD (150 MHz).

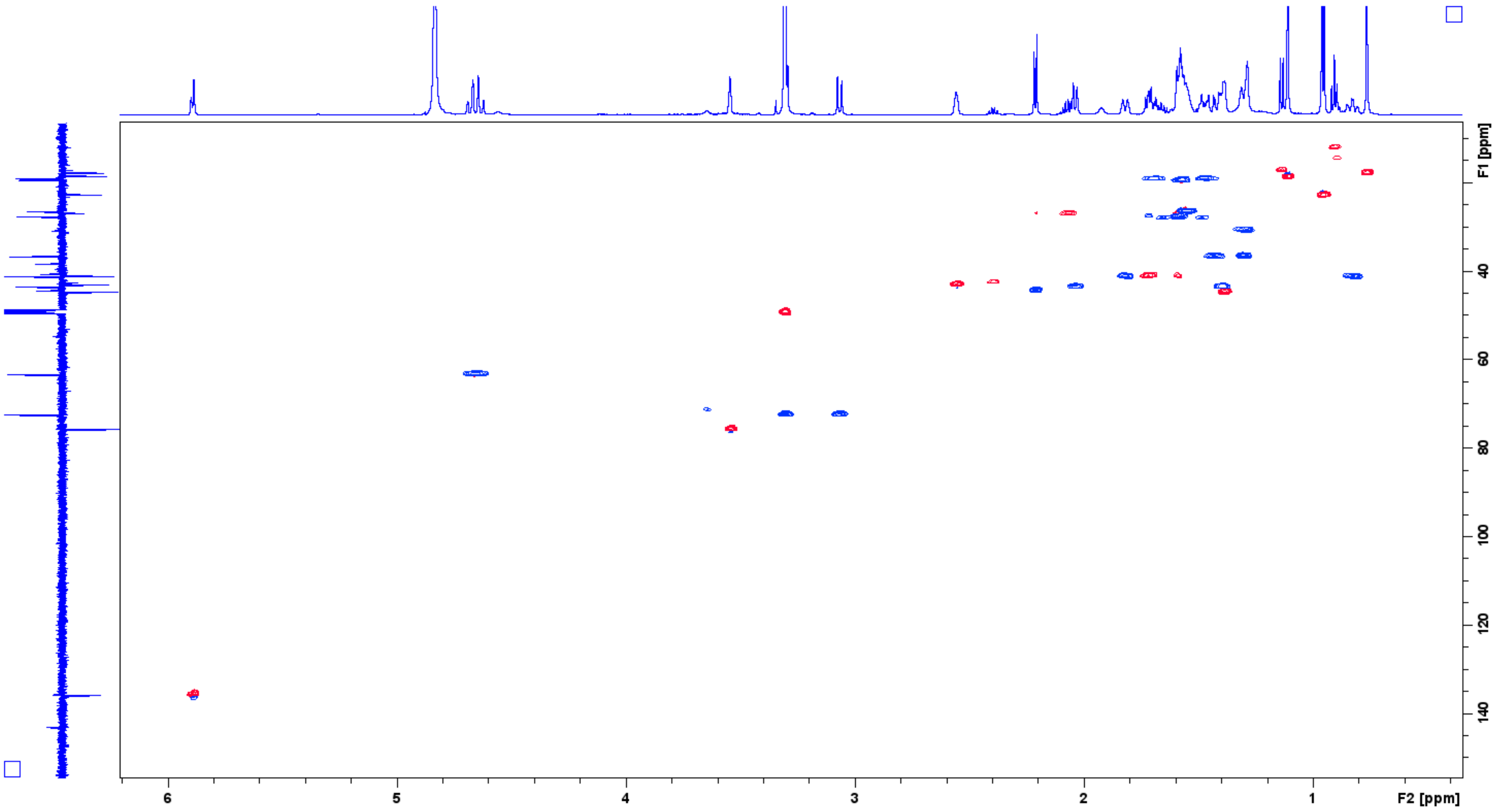

Figure S24. HSQC NMR spectrum of sideritriol isovalerate (**5**) in CD_3OD (600 MHz)

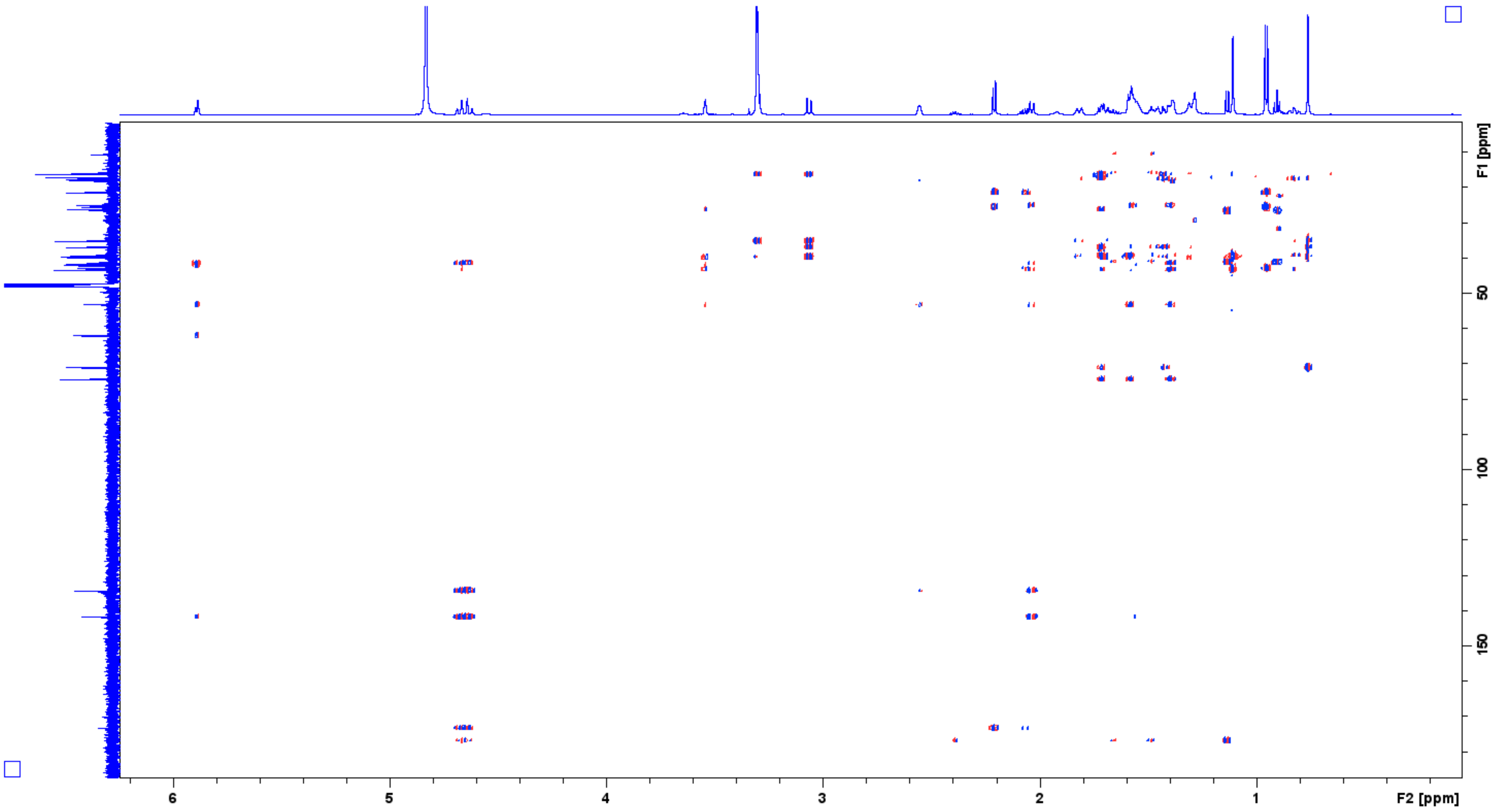

Figure S25. HMBC NMR spectrum of sideritriol isovalerate (**5**) in CD_3OD (600 MHz).

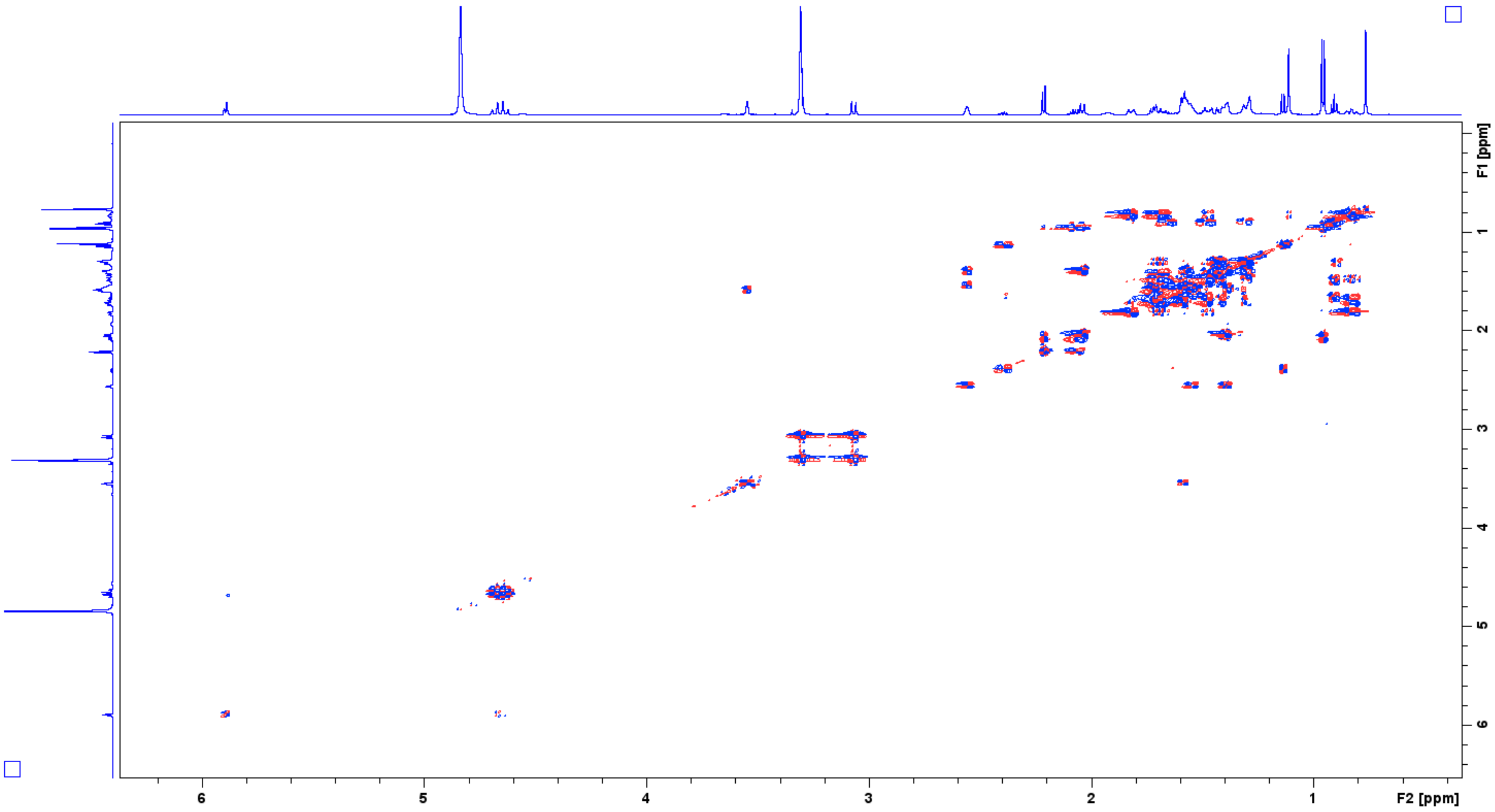

Figure S26. COSY NMR spectrum of sideritriol isovalerate (**5**) in CD_3OD (600 MHz).

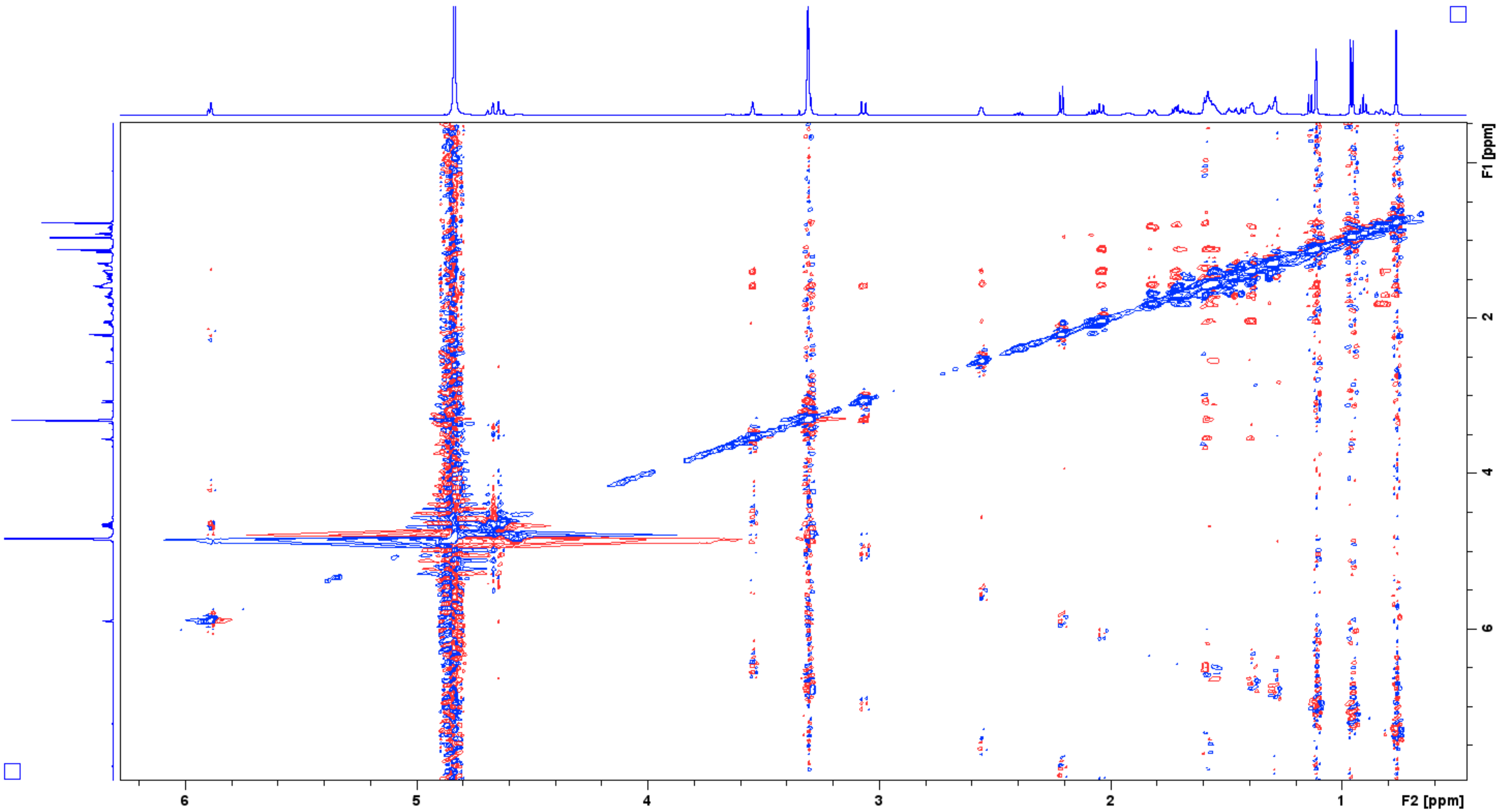

Figure S27. ROESY NMR spectrum of sideritriol isovalerate (**5**) in CD_3OD (600 MHz).

m/z	Theo. Mass	Delta (ppm)	RDB equiv.	Composition
369.2395	369.2400	-1.40	5.5	C22 H34 O3 Na

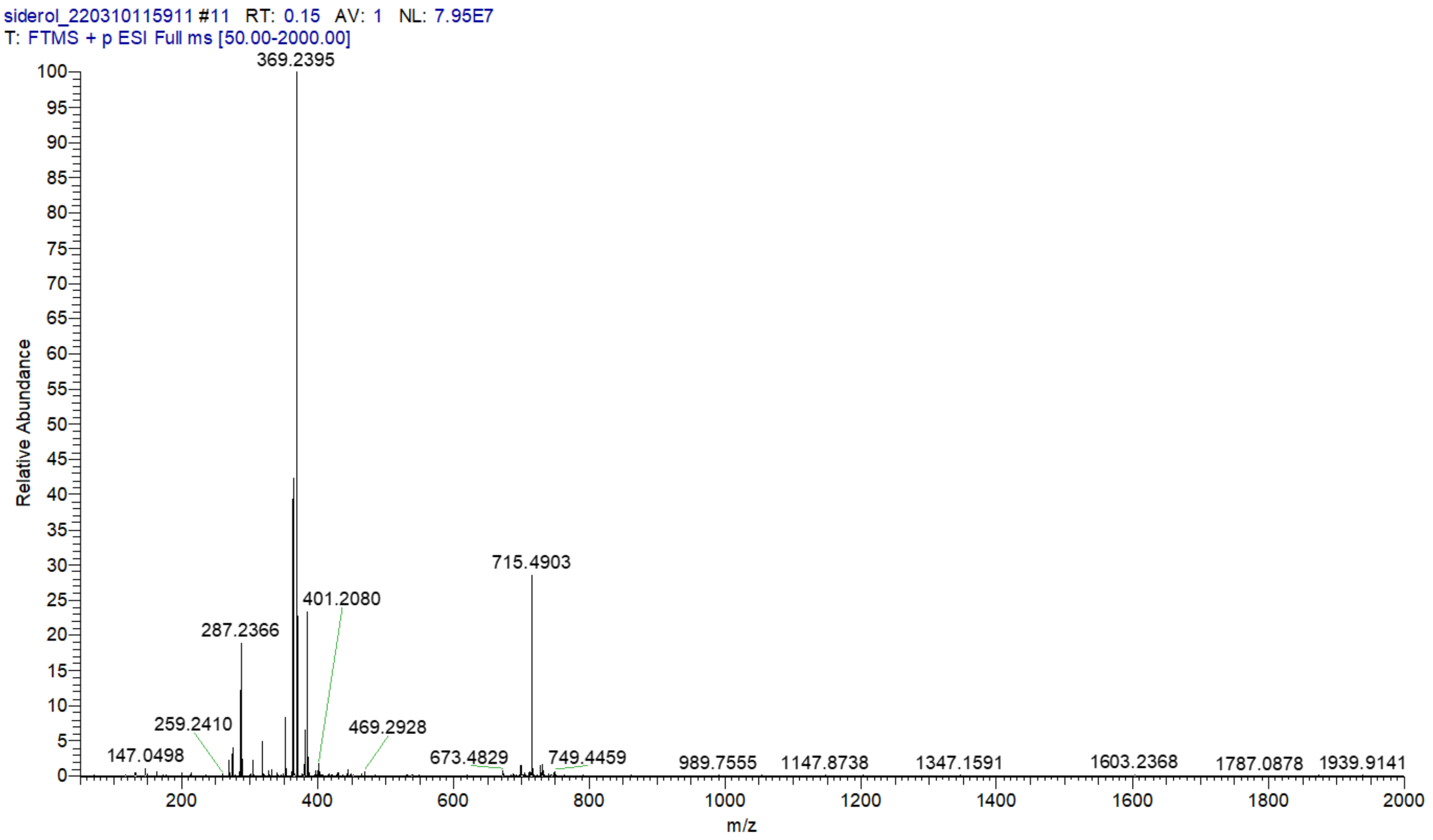

Figure S28. HRESIMS data of siderol (**3**).

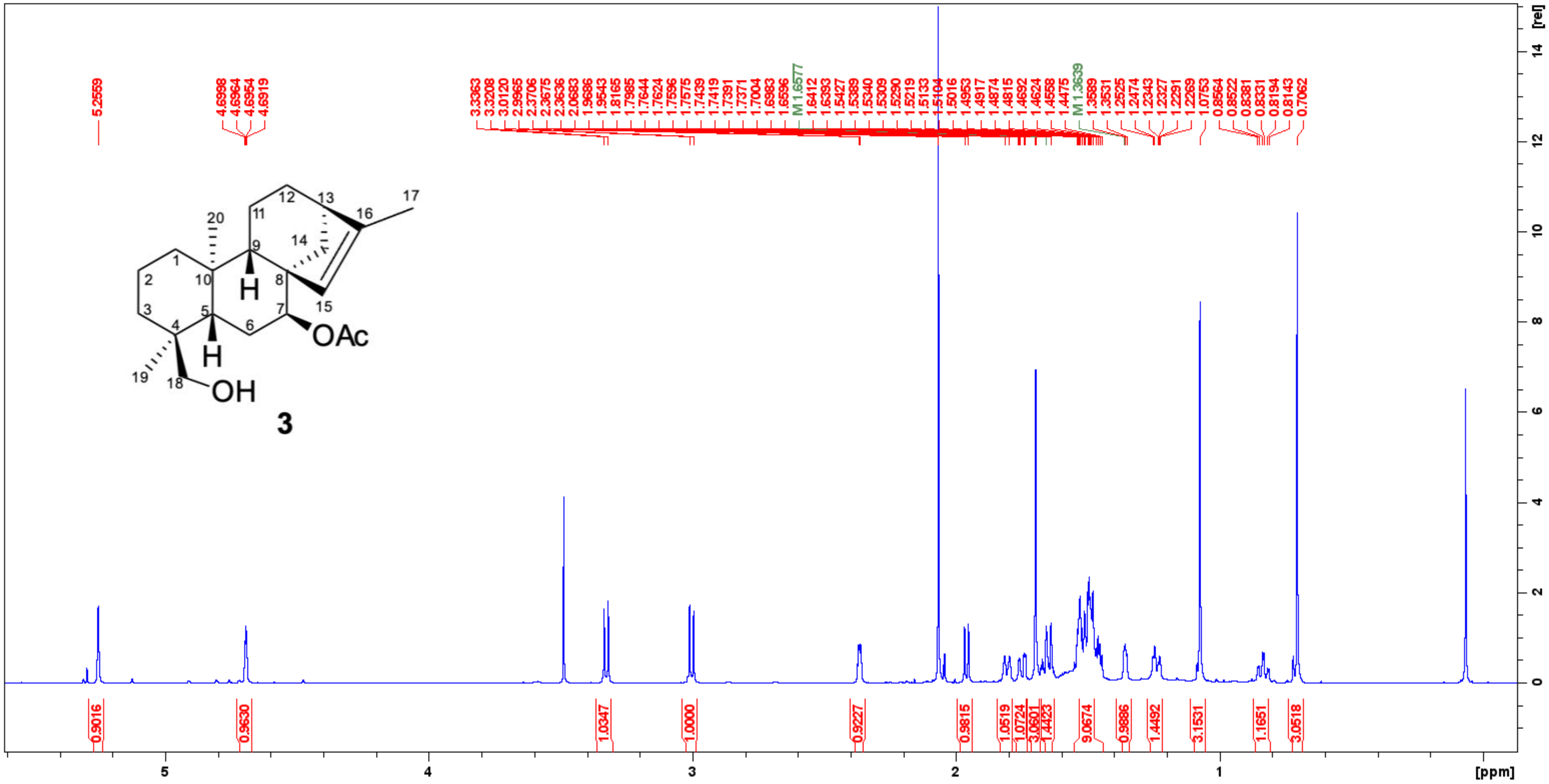

Figure S29. ^{1}H-NMR spectrum of siderol (**3**) in $CDCl_3$ (700 MHz)

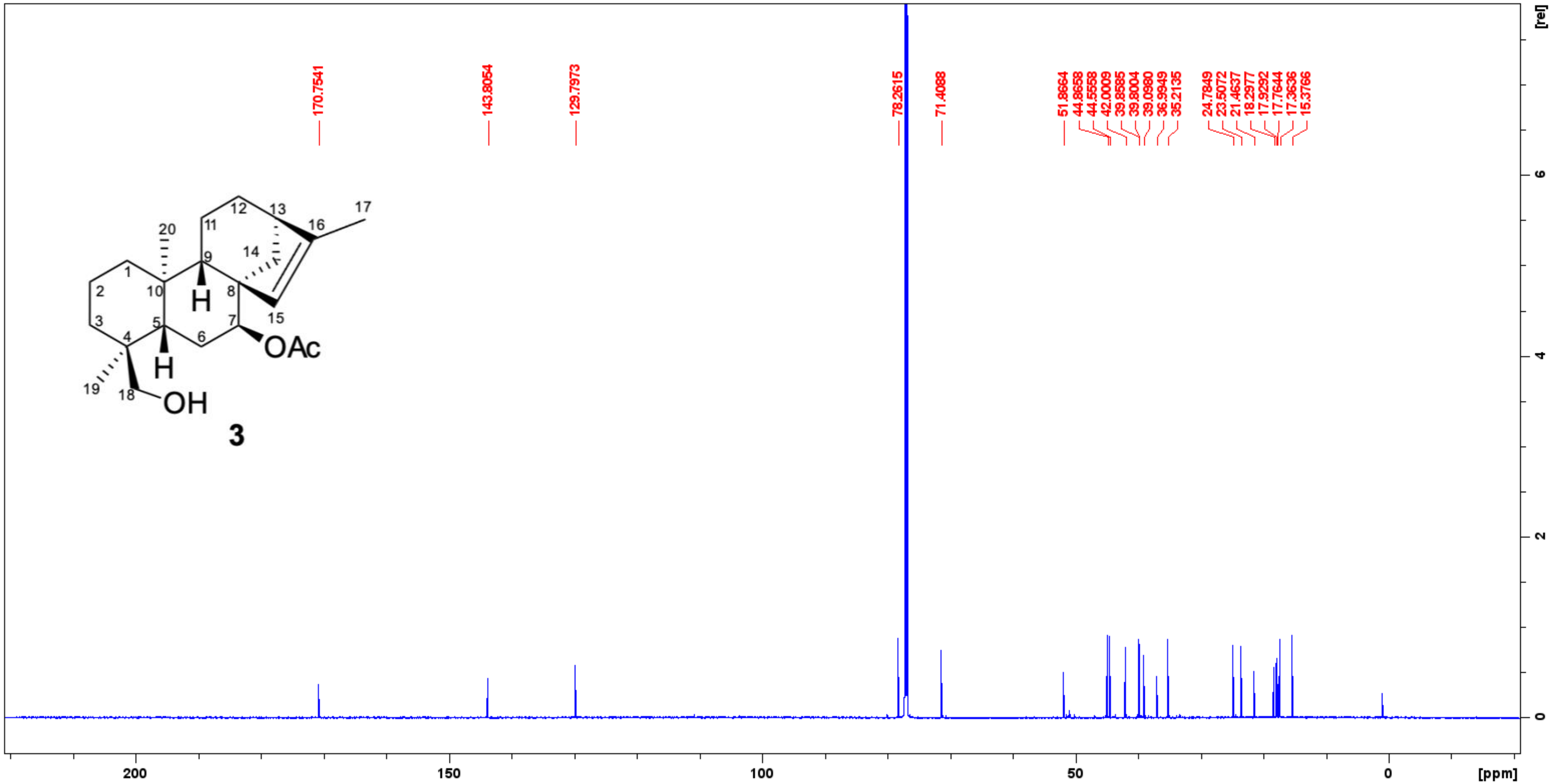

Figure S30. ^{13}C-NMR spectrum of siderol (**3**) in $CDCl_3$ (176 MHz)

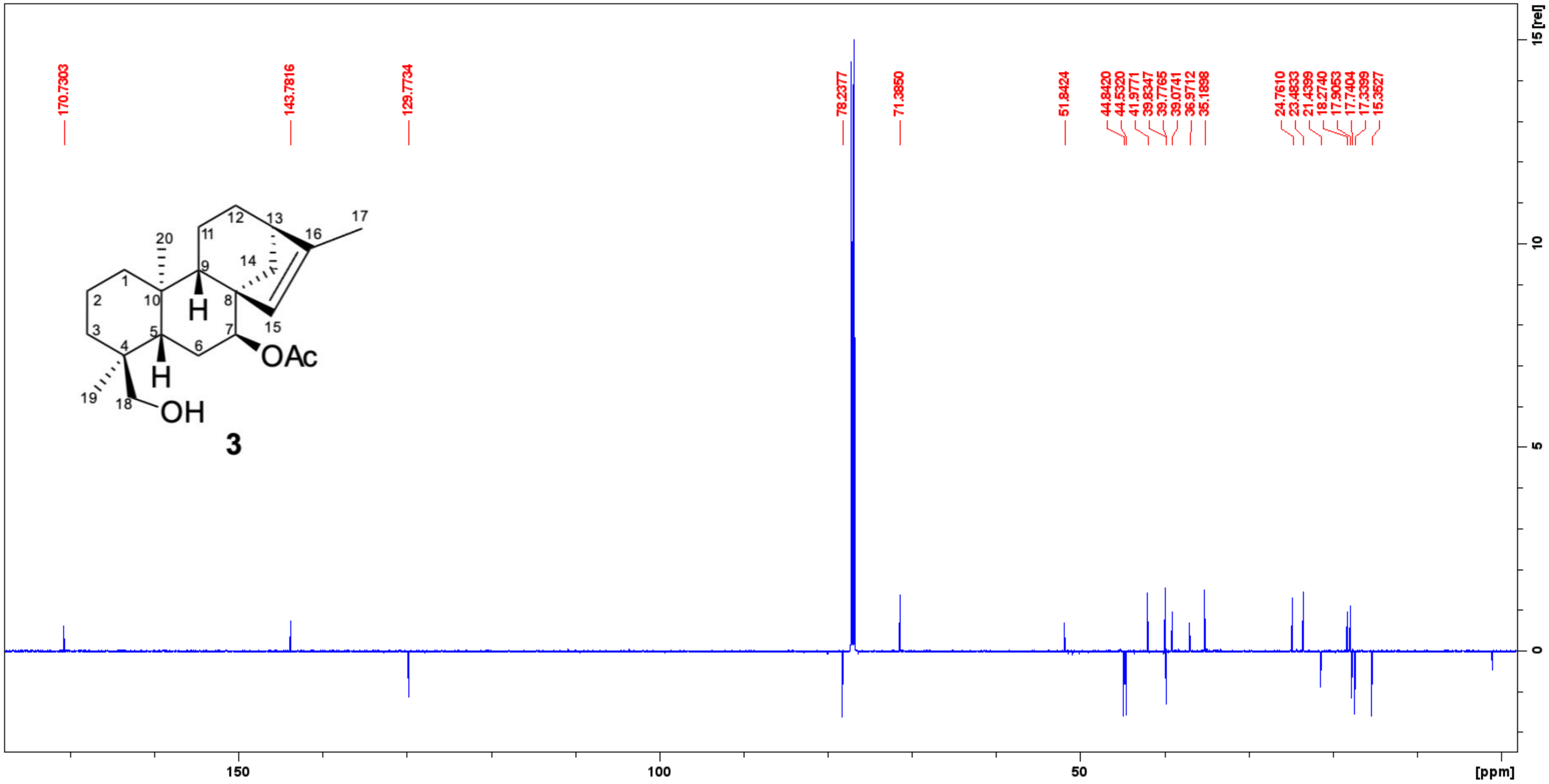

Figure S31. APT NMR spectrum of siderol (**3**) in $CDCl_3$ (176 MHz)

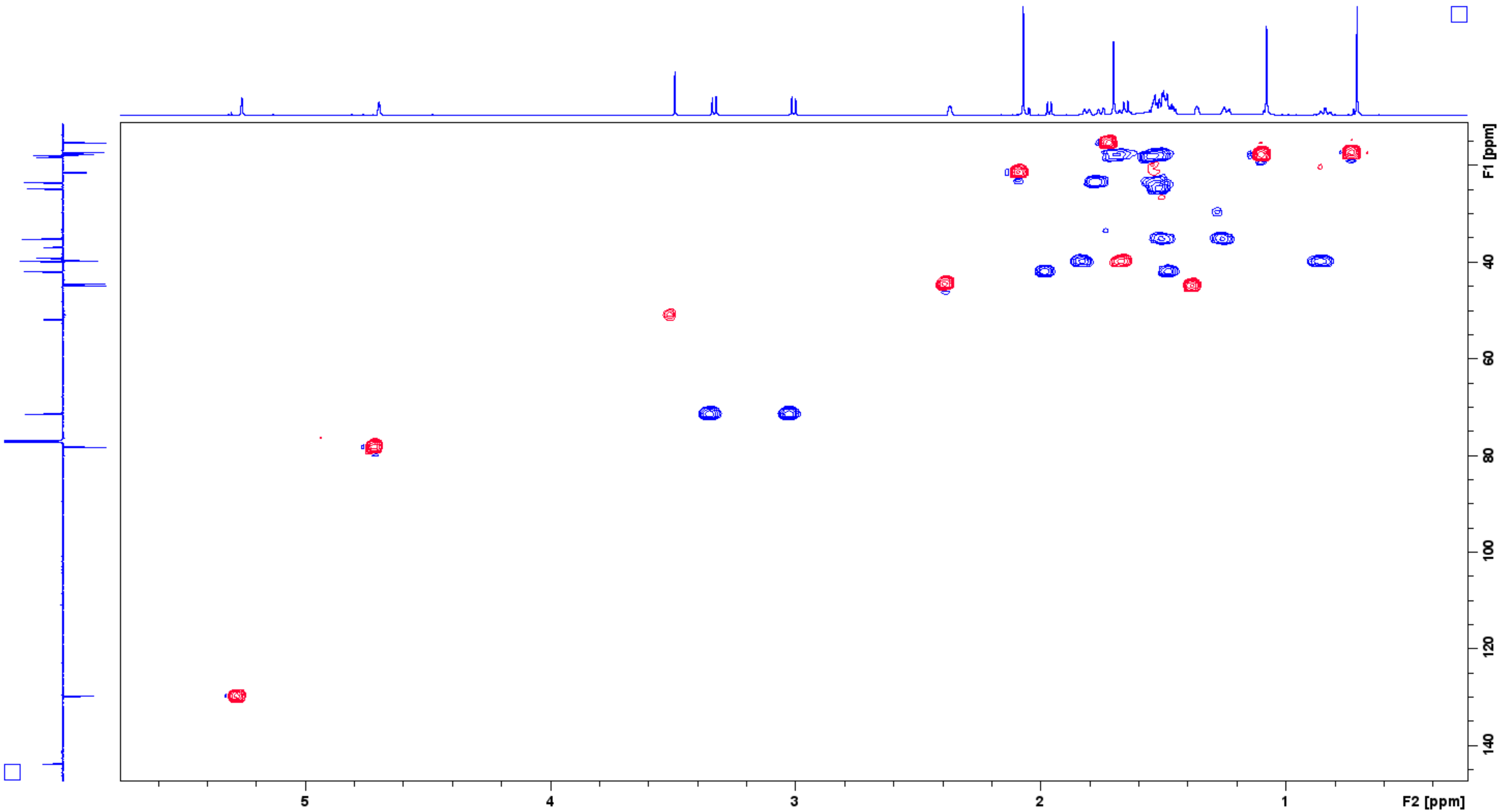

Figure S32. HSQC spectrum of siderol (**3**) in $CDCl_3$ (700 MHz).

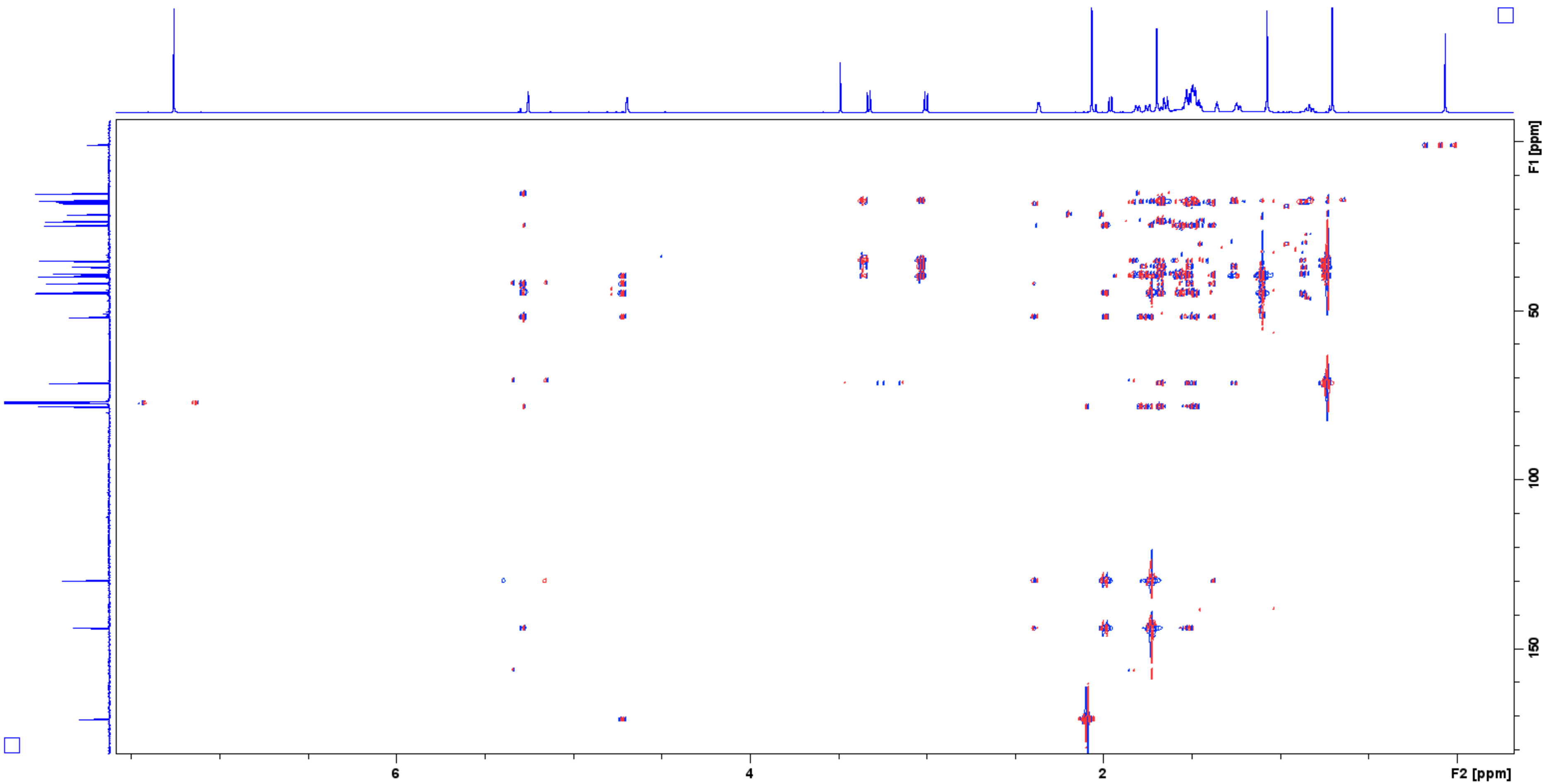

Figure S33. HMBC NMR spectrum of siderol (**3**) in $CDCl_3$ (700 MHz).

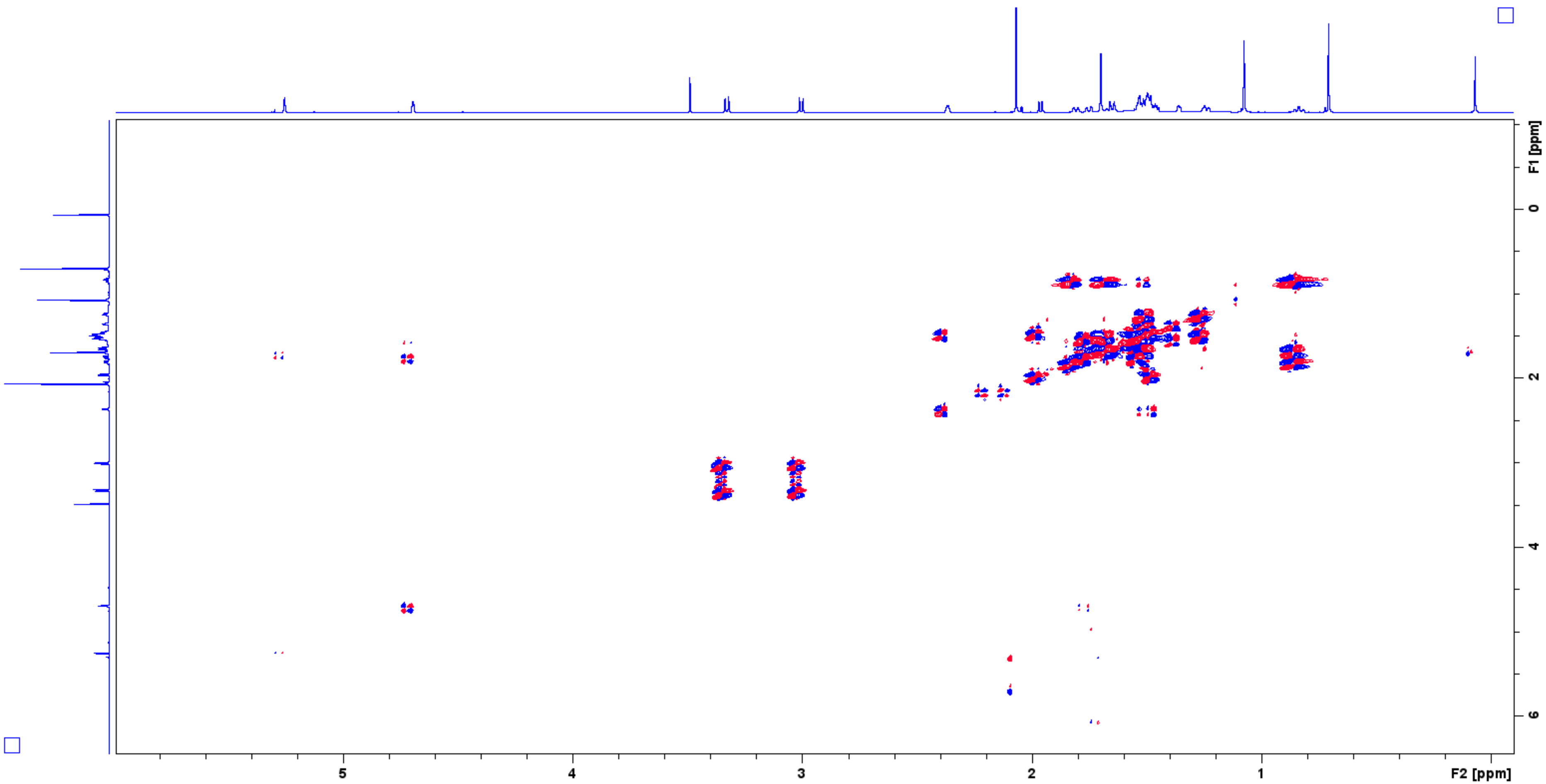

Figure S34. COSY NMR spectrum of siderol (**3**) in $CDCl_3$ (700 MHz).

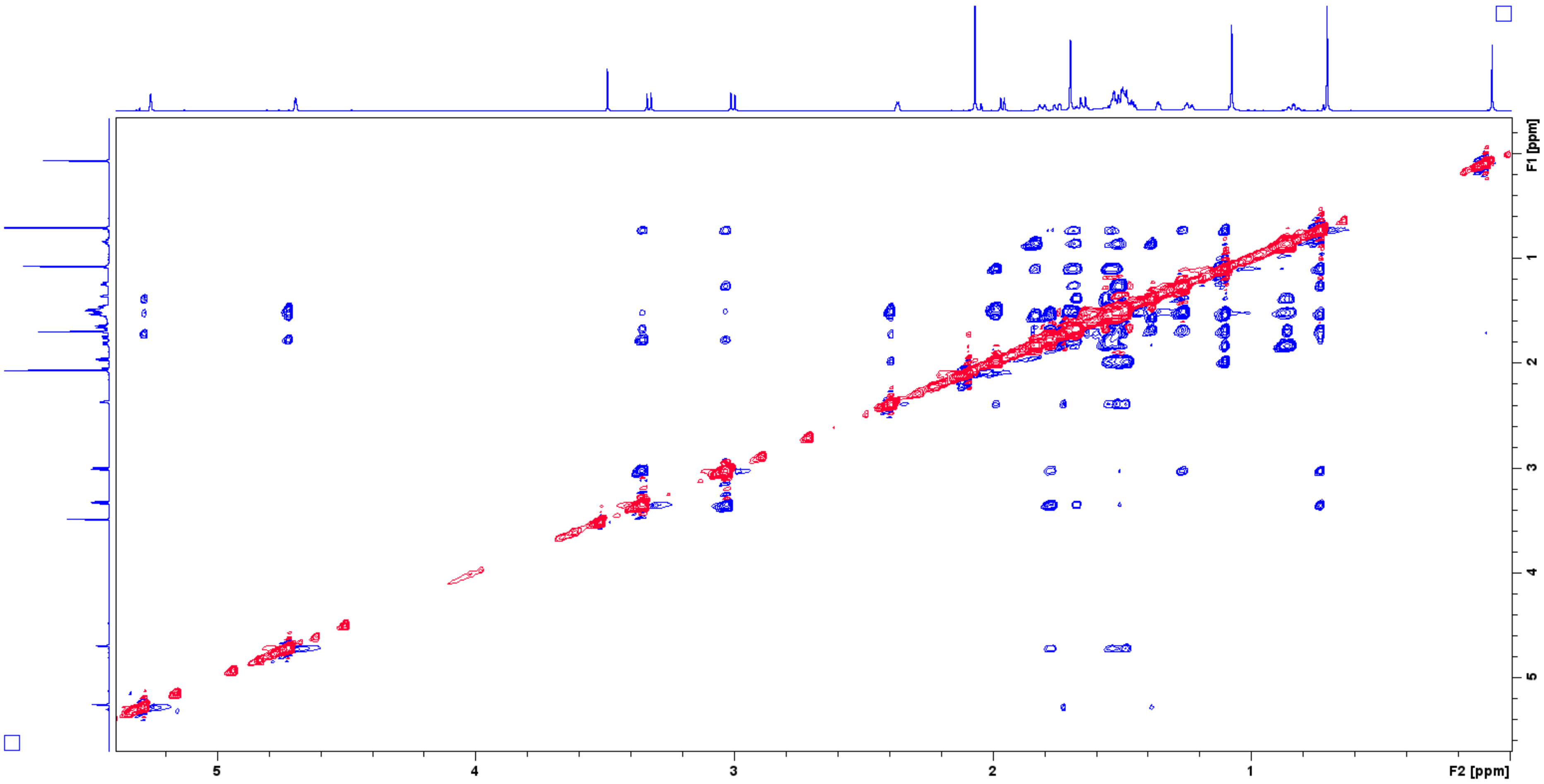

Figure S35. ROESY NMR spectrum of siderol (**3**) in $CDCl_3$ (700 MHz).

m/z	Theo. Mass	Delta (ppm)	RDB equiv.	Composition
327.2289	327.2295	-1.65	4.5	C20 H32 O2 Na

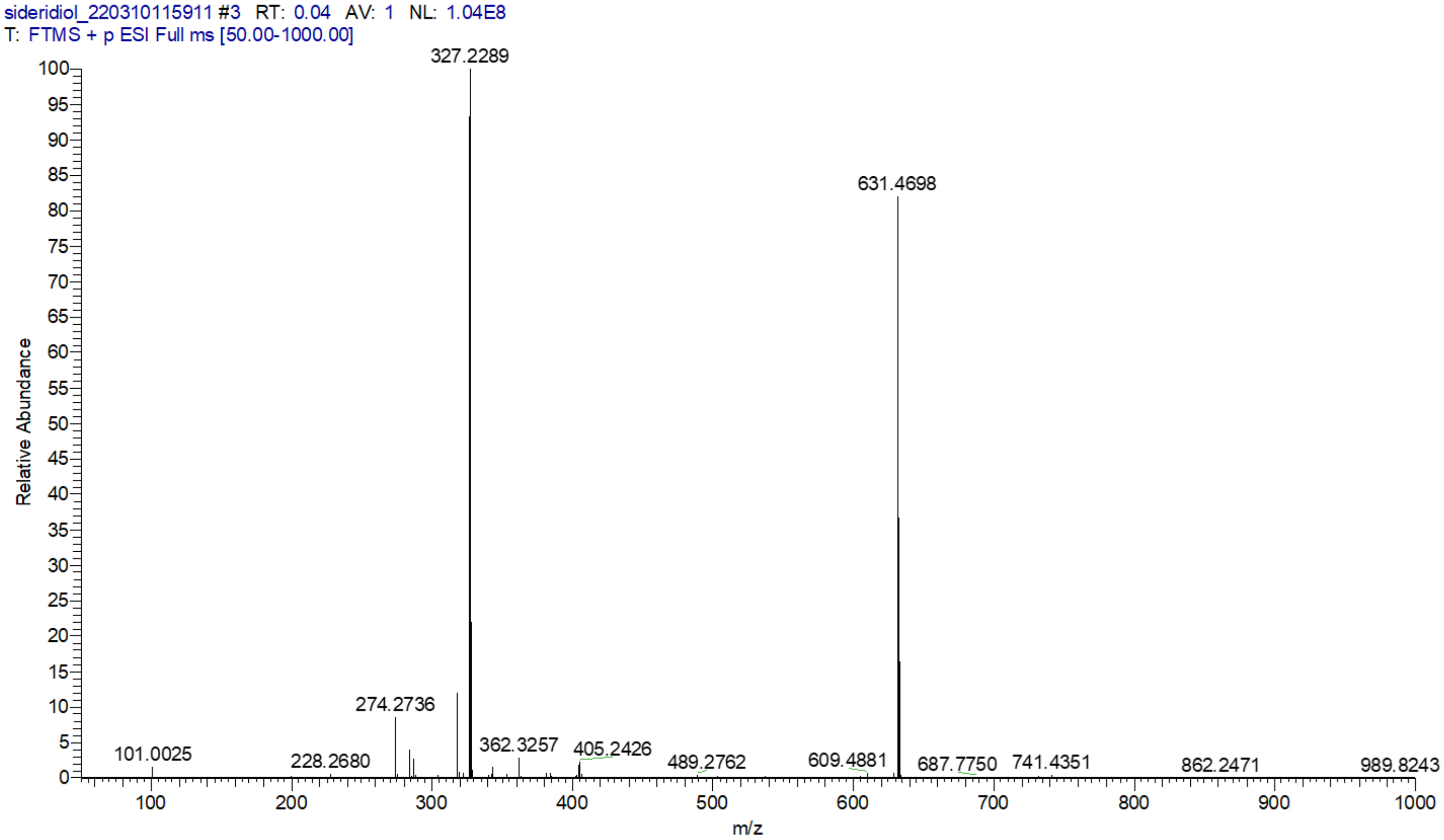

Figure S36. HRESIMS data of sideridiol (**4**)

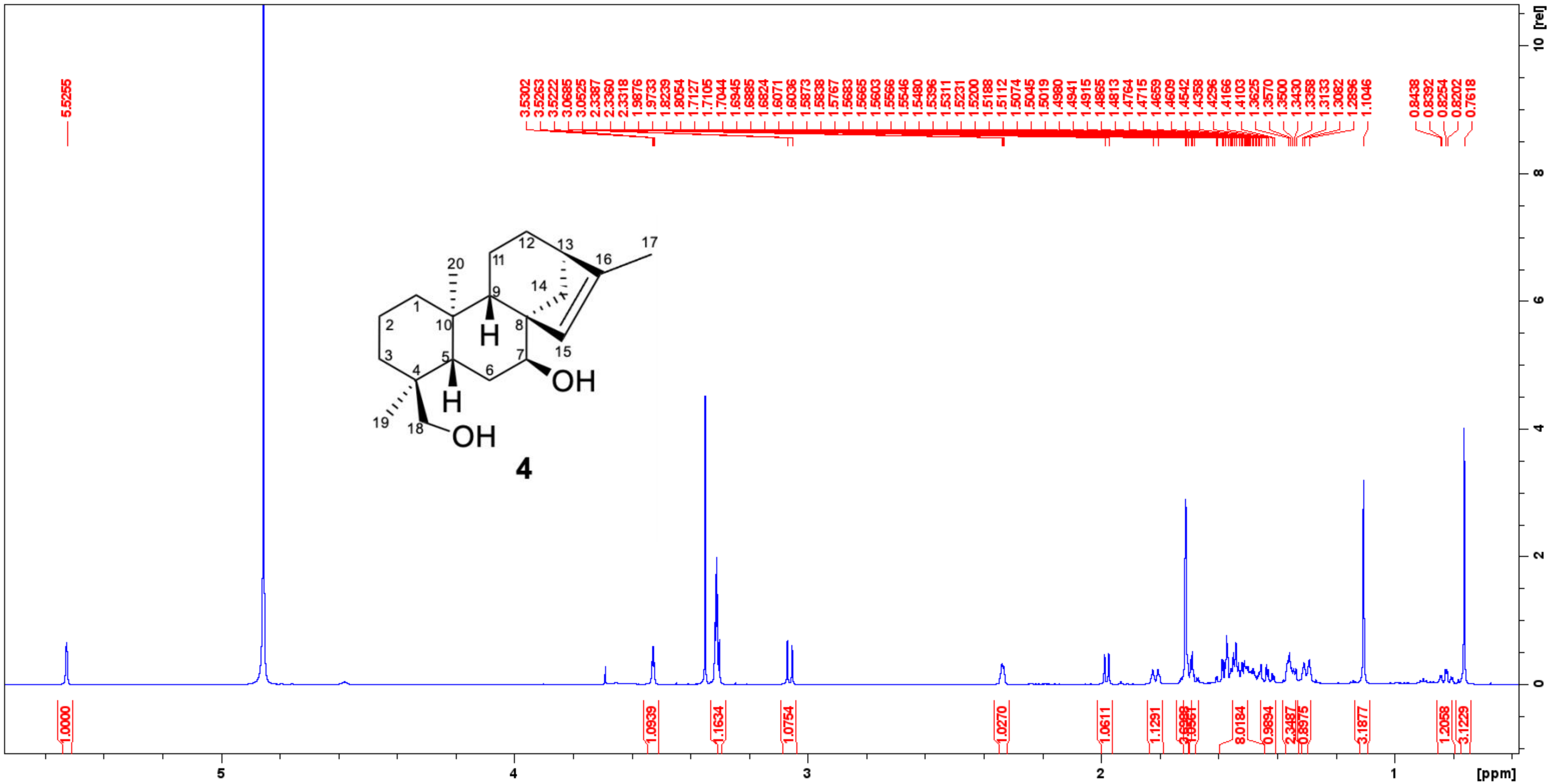

Figure S37. ^{1}H-NMR spectrum of sideridiol (**4**) in CD_3OD (700 MHz).

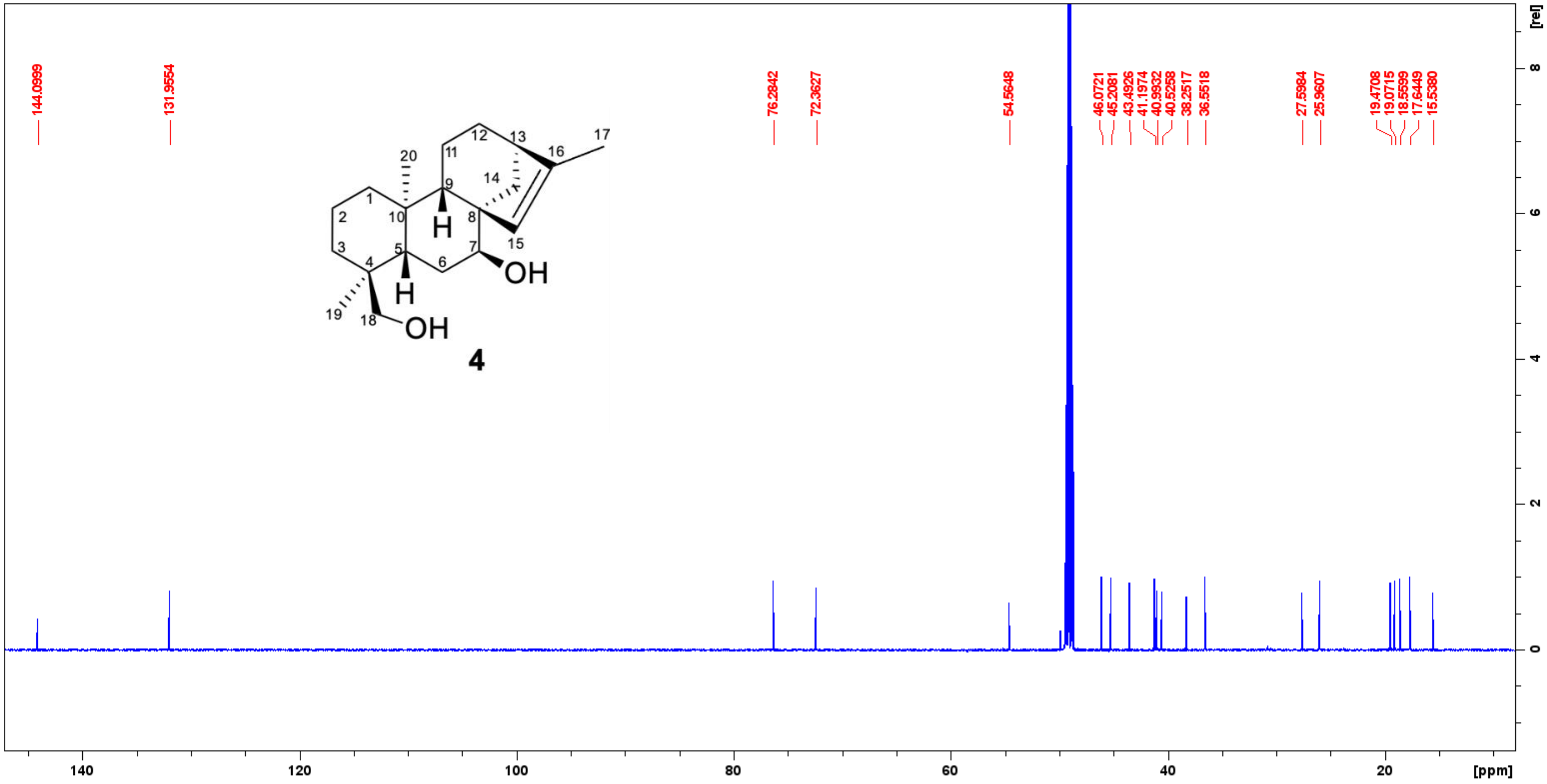

Figure S38. ^{13}C-NMR spectrum of sideridiol (**4**) in CD_3OD (176 MHz).

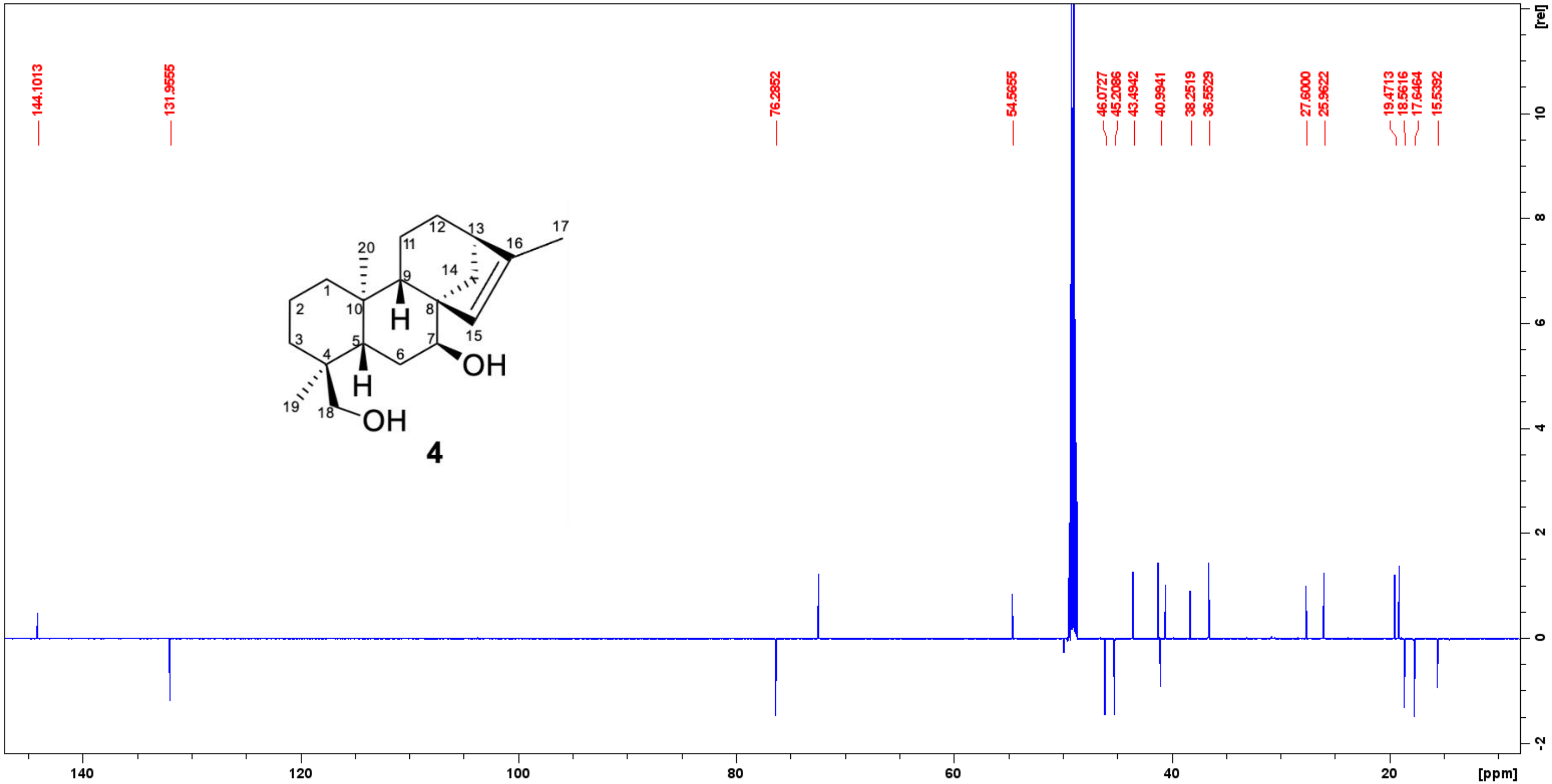

Figure S39. APT NMR spectrum of sideridiol (**4**) in CD_3OD (176 MHz).

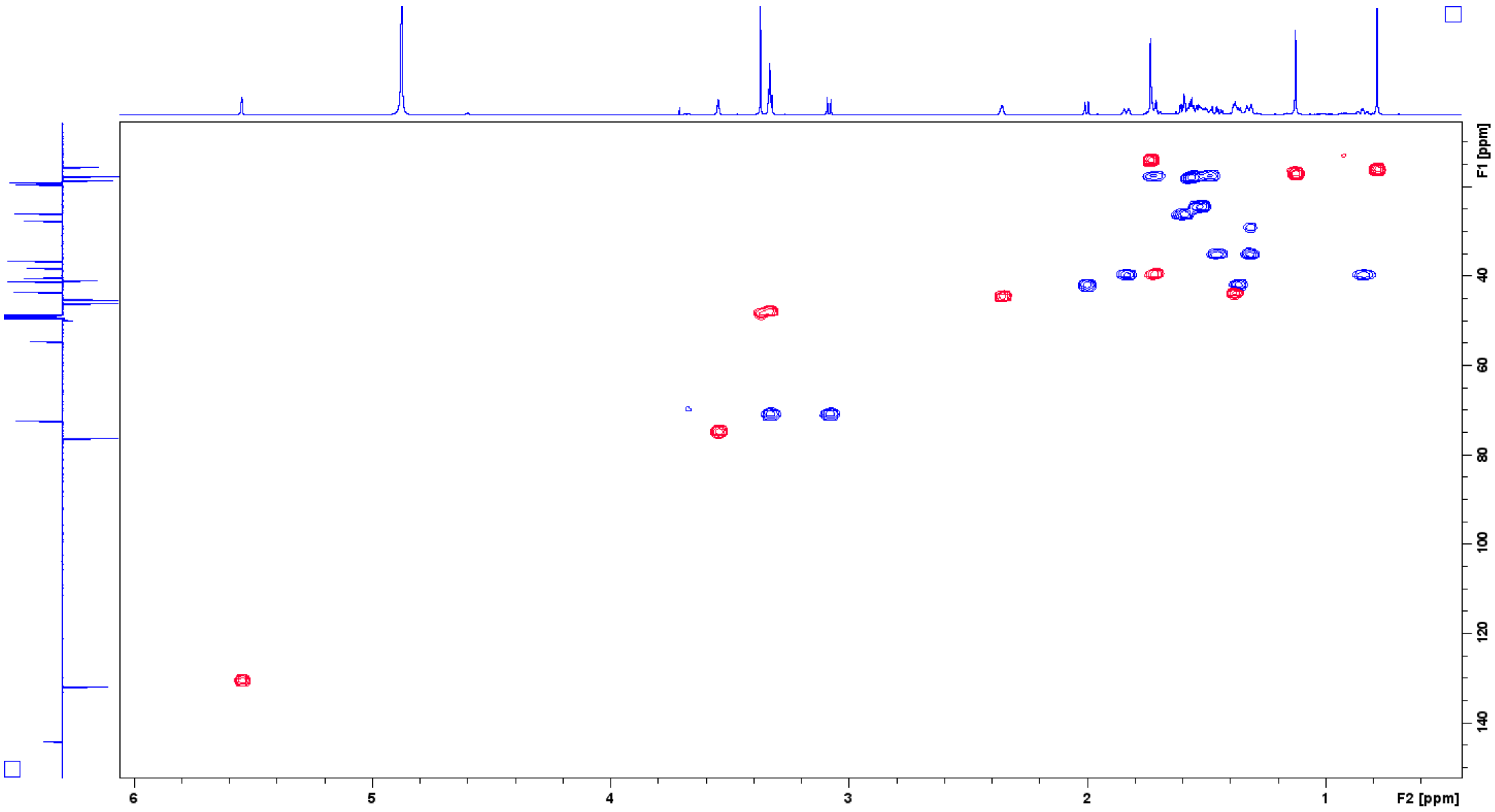

Figure S40. HSQC NMR spectrum of sideridiol (**4**) in CD_3OD (700 MHz).

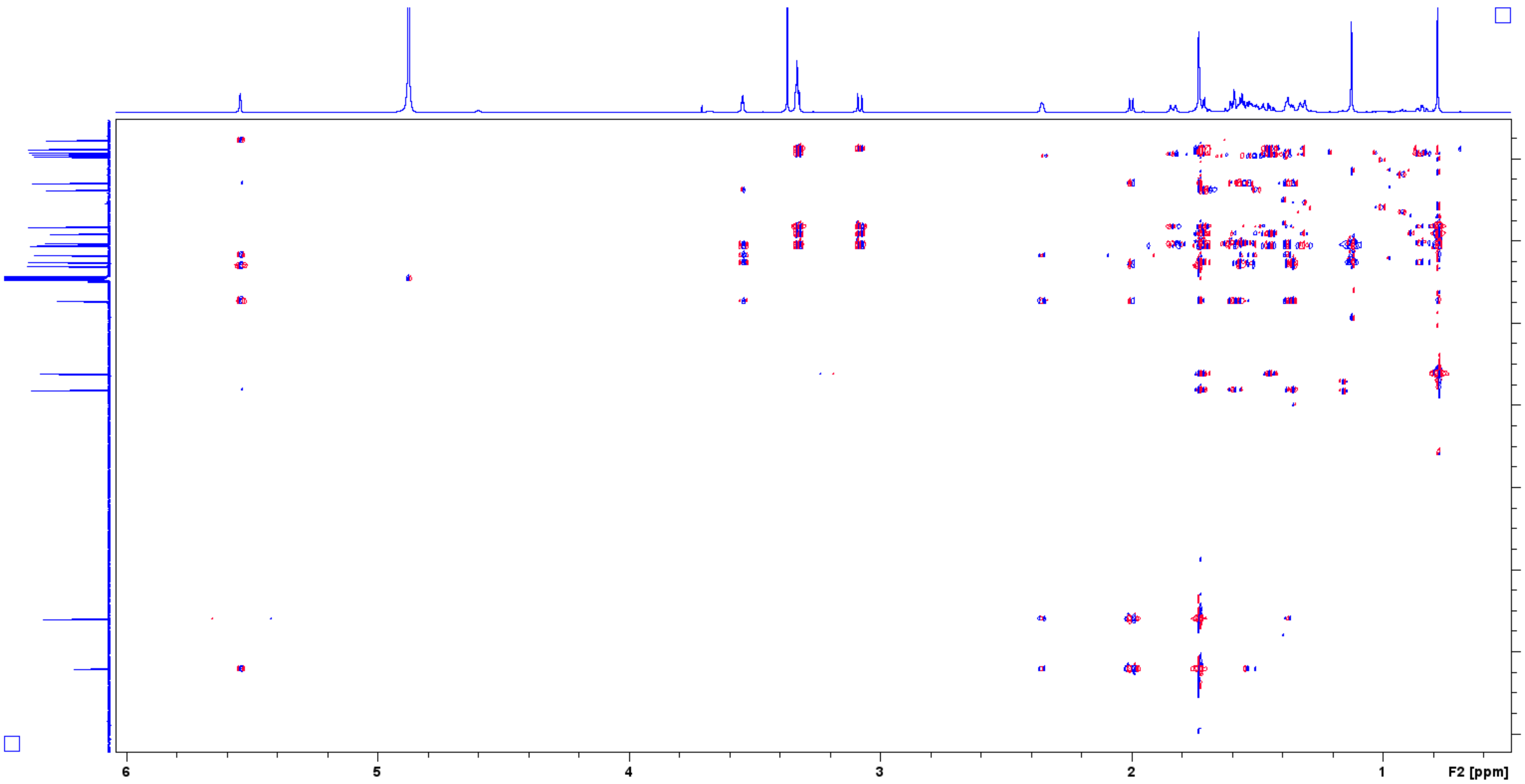

Figure S41. HMBC NMR spectrum of sideridiol (**4**) in CD_3OD (700 MHz).

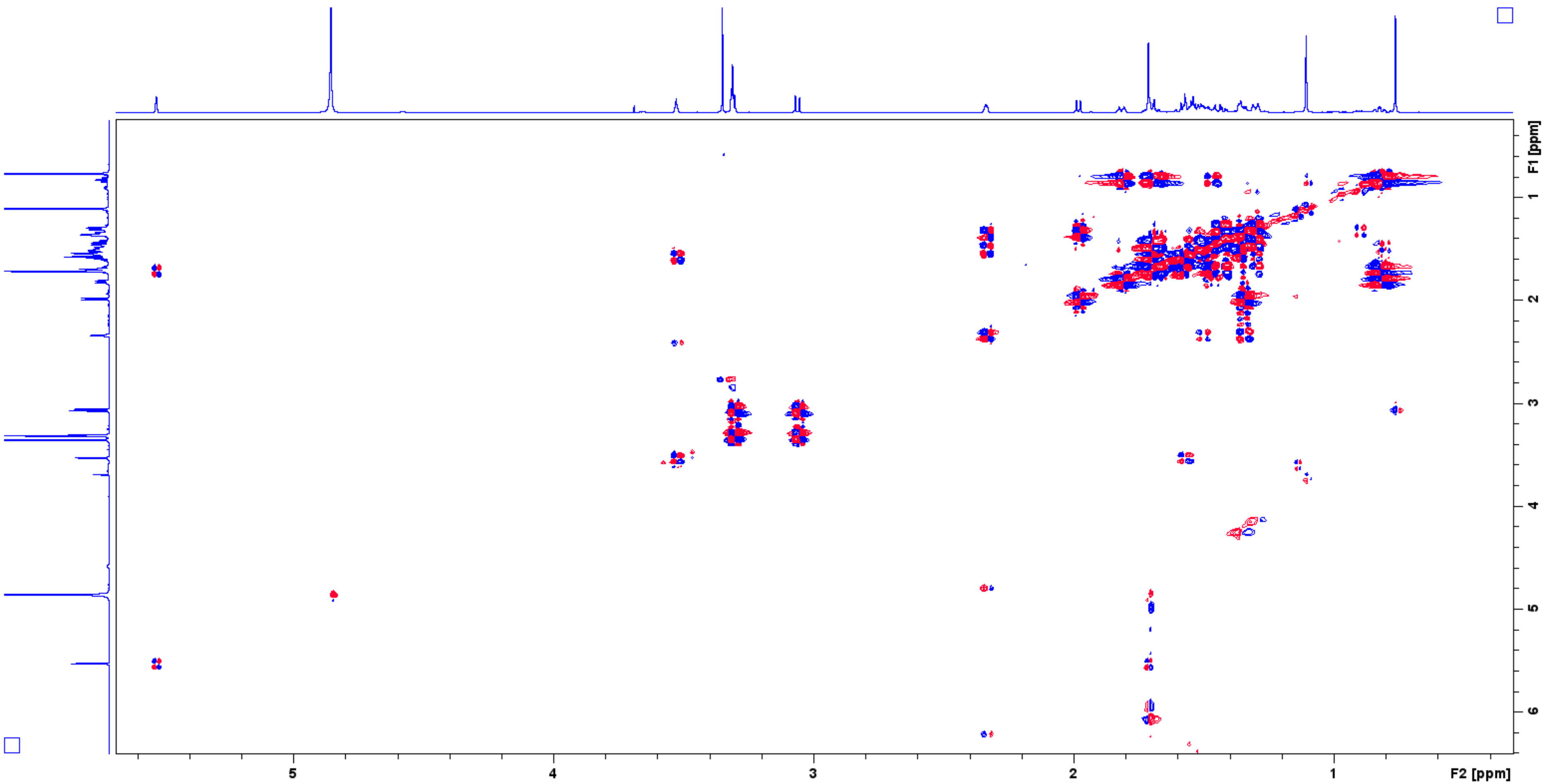

Figure S42. COSY NMR spectrum of sideridiol (**4**) in CD_3OD (700 MHz).

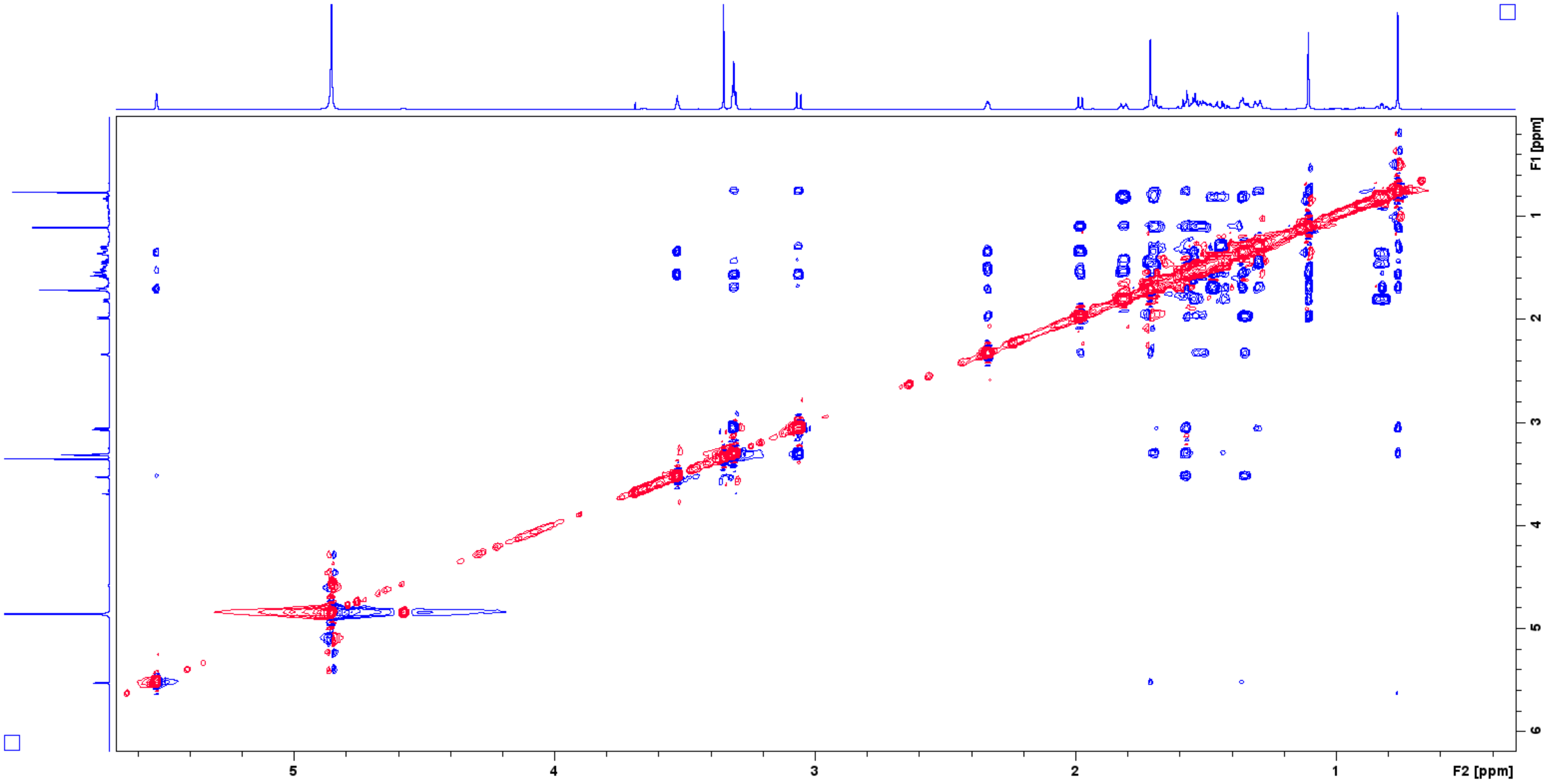

Figure S43. ROESY NMR spectrum of sideridiol (**4**) in CD_3OD (700 MHz).

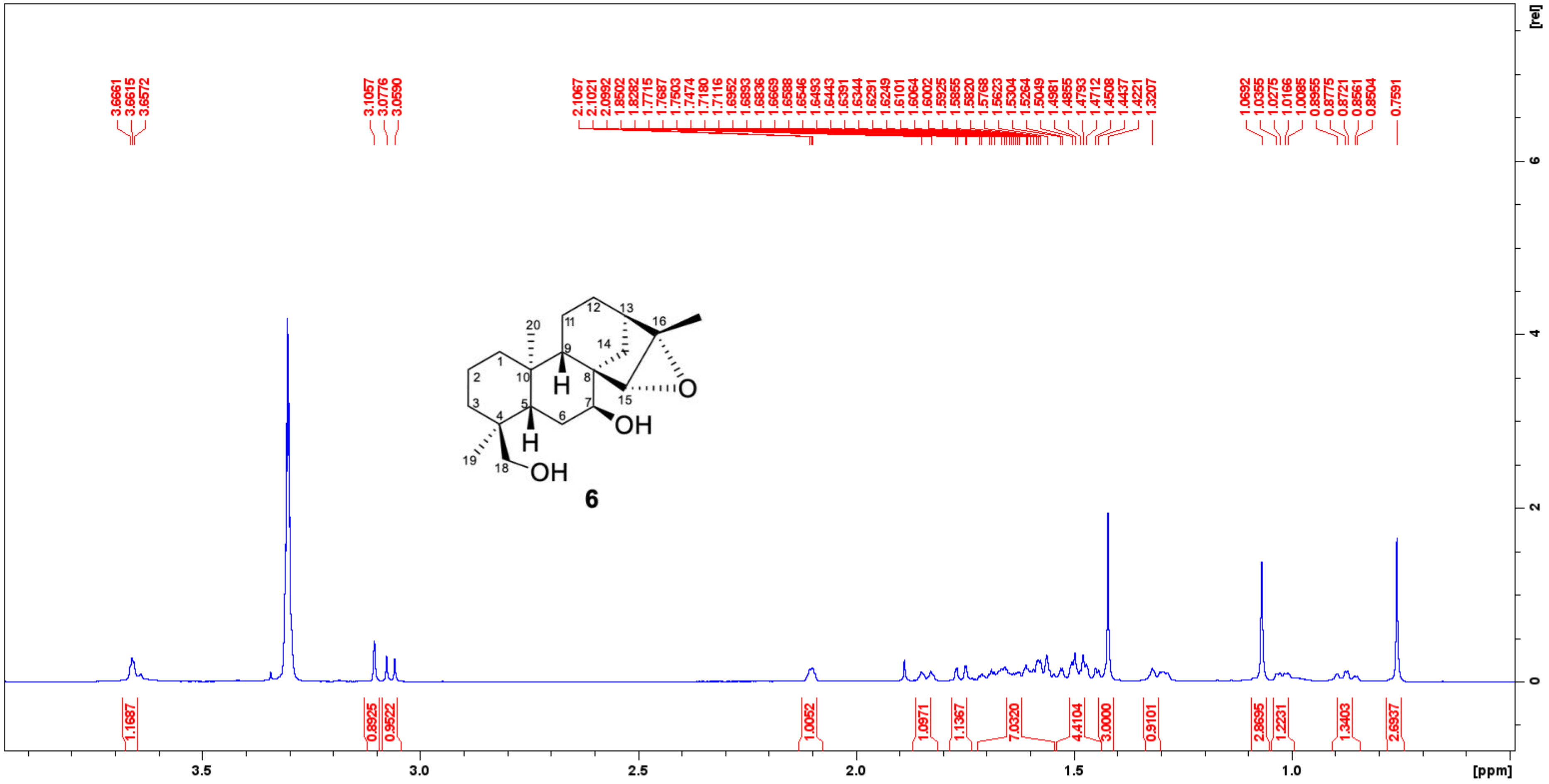

Figure S44. ^{1}H-NMR spectrum of sideroxol (**6**) in CD_3OD (600 MHz).

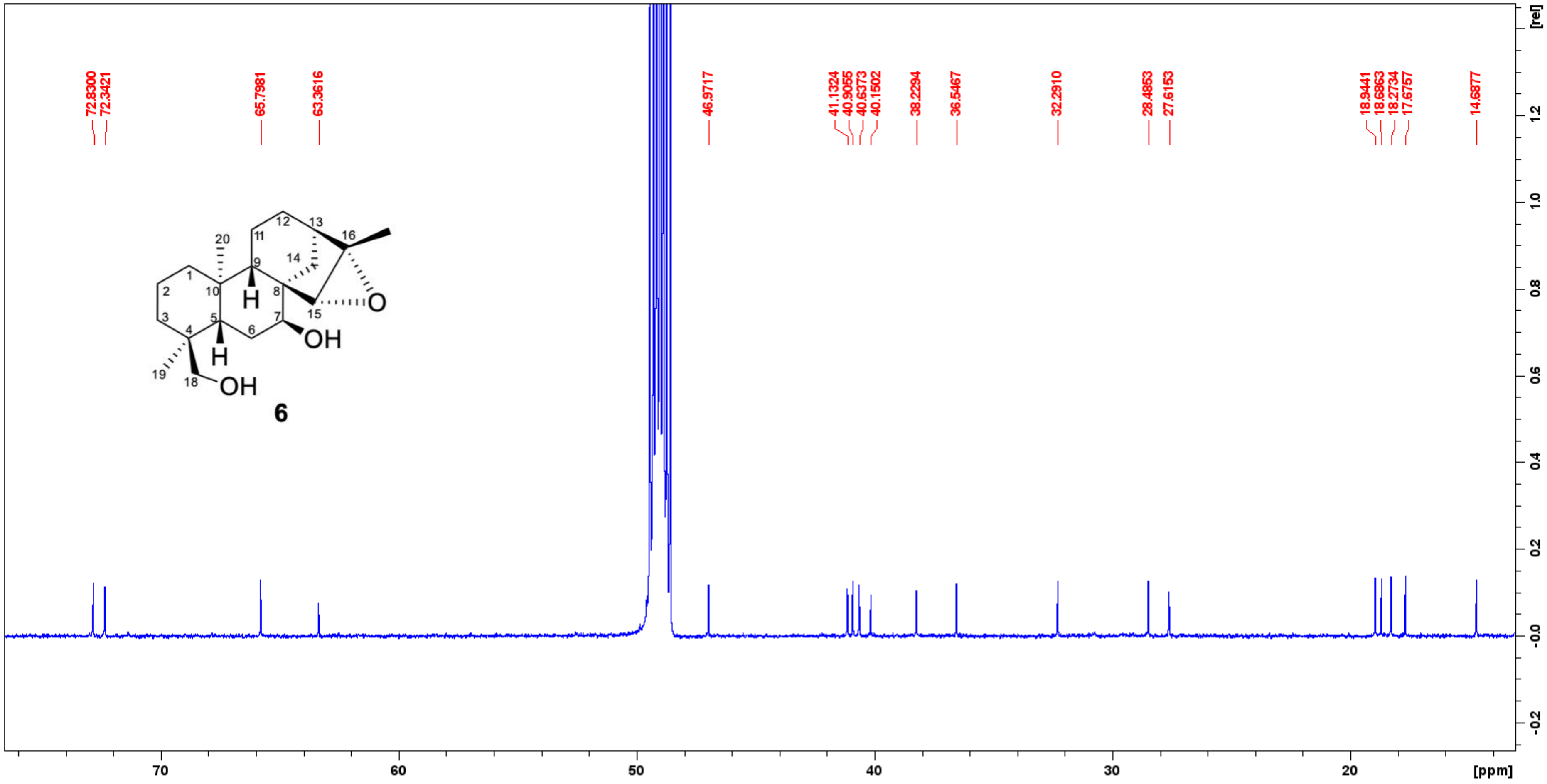

Figure S45. ^{13}C-NMR spectrum of sideroxol (**6**) in CD_3OD (150 MHz).

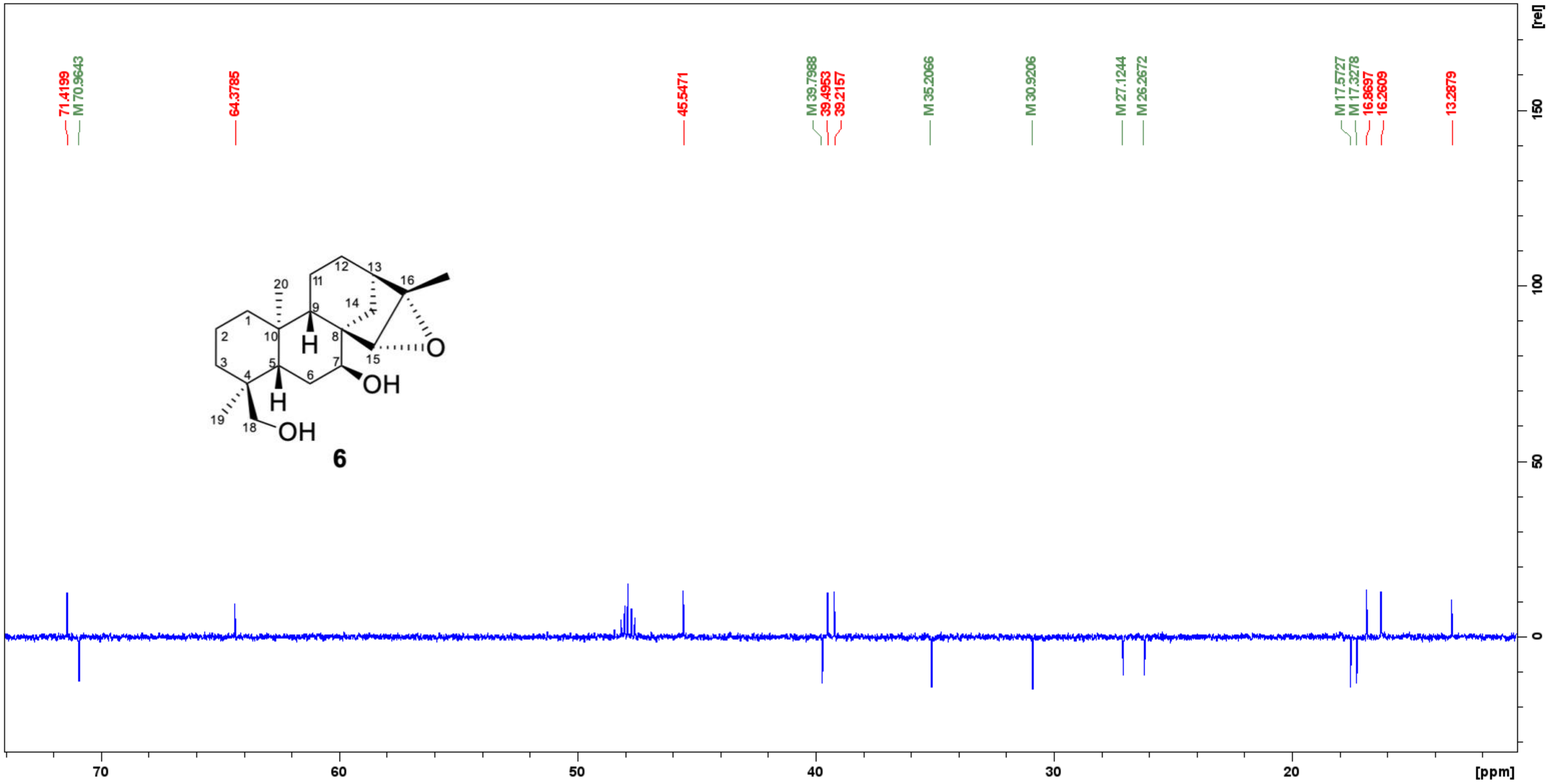

Figure S46. DEPT135-NMR spectrum of sideroxol (**6**) in CD_3OD (150 MHz).

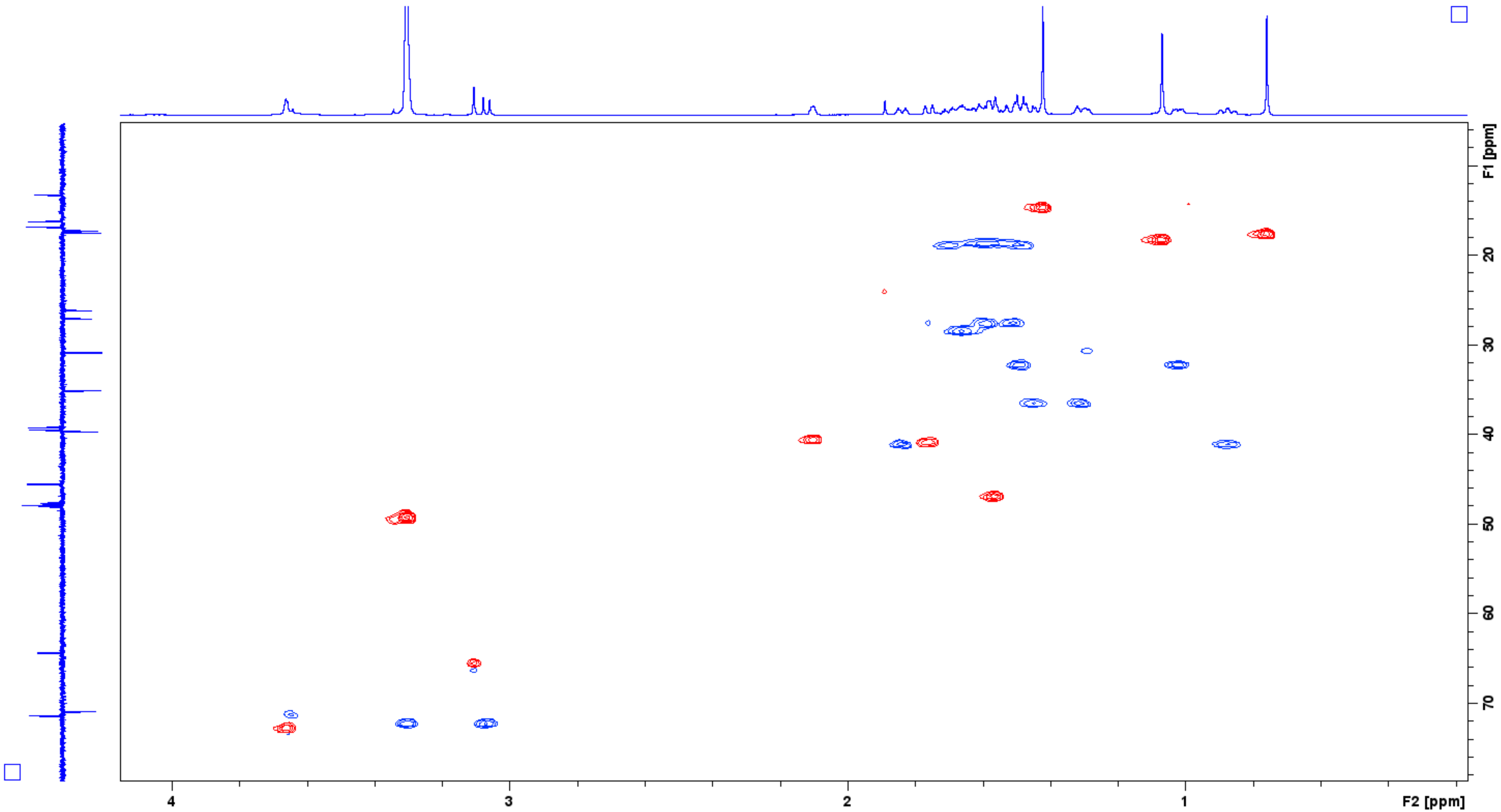

Figure S47. HSQC NMR spectrum of sideroxol (**6**) in CD_3OD (600 MHz).

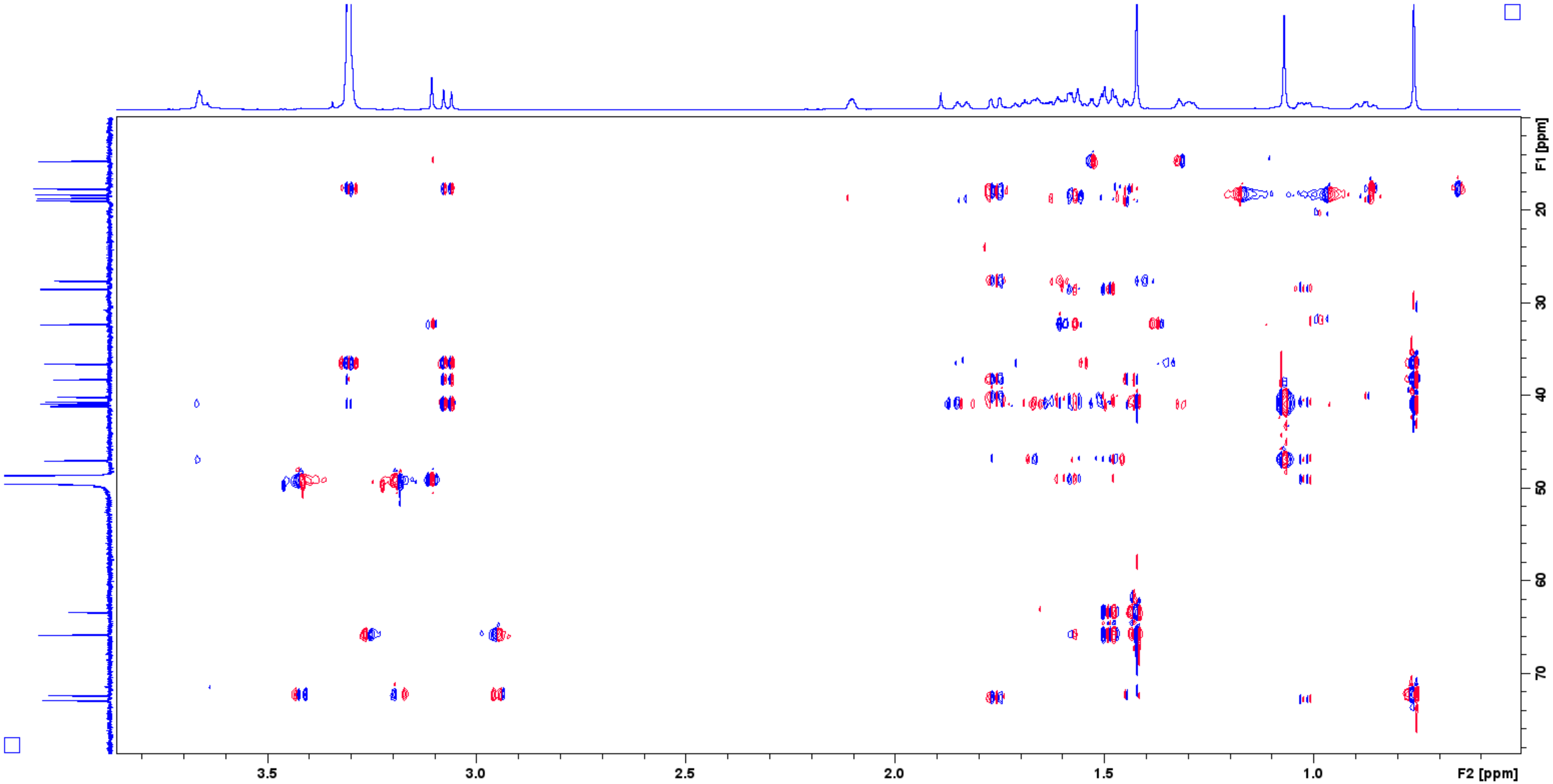

Figure S48. HMBC NMR spectrum of sideroxol (**6**) in CD_3OD (600 MHz).

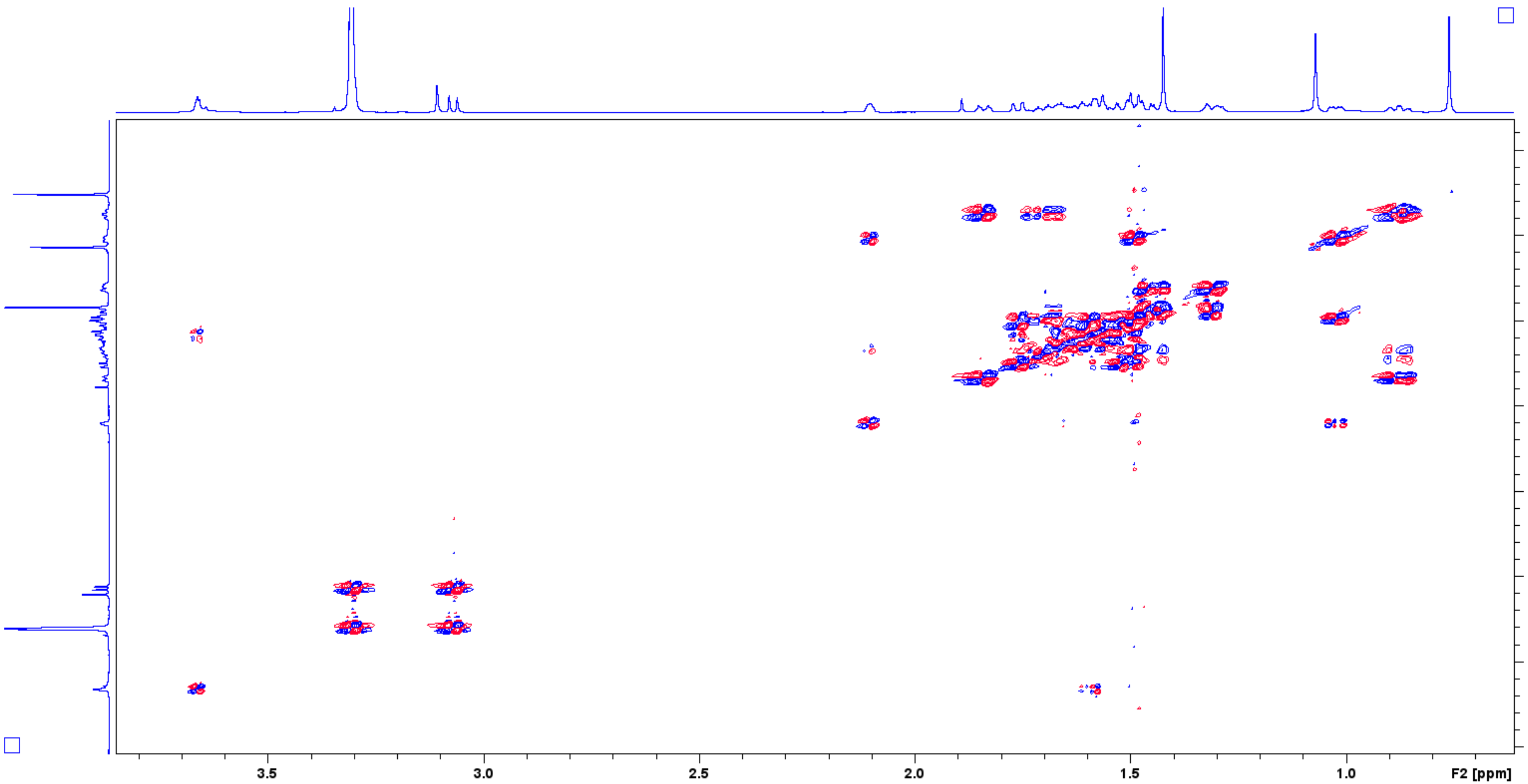

Figure S49. COSY NMR spectrum of sideroxol (**6**) in CD_3OD (600 MHz).

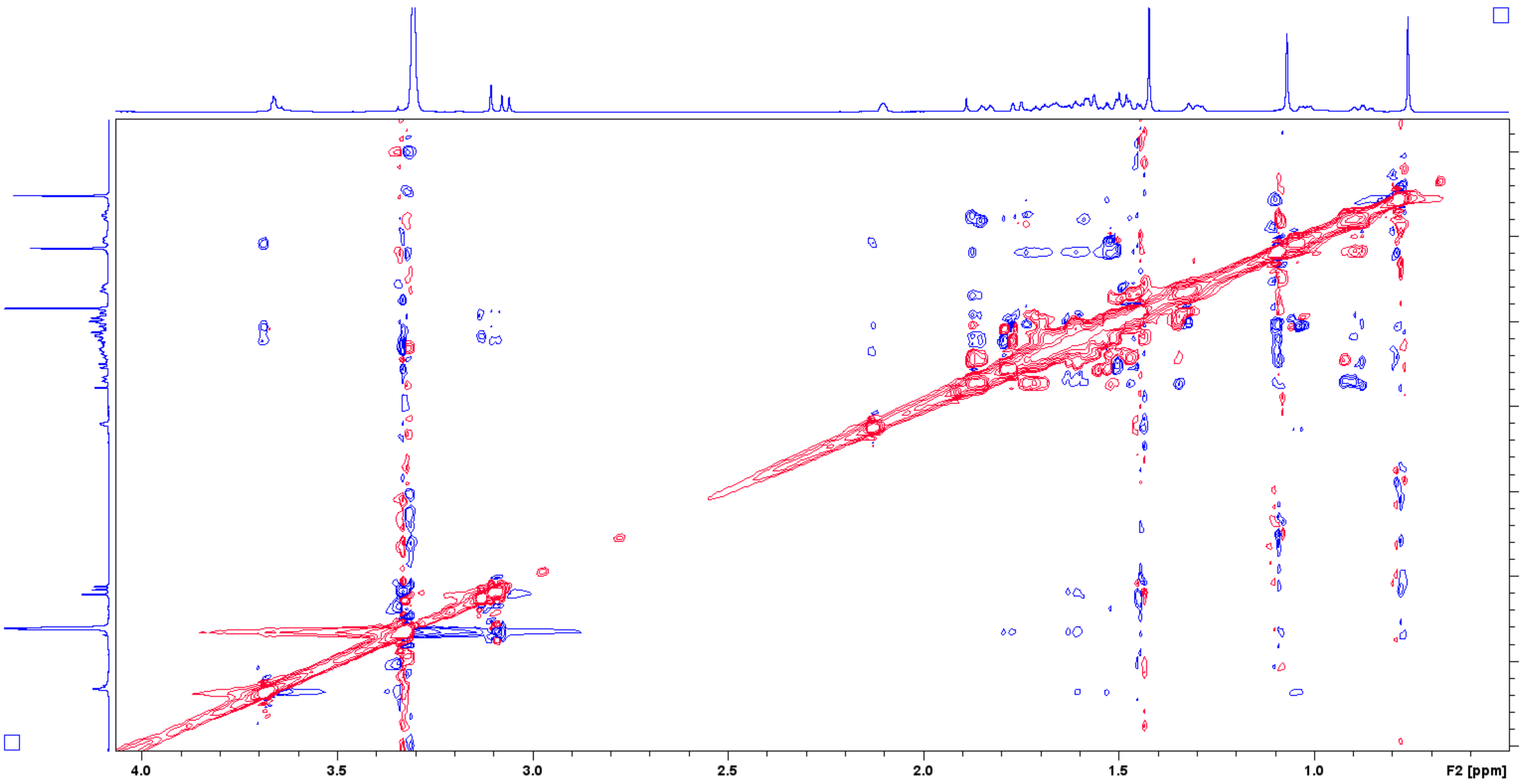

Figure S50. ROESY NMR spectrum of sideroxol (**6**) in CD_3OD (600 MHz).

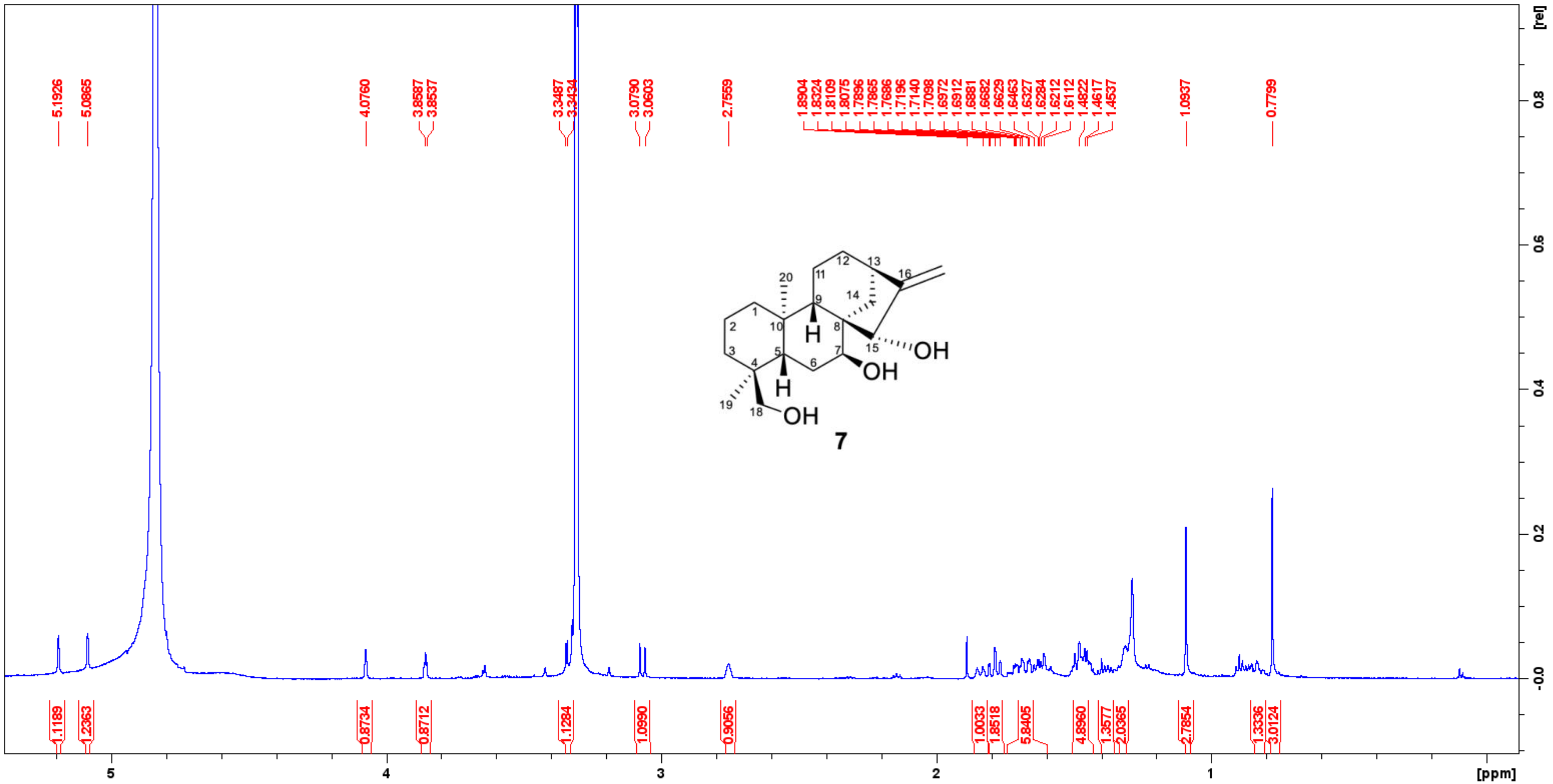

Figure S51. ^{1}H-NMR spectrum of eubotriol (**7**) in CD3OD (600 MHz).

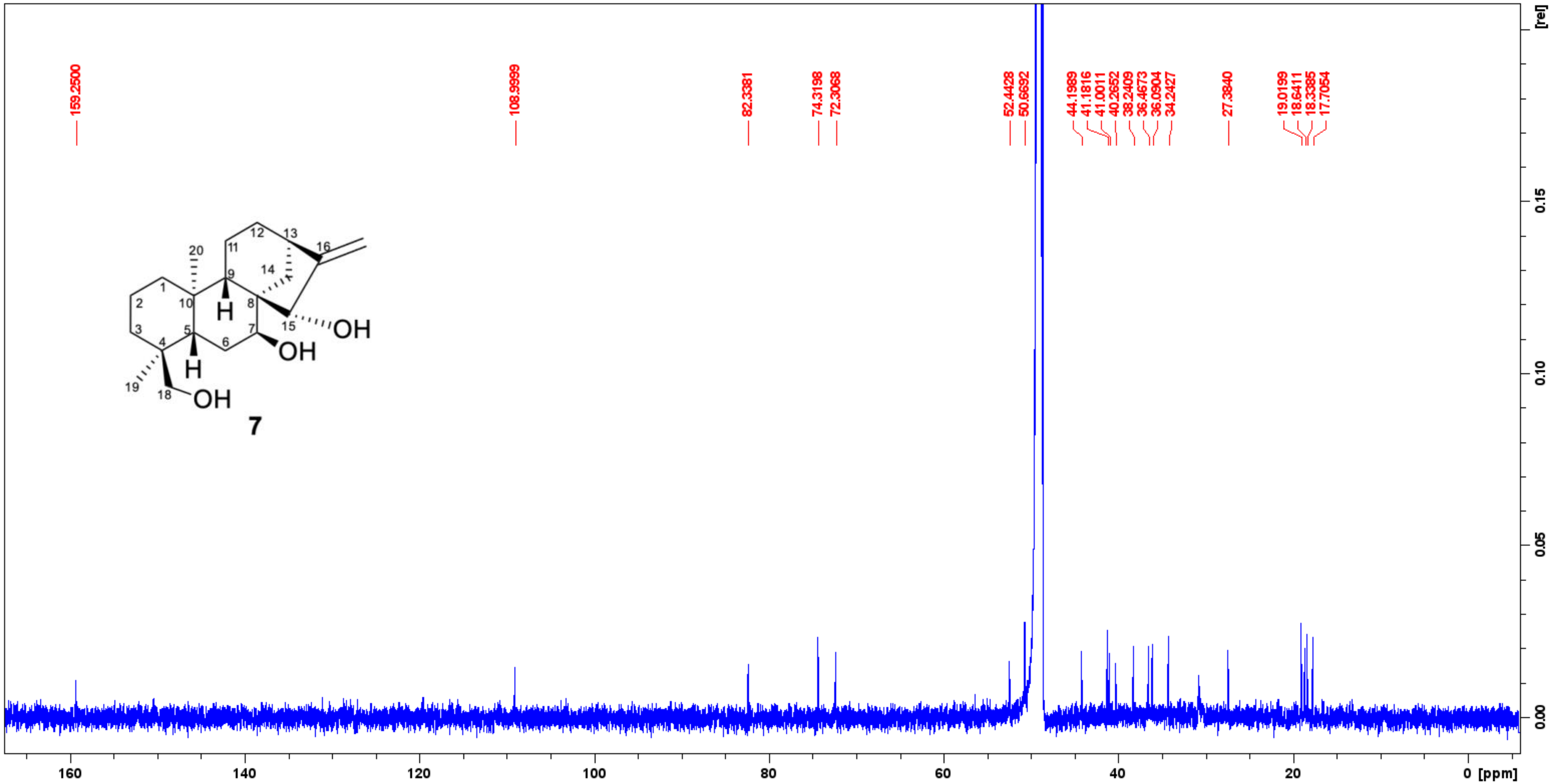

Figure S52. ^{13}C-NMR spectrum of eubotriol (**7**) in CD_3OD (150 MHz).

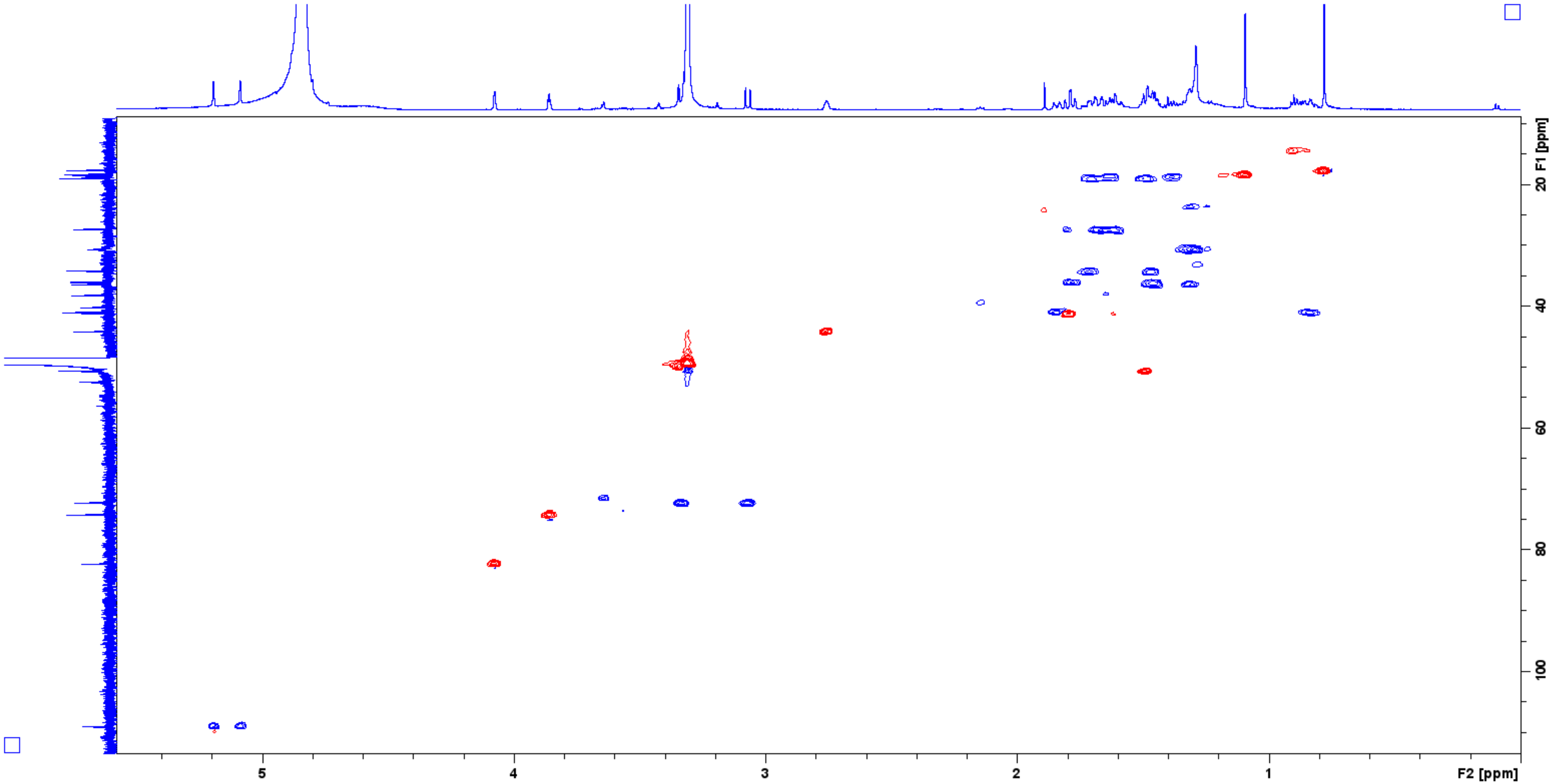

Figure S53. HSQC NMR spectrum of eubotriol (**7**) in CD_3OD (600 MHz).

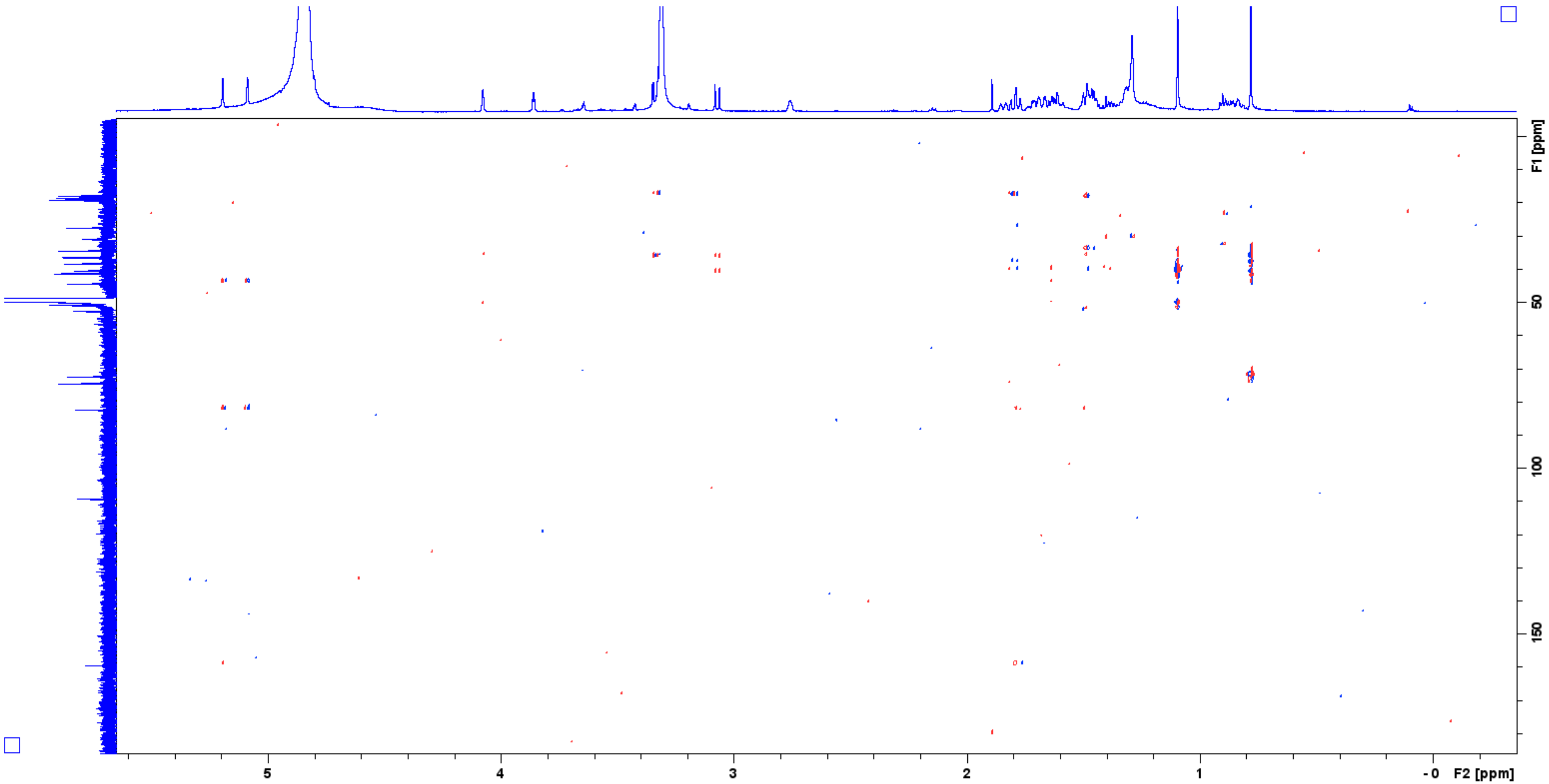

Figure S54. HMBC NMR spectrum of eubotriol (**7**) in CD_3OD (600 MHz).

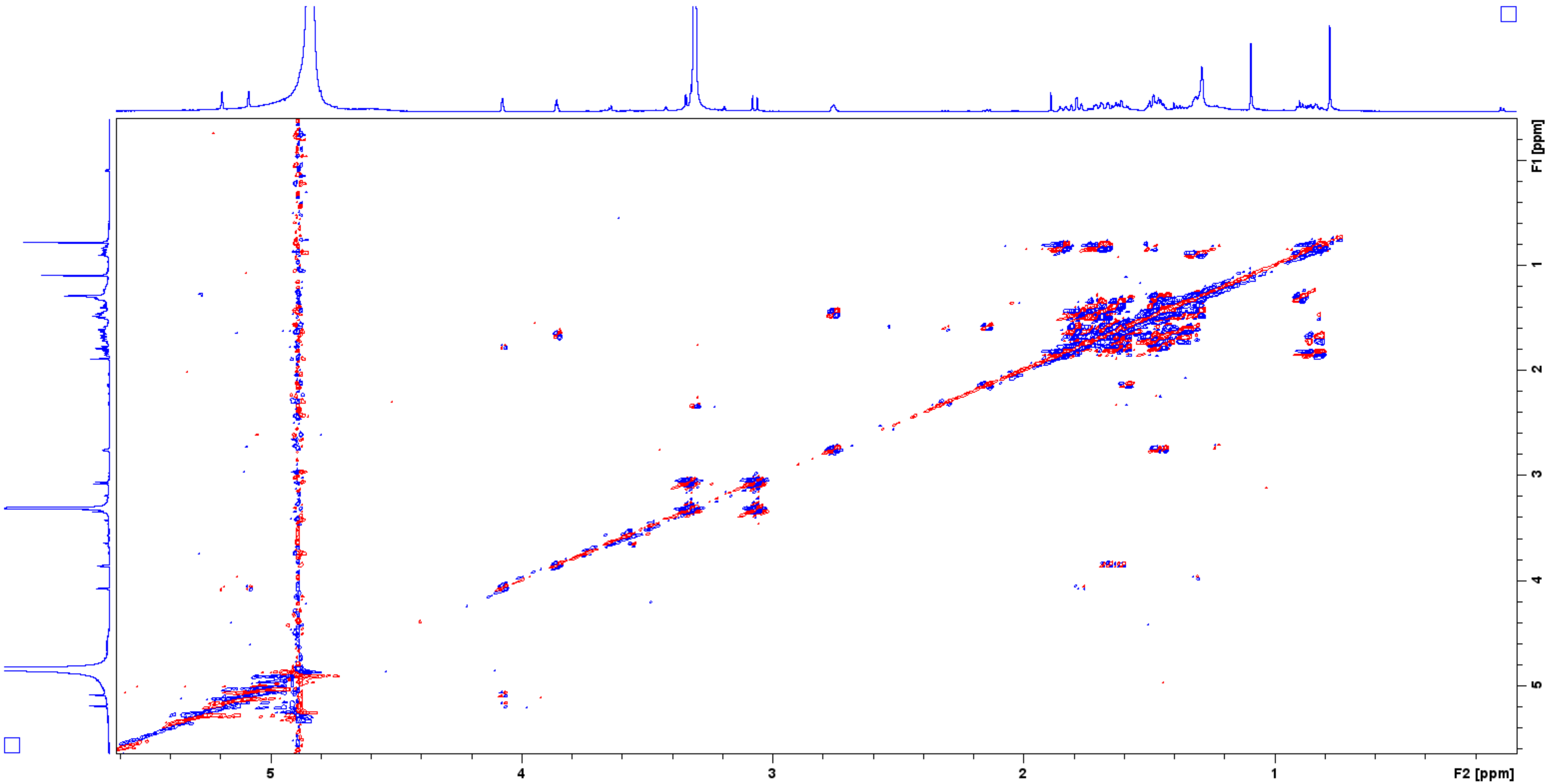

Figure S55. COSY NMR spectrum of eubotriol (**7**) in CD_3OD (600 MHz).

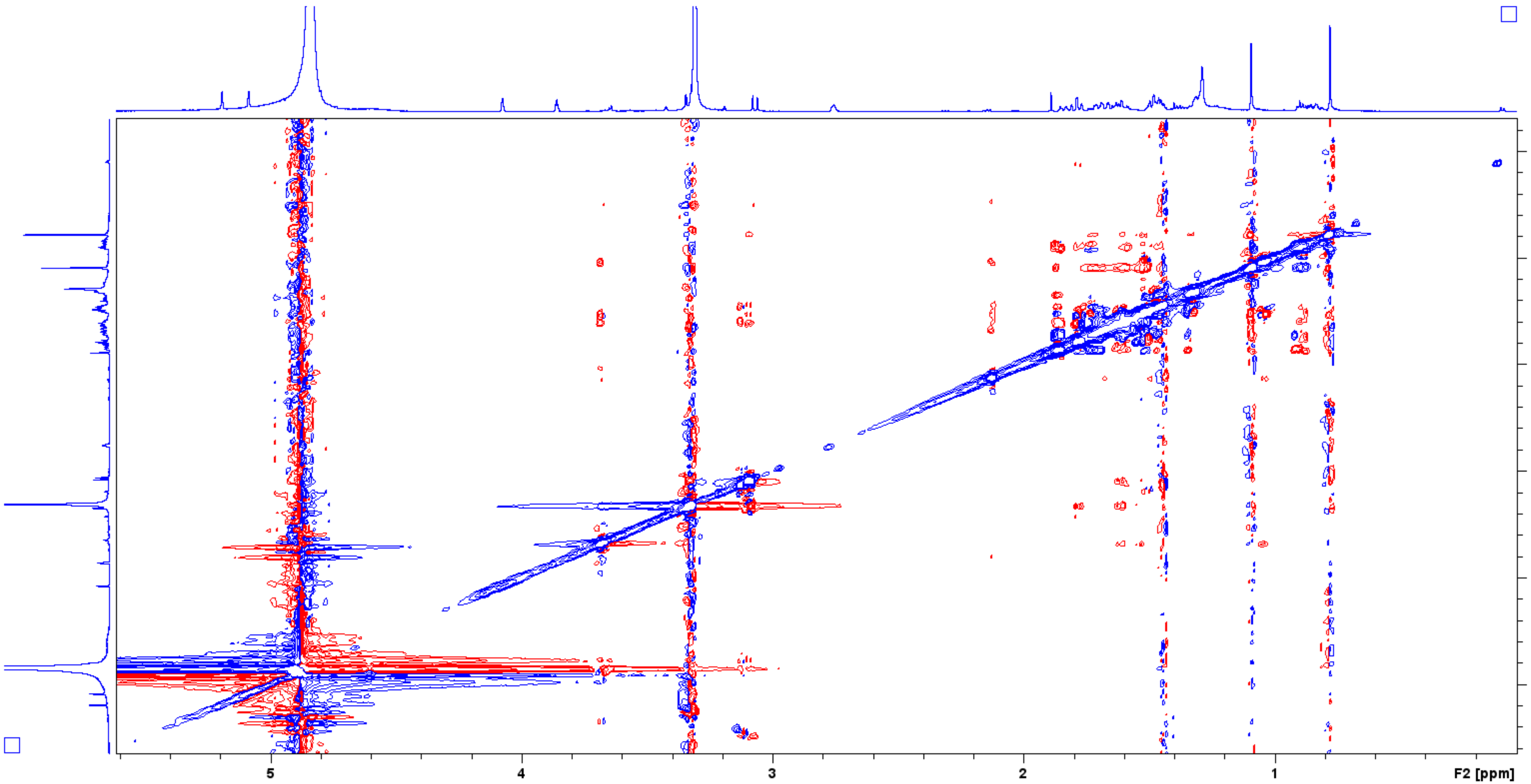

Figure S56. ROESY NMR spectrum of eubotriol (**7**) in CD_3OD (600 MHz).

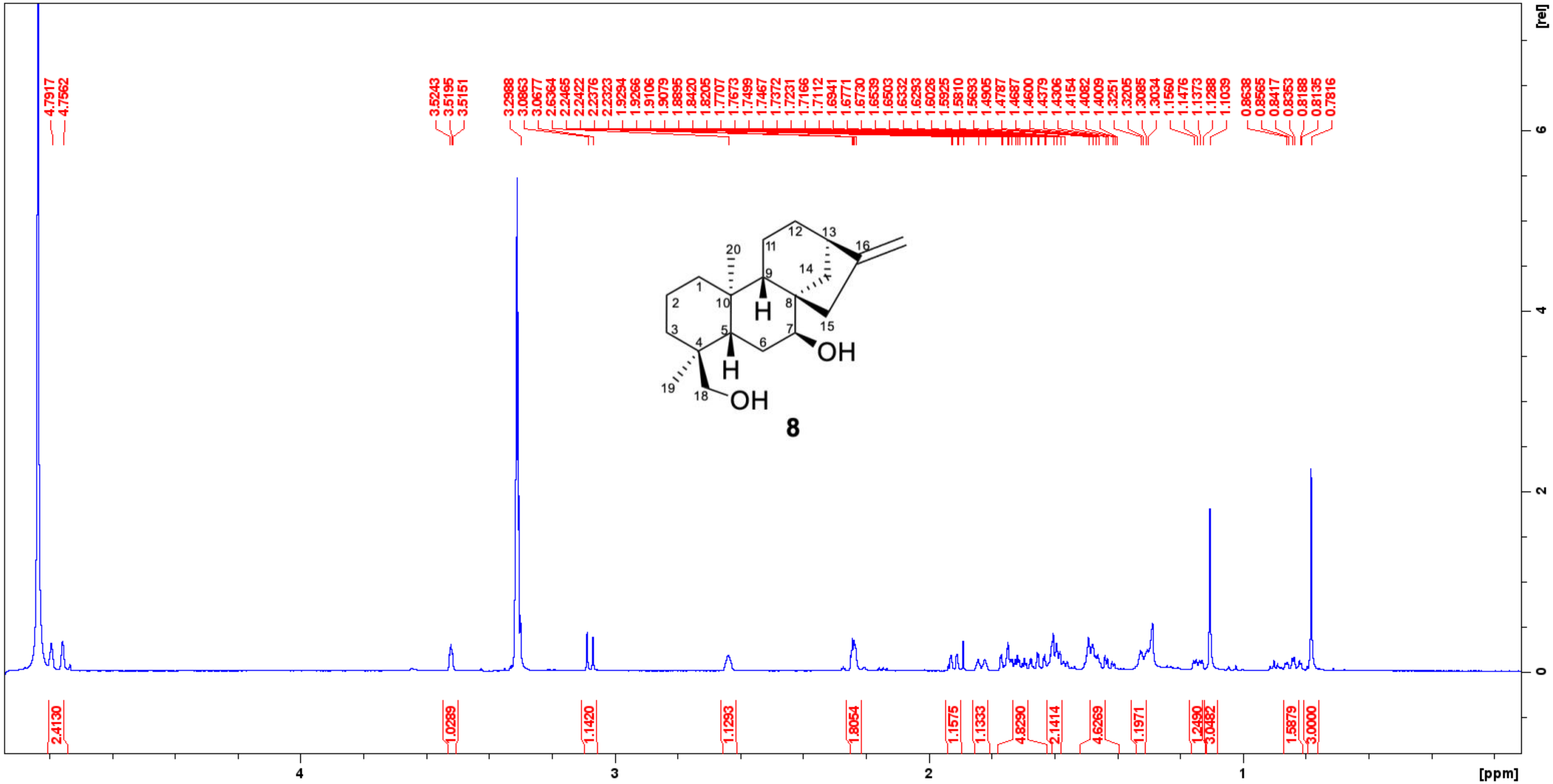

Figure S57. ^{1}H-NMR spectrum of epi-candicandiol (**8**) CD_3OD (600 MHz).

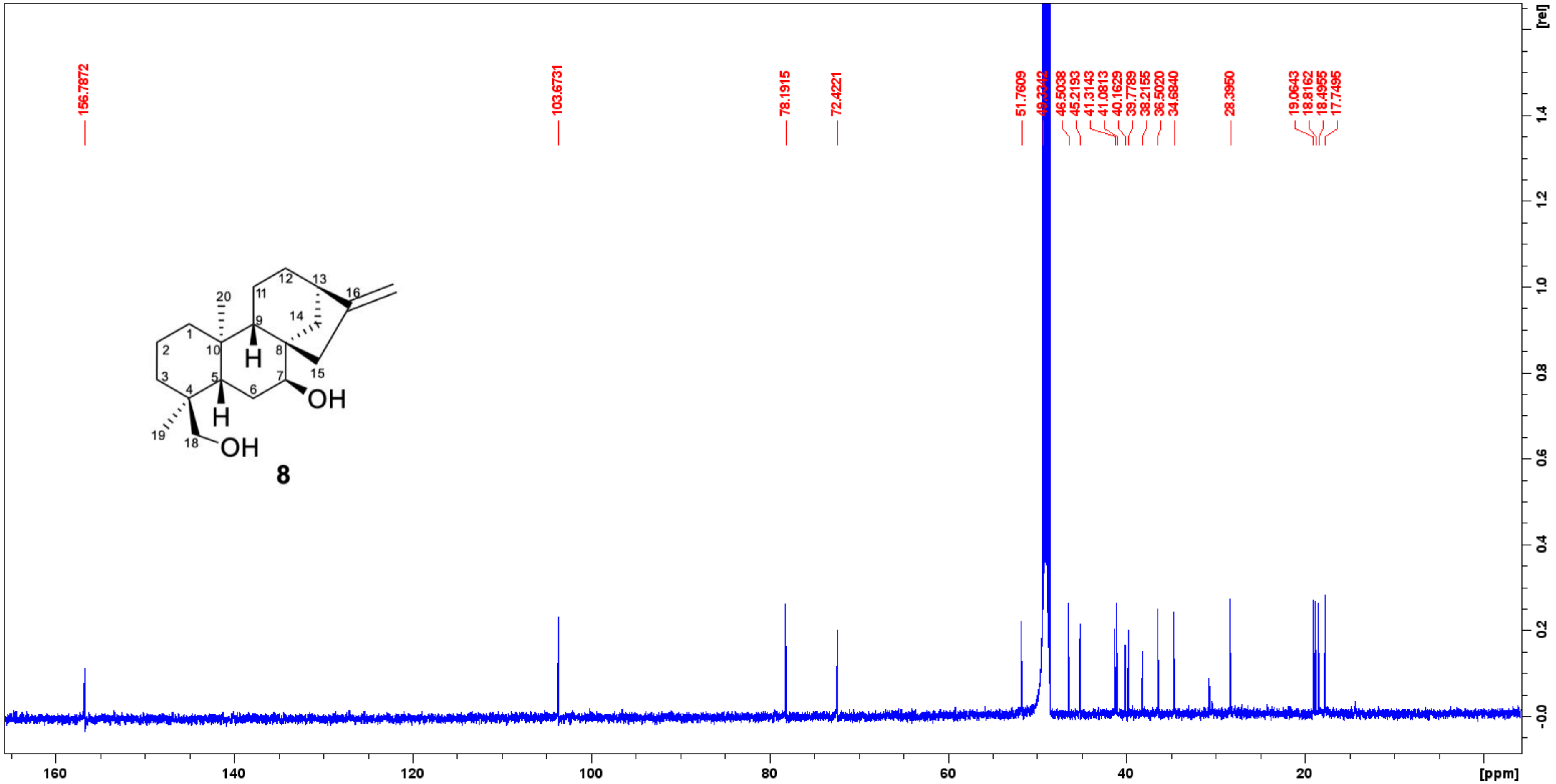

Figure S58. ^{13}C-NMR spectrum of epi-candicandiol (**8**) in CD_3OD (150 MHz).

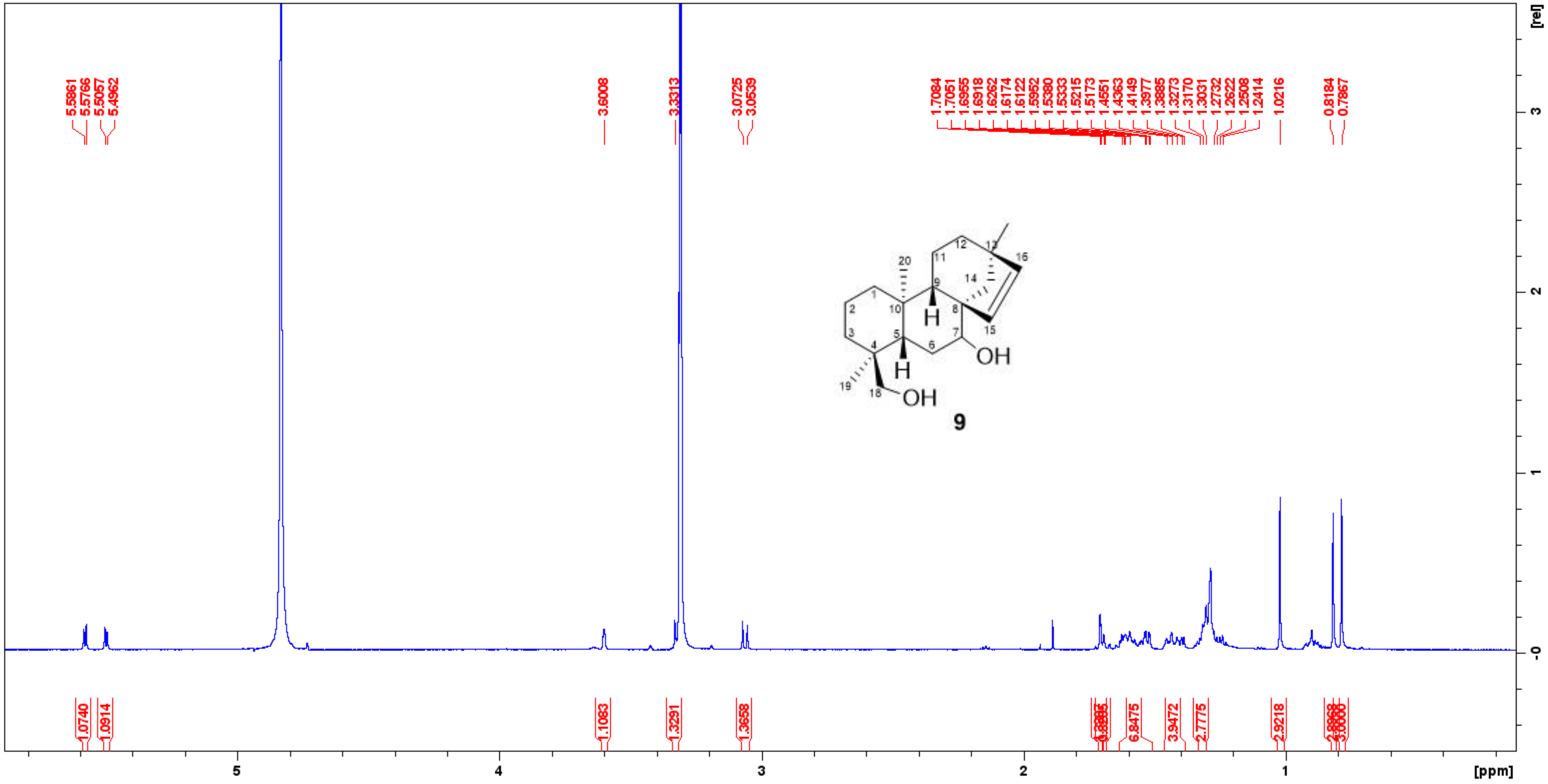

Figure S59. ^{1}H-NMR spectrum of flavovirol (**9**) in CD_3OD (600 MHz).

NMR (CD_3OD, 600 MHz) δ 5.58 (1H, d, *J* = 5.7 Hz, H-15), 5.50 (1H, d, *J* = 5.7 Hz, H-16), 3.60 (1H, s, H-7), 3.30 (1H, H-18a, overlapped by the solvent), 3.06 (1H, d, H-18b, *J* = 11.2 Hz), 1.70-1.69 (1H, m, H-5), 1.62-1.24 (13H, m, H-2, H-3, H-6, H-9, H-11, H-12, H-14), 0.82 (3H, s, CH_3-20), 0.79 (1H, s, CH_3 -19).

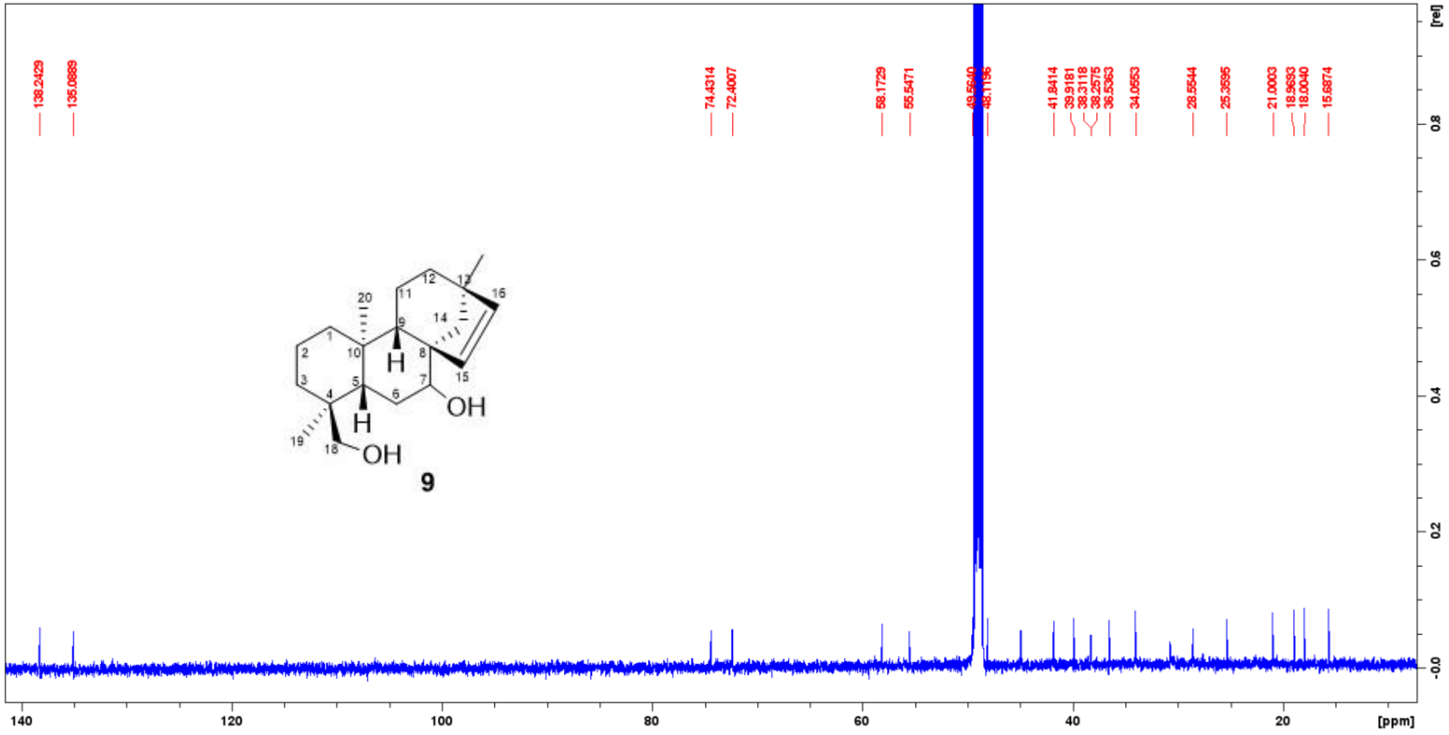

Figure S60. ^{13}C-NMR spectrum of flavovirol (**9**) in CD_3OD (150 MHz).

^{13}C NMR (CDCl3, 150 MHz) δ 138.24 (CH, C-15), 135.09 (CH, C-16), 74.43 (CH, C-7), 72.40 (CH2, C-18), 58.17 (CH2, C-14), 55.54 (C, C-8), 48.11 (CH, C-9), 41.84 (CH, C-5), 38.31(C, C-4), 38.25 (C, C-10), 18.96 (CH3, C-17), 18.00 (CH_3, C-20), 15.68 (CH_3, C-19).

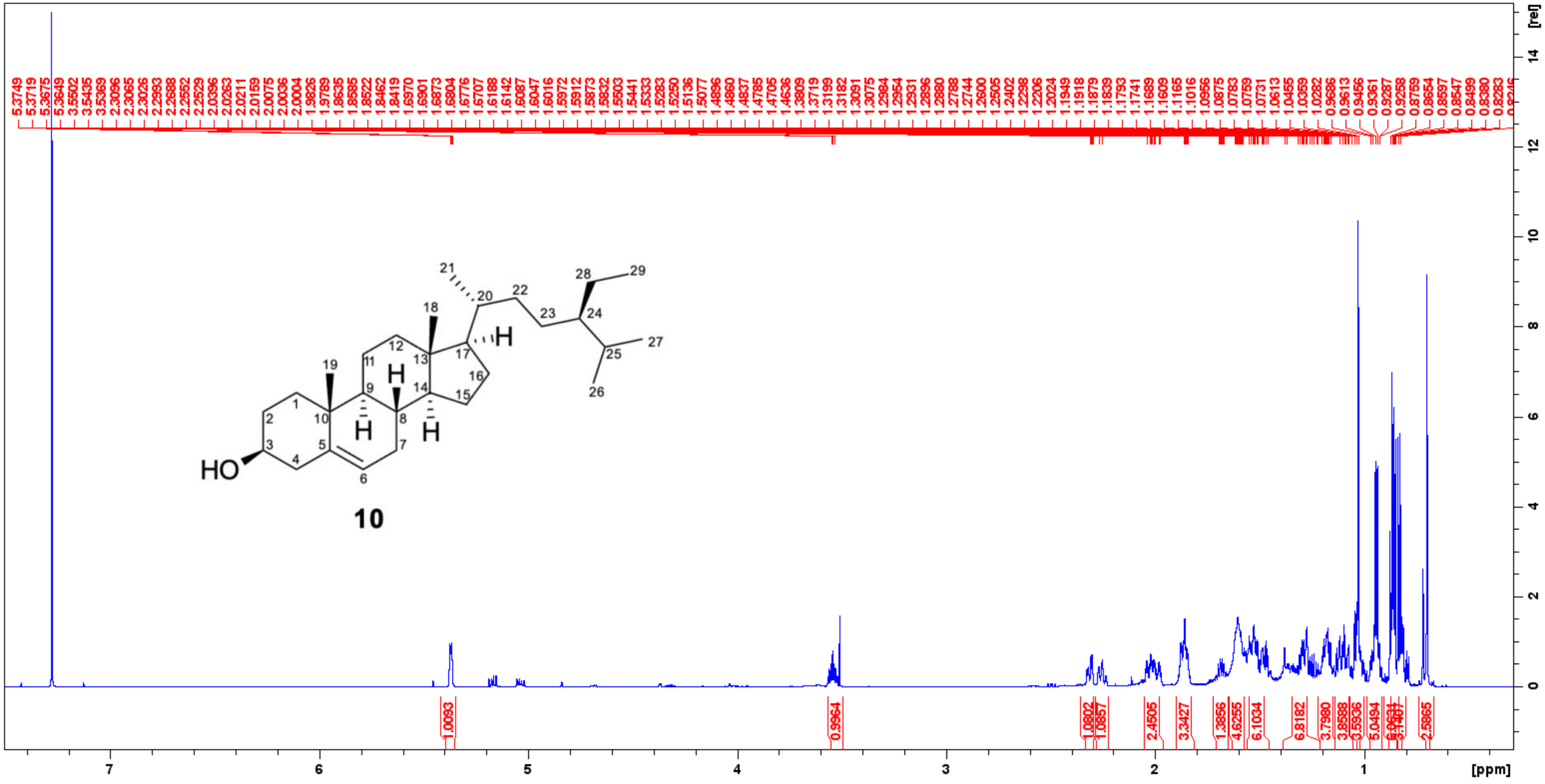

Figure S61. ^{1}H-NMR spectrum of β-sitosterol (**10**) in $CDCl_3$ (700 MHz)

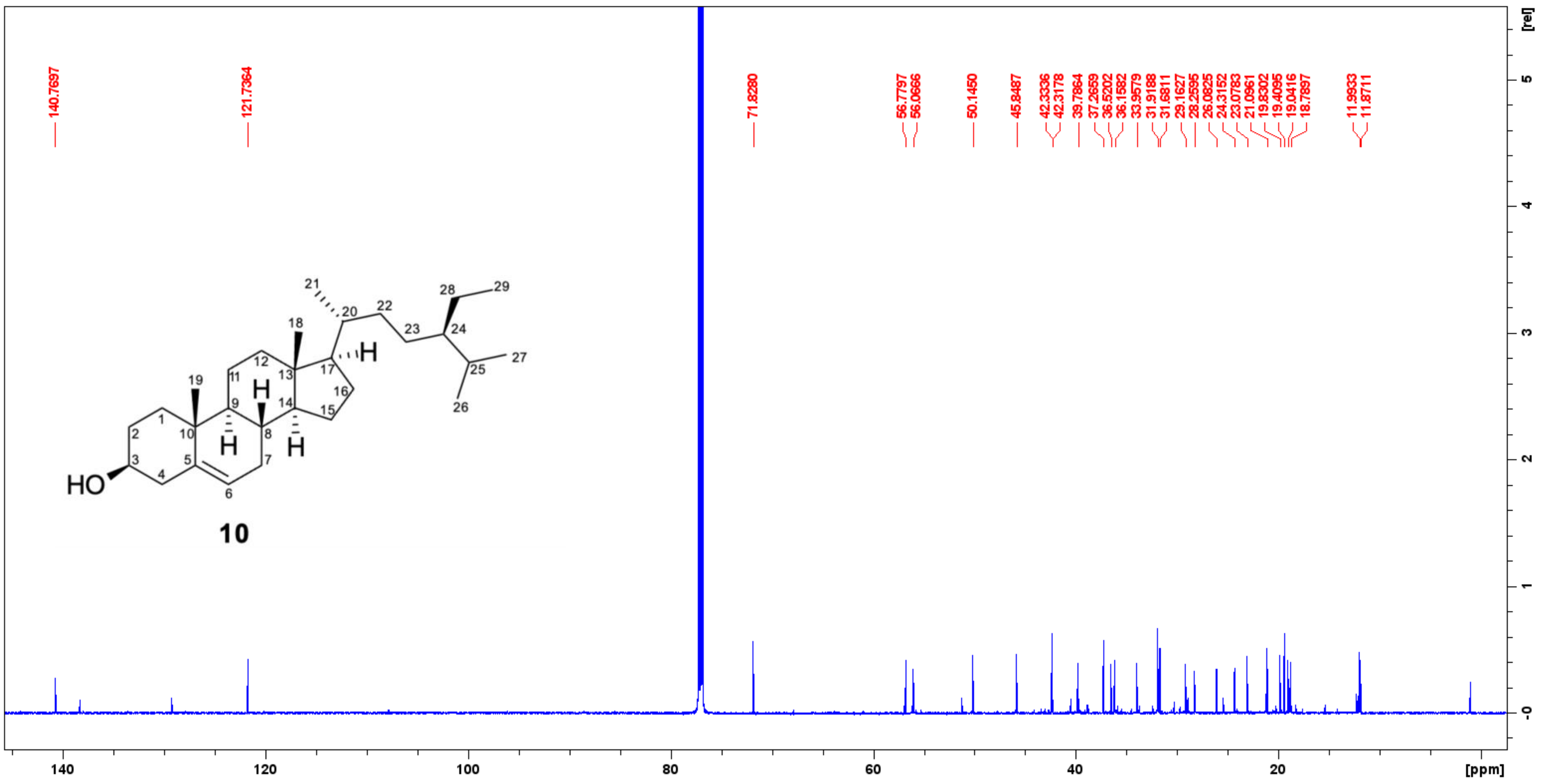

Figure S62. ^{13}C-NMR spectrum of β-sitosterol (**10**) in $CDCl_3$ (176 MHz)

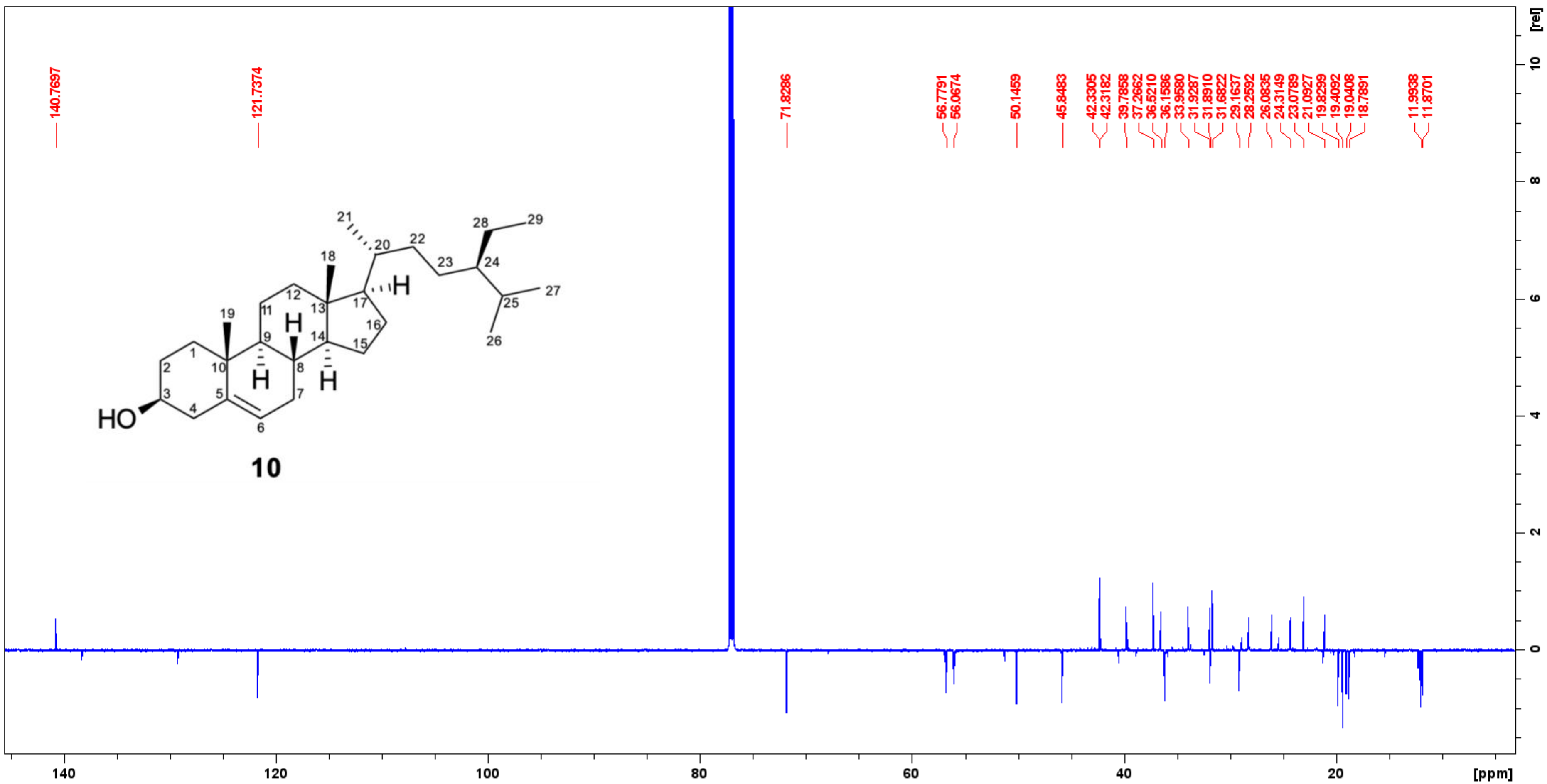

Figure S63. APT NMR spectrum of β-sitosterol (**10**) in $CDCl_3$, (176 MHz)

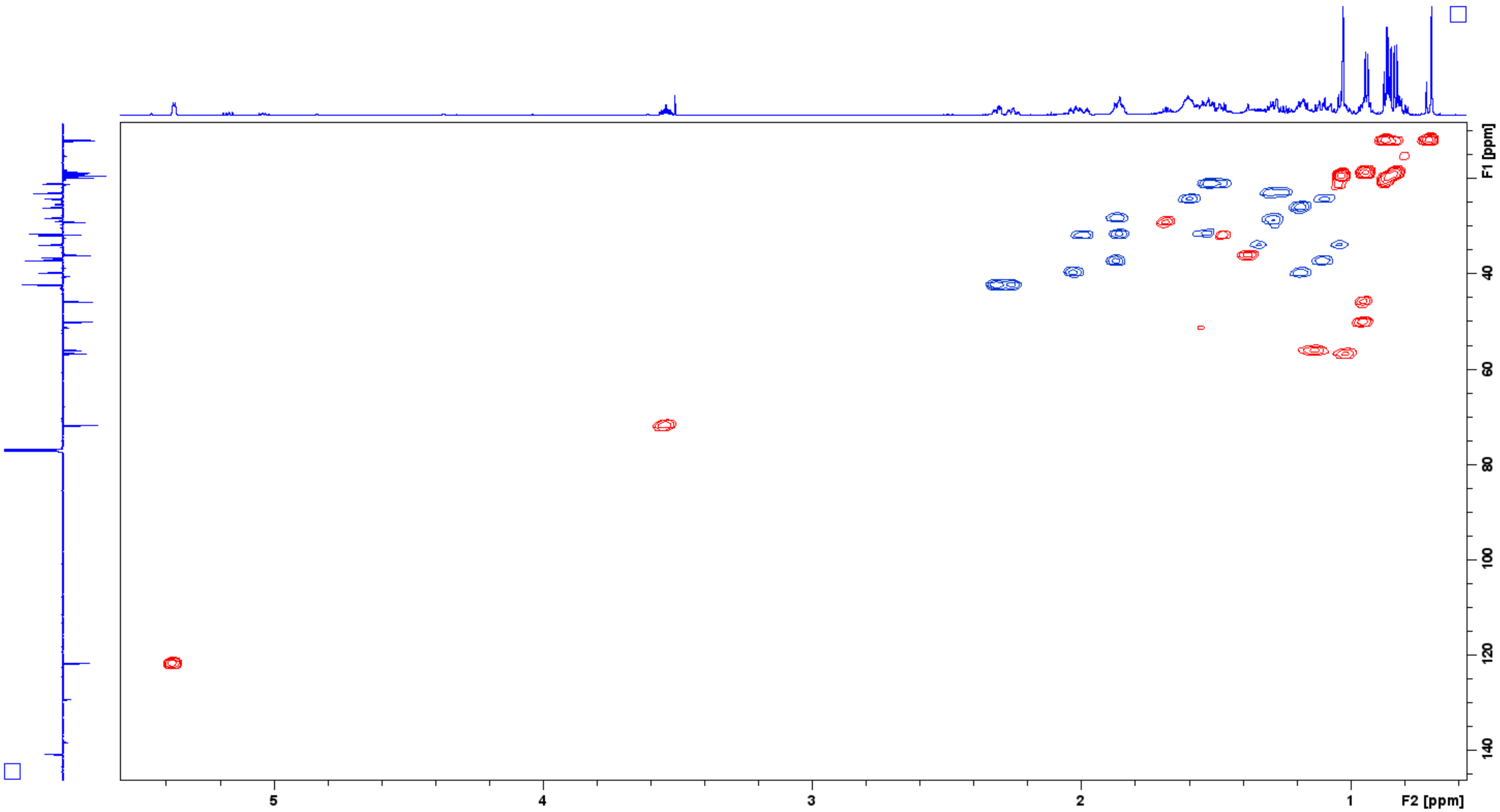

Figure S64. HSQC NMR spectrum of β-sitosterol (**10**) in $CDCl_3$ (700 MHz)

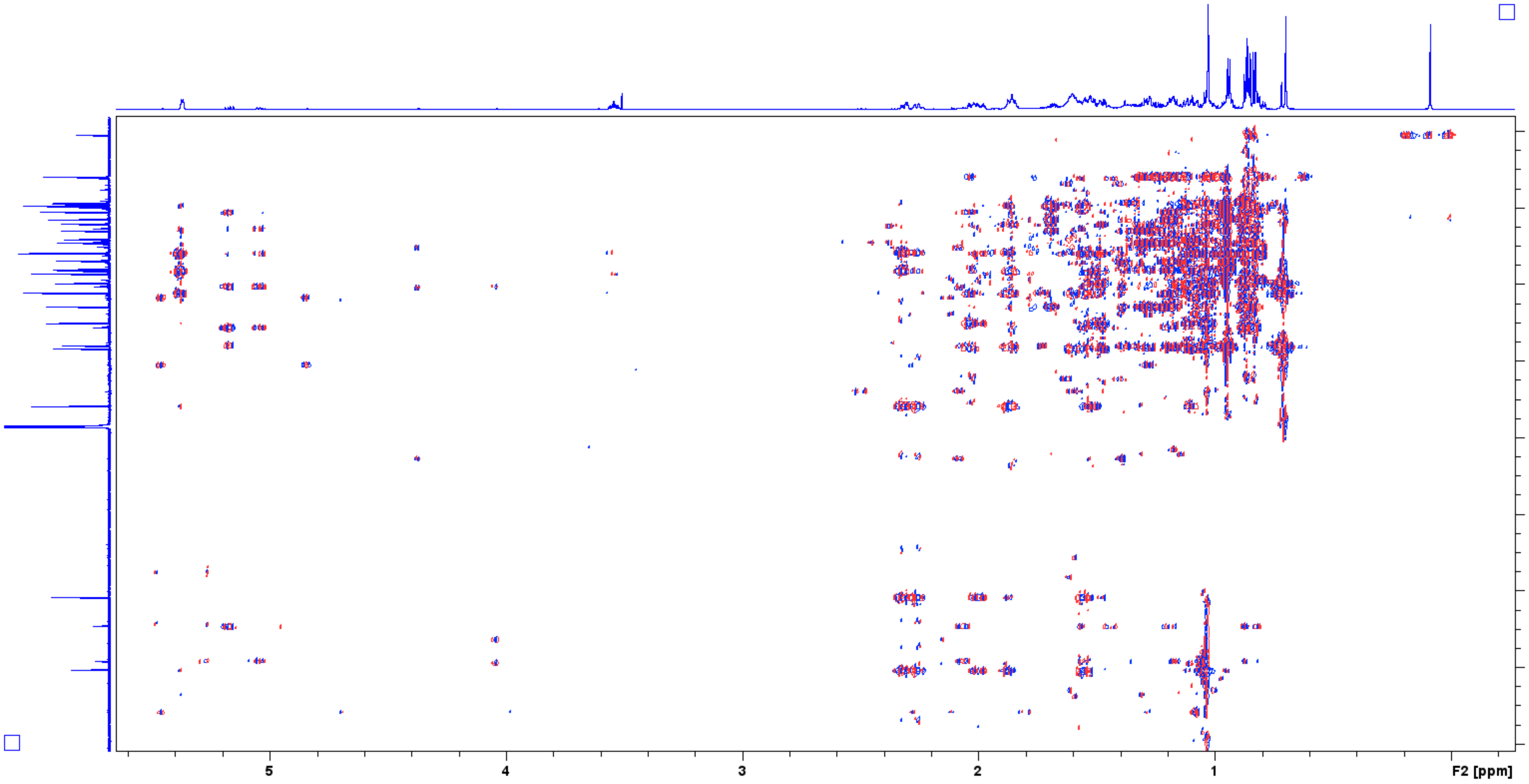

Figure S65. HMBC NMR spectrum of β-sitosterol (**10**) in $CDCl_3$ (700 MHz)

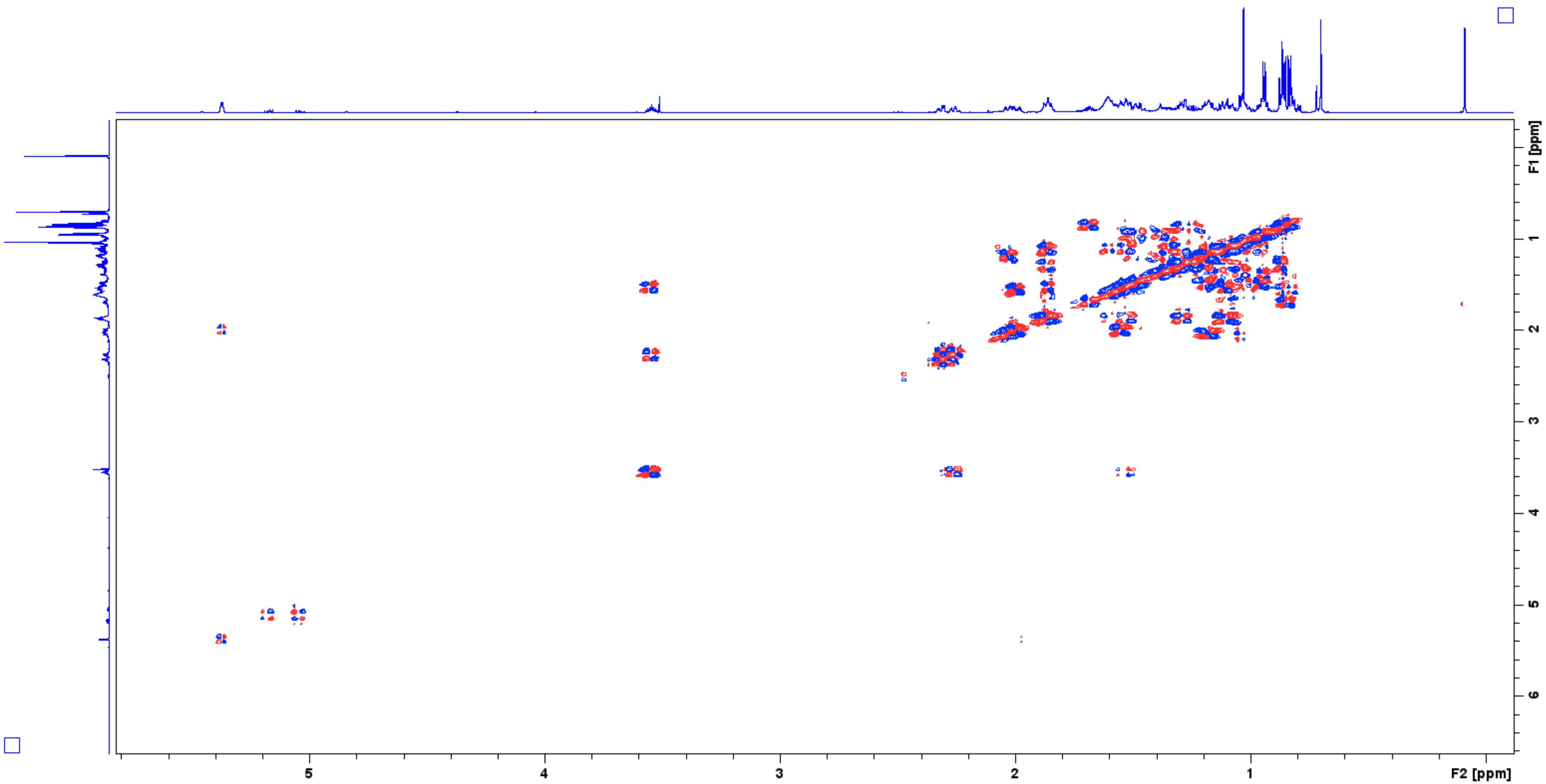

Figure S66. COSY NMR spectrum of β-sitosterol (**10**) in $CDCl_3$ (700 MHz)

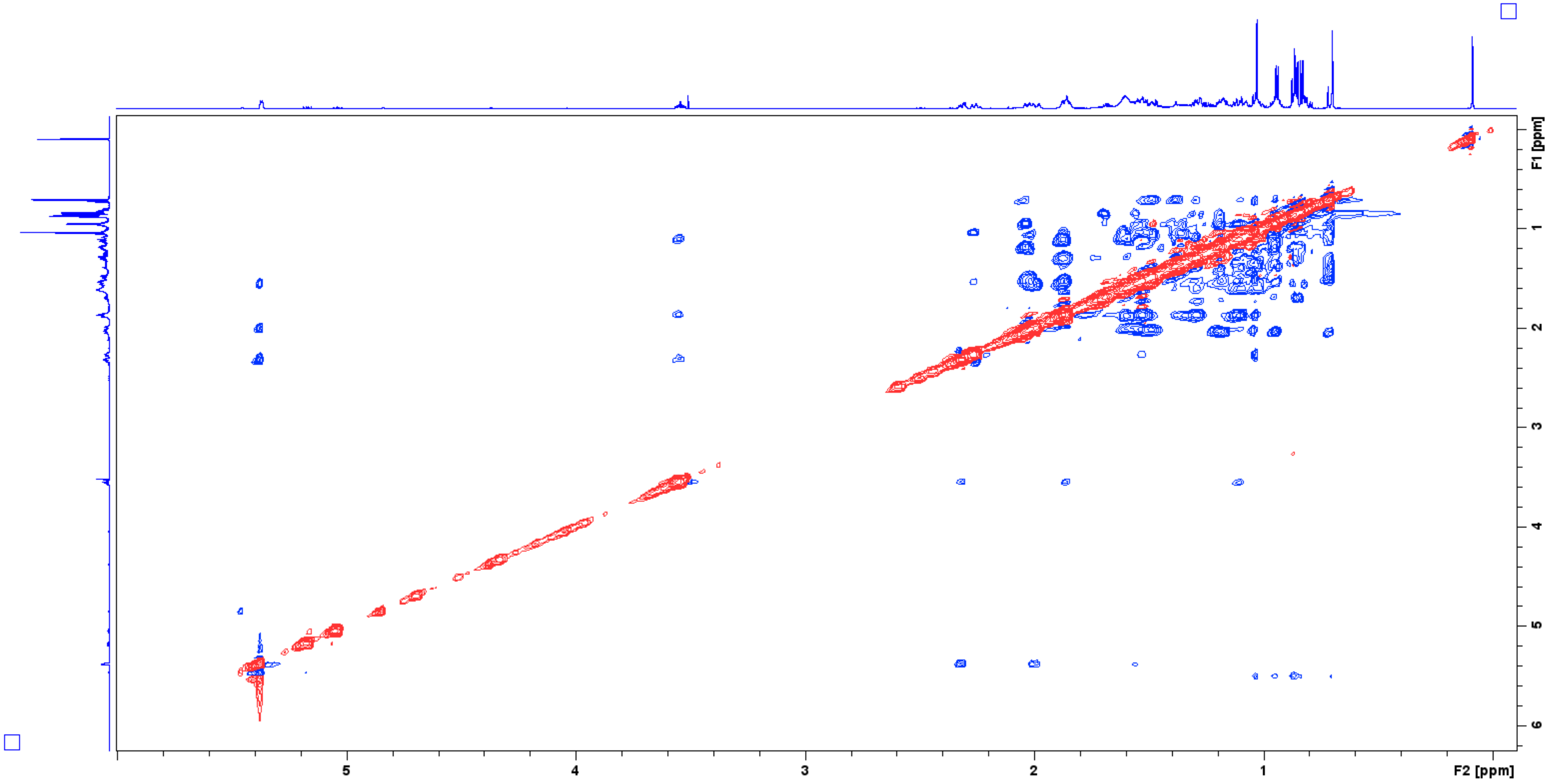

Figure S67. ROESY NMR spectrum of β-sitosterol (**10**) in $CDCl_3$ (700 MHz)

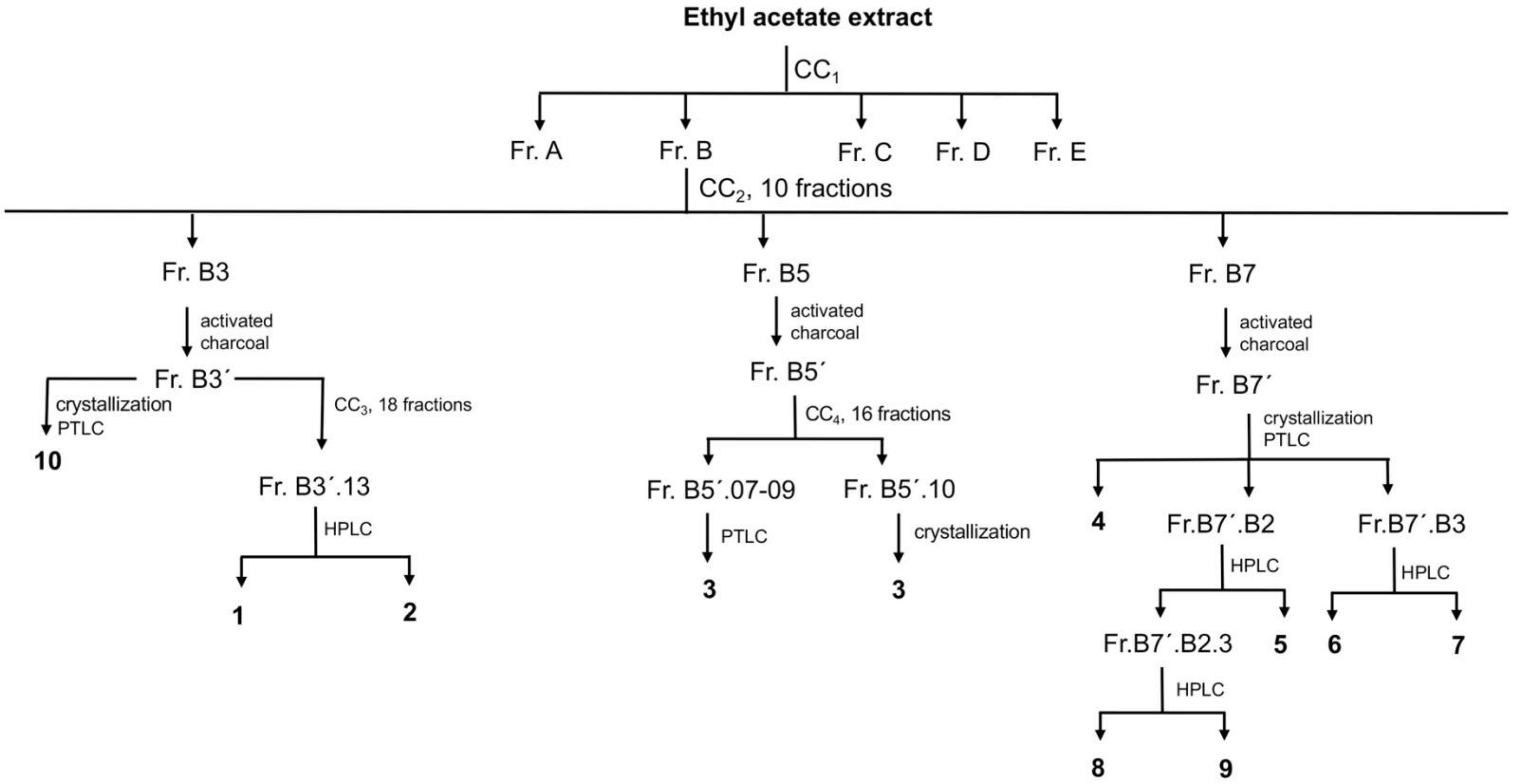

Scheme S1. Overview of isolation procedures
